# Supplementary material for: Development of a High-Resolution Single-Nucleotide Polymorphism Strain-Typing Assay Using Whole Genome-Based Analyses for the Lactobacillus acidophilus Probiotic Strain
Source: Microorganisms. 2020 Sep 21;8(9):1445. doi: 10.3390/microorganisms8091445 (PMC7564606; doi:10.3390/microorganisms8091445)
Supplement: Supplementary file 1 [file microorganisms-08-01445-s001.pdf]

Supplementary Table S1. Genomic characteristics of *Lactobacillus acidophilus* strains

Strain	Other designation	Source	Accession no.	Genome size (bp)	G+C content (%)	No. of contigs	N50 length (bp)	No. of CDS	rRNAs	tRNAs
**BCRC 12255**	NCIMB 701243	Cheese starter	JAAONQ000000000	1,969,483	34.6	26	167,756	1,850	7	60
**BCRC 14065**	CSCC 2401	Commercial probiotics	JAAONR000000000	1,967,267	34.6	17	231,484	1,853	3	56
**BCRC 14079**		Yogurt	JAAONS000000000	1,987,806	34.5	21	167,722	1,881	6	58
**BCRC 16092**	CCUG 12853	Acidophilus milk	JAAONT000000000	1,950,144	34.5	20	167,711	1,870	4	60
**BCRC 16099**	CIP 103600	Acidophilus milk	JAAOXP000000000	1,966,633	34.6	3	231,484	1,851	3	56
**BCRC 17008**	ATCC 4357	Human	JAAOXQ000000000	1,977,848	34.6	17	222,101	1,867	4	56
**BCRC 17481**	JCM 1028	Human	JAAOXR000000000	1,993,969	34.6	18	167,771	1,873	7	61
**BCRC 17486**	JCM 1229	Human	JAAPAR000000000	2,003,593	34.5	24	166,801	1,949	6	58
**BCRC 80064**		Human	JAAPAS000000000	1,961,821	34.5	22	167,795	1,849	4	60
ATCC 4796			GCA_000159715	2,020,500	34.2	38	1,227,701	1,957	4	58
ATCC 53544	BCRC 17009	Feed supplement	GCA_002224305	1,991,906	34.7	1	1,991,906	1,867	12	61
DSM 9126	ATCC 13651		GCA_000469745	1,991,758	34.5	27	242,633	1,893	4	44
DSM 20242			GCA_000442825	2,047,860	34.7	21	169,267	1,921	13	57
DSM 20079^T	BCRC 10695^T	Human	GCA_003047065	2,009,973	34.7	1	2,009,973	1,919	12	60
**LA1063**		Dietary supplement	JAAUOF000000000	1,995,761	34.6	14	242,274	1,874	4	56
CIRM-BIA 442		Dairy product	GCA_000442865	1,986,992	34.7	19	169,301	1,867	10	58
CIRM-BIA 445		Dairy product	GCA_000469765	2,002,008	34.5	22	242,633	1,893	4	14
DS1_1A		Commercial dietary supplement	GCA_003062045	1,970,142	34.6	36	168,008	1,851	5	61
DS10_1A		Commercial dietary supplement	GCA_003053245	1,970,718	34.6	34	167,634	1,825	5	61
DS11_1A		Commercial dietary supplement	GCA_003062025	1,968,514	34.6	34	168,008	1,851	5	61
DS13_1A		Commercial dietary supplement	GCA_003061965	1,964,272	34.6	41	168,388	1,853	3	61
DS13_1B		Commercial dietary supplement	GCA_003061905	1,963,932	34.6	37	167,831	1,851	3	61
DS2_1A		Commercial dietary supplement	GCA_003062005	1,982,025	34.6	110	86,217	1,855	16	61
DS20_1		Commercial dietary supplement	GCA_003061885	1,969,467	34.6	36	167,716	1,850	5	61
DS24_1		Commercial dietary supplement	GCA_003053135	1,968,373	34.6	32	168,151	1,851	5	61
DS5_1A		Commercial dietary supplement	GCA_003061985	1,968,830	34.6	28	167,756	1,851	5	61
DS8_1A		Commercial dietary supplement	GCA_003061945	1,967,572	34.6	32	167,594	1,851	5	61
DS9_1A		Commercial dietary supplement	GCA_003061925	1,969,008	34.6	28	167,756	1,852	5	61
FSI4		Yogurt	GCA_000934625	1,991,969	34.7	1	1,991,969	1,863	12	61
L-55		Commercial dairy product	GCA_001950045	2,009,507	34.6	25	242,365	1,902	4	54
LA1		Dairy product	GCA_002286215	1,991,195	34.7	1	1,991,195	1,864	12	61
La-14		Dietary supplement	GCA_000389675	1,991,579	34.7	1	1,991,579	1,869	12	61
LA-5		Dietary supplement	JAAQQS000000000	1,965,390	34.6	19	231,909	1,856	3	57
LMG P-21904		Commercial dietary supplement	GCA_002914945	1,965,665	34.6	16	320,101	1,853	3	58
MGYG-HGUT-02379		Human	GCA_902386525	2,009,507	34.6	25	242,365	1,902	4	54
NCFM		Dietary supplement	GCA_000011985	1,993,560	34.7	1	1,993,560	1,877	12	61
P2		Commercial probiotic	GCA_002406675	2,046,837	35.7	70	167,927	1,923	12	61
PNW3		Pig	GCA_004348805	1,857,655	34.6	25	230,557	1,757	0	55
UBLA-34		Commercial dietary supplement	GCA_003641085	1,951,037	34.6	34	167,656	1,845	3	60
WG-LB-IV		Yogurt	GCA_001639165	1,951,689	34.6	74	89,054	1,819	4	60
YT1		Intestinal tract	GCA_003952845	2,092,540	34.7	1	2,092,540	1,985	15	65

The bold letters indicate the strains which were sequenced in this study.

Supplementary Table S2. Genes and primers used for validation of reference strains.

Gene	Enzyme function	Primer	Sequence (5'–3')	Amplicon size (bp)
oppA	Oligopeptide ABC transporter substrate-binding protein	SAL0000007-F	AACCTGGTATGAATACTAG	1167
		SAL0000007-R	TAGCGTAAGCAACATTGTAC	
tr	Transcriptional regulator	SAL0000541-F	TAAGATGGCAATTAAGGAC	397
		SAL0000541-R	ACTAATGCAATACCAG	
ybhL	Inner membrane protein YbhL	SAL0000678-R	TCCTGAGCGTCGTCAAGTA	663
		SAL0000678-R	ACGATCATTACCCATACCA	
frdA	Fumarate reductase flavoprotein subunit	SAL0001061-F	CGTCAAGACGGTAGCAAGAT	115
		SAL0001061-R	TCCAGGCTTGAGCCTGACCA	
hp	hypothetical protein	SAL0001150-F	TACAATGTGGACATGAGYGG	675
		SAL0001150-R	AAGTTAAGACCAGATAAGTT	
rr	Response regulator	SAL0001160-F	TGAAGATGACAACTCCGTTG	273
		SAL0001160-R	GCTCCAATACCAAGACCTAC	
uvrA _2	UvrABC system protein A	SAL0001385-F	ACCATATCTTGATGGTAGTG	358
		SAL0001385-R	ACGCCGCAAGTTATTCATAA	
phoU	Phosphate-specific transport system accessory protein	SAL0001390-F	CATGATTATGTTCGTGCTAGA	165
		SAL0001390-R	GCTAAGTTAACAATGTGATCGC	

Supplementary Table S3. List of the 1390 cgMLST loci in 41 *Lactobacillus acidophilus* strains.

Locus	Gene	Annotation	Allele profile																																							
			BCRC 12255	BCRC 14065	BCRC 14079	BCRC 16092	BCRC 16099	BCRC 17008	BCRC 17481	BCRC 17486	BCRC 80064	LA1063	LA-5	ATCC 4796	ATCC 53544	CIRM-BIA-442	CIRM-BIA-445	DS10_1A	DS11_1A	DS13_1A	DS13_1B	DS1_1A	DS20_1	DS24_1	DS2_1A	DS5_1A	DS8_1A	DS9_1A	DSM 20079T	DSM 20242	DSM 9126	MGYG-HGUT-02379	FSI4	L-55	LA1	LMG P-21904	La-14	NCFM	P2	PNW3	UBLA-34	WG-LB-IV	YT1
SAL0000001	kup	potassium transport protein Kup	1	1	3	2	1	4	2	9	1	2	1	2	1	1	3	1	1	1	1	1	1	1	1	1	1	1	2	5	8	3	1	3	1	1	1	7	1	1	1	1	6
SAL0000002	lmrA	Multidrug resistance ABC transporter ATP-binding and permease protein	1	1	1	2	1	1	1	2	1	1	1	1	1	1	1	1	1	1	1	1	1	1	1	1	1	1	2	1	1	1	1	1	1	1	1	1	1	1	1	1	3
SAL0000003	maa_2	Maltose O-acetyltransferase	2	2	2	2	2	2	2	2	2	2	2	3	2	2	2	2	2	2	2	2	2	2	2	2	2	2	2	4	2	2	2	2	2	2	2	2	2	2	2	2	5
SAL0000004	hp	hypothetical protein	1	1	1	2	1	1	1	3	1	1	1	1	1	1	1	1	1	1	1	1	1	1	1	1	1	1	2	1	1	1	1	1	1	1	1	1	1	1	1	1	4
SAL0000005	group_127	type I restriction enzyme EcoKI subunit R	1	1	1	1	1	6	1	4	1	1	1	2	1	1	5	1	1	1	1	1	1	1	1	1	1	1	1	1	1	1	1	1	1	1	1	1	1	1	1	1	3
SAL0000006	group_13	DegV domain-containing protein	1	1	2	3	1	1	1	1	1	1	1	6	1	1	2	1	1	1	1	1	1	1	1	1	1	1	3	1	6	2	1	2	1	1	1	1	1	1	1	1	4
**SAL0000007**	**oppA_1**	**Oligopeptide-binding protein AppA precursor**	1	1	2	4	1	11	3	10	1	3	1	6	1	1	2	1	1	1	7	1	1	1	1	1	1	1	4	8	5	2	1	2	1	1	1	1	1	1	9	1	5
SAL0000008	yflS	Putative malate transporter YflS	1	1	1	1	1	1	1	2	1	1	1	3	1	1	1	1	1	1	1	1	1	1	1	1	1	1	1	2	4	1	1	1	1	1	1	1	1	1	1	1	5
SAL0000009	recQ	ATP-dependent DNA helicase RecQ	1	1	1	1	1	1	1	3	1	1	1	1	1	1	1	1	1	1	1	1	1	1	1	1	1	1	1	1	1	1	1	1	1	1	1	1	1	1	1	1	2
SAL0000010	ycaM_2	Inner membrane transporter YcaM	1	1	1	1	1	1	1	1	1	1	1	2	1	1	1	1	1	1	1	1	1	1	1	1	1	1	1	1	1	1	1	1	1	1	1	1	1	1	1	1	3
SAL0000011	group_1517	Bacterial ABC transporter protein EcsB	1	1	1	1	1	1	1	1	1	1	1	1	1	1	1	1	1	1	1	1	1	1	1	1	1	1	1	1	1	1	1	1	1	1	1	1	1	1	1	1	1
SAL0000012	rpmD	50S ribosomal protein L30	1	1	1	1	1	1	1	1	1	1	1	1	1	1	1	1	1	1	1	1	1	1	1	1	1	1	1	1	1	1	1	1	1	1	1	1	1	1	1	1	1
SAL0000013	yigZ	IMPACT family member YigZ	1	1	1	1	1	1	1	1	1	1	1	1	1	1	1	1	1	1	1	1	1	1	1	1	1	1	1	1	1	1	1	1	1	1	1	1	1	1	1	1	1
SAL0000014	pstA	Phosphate transport system permease protein PstA	1	1	1	1	1	1	1	1	1	1	1	1	1	1	1	1	1	1	1	1	1	1	1	1	1	1	1	1	1	1	1	1	1	1	1	1	1	1	1	1	1
SAL0000015	group_1521	rod shape-determining protein MreD	1	1	1	1	1	1	1	1	1	1	1	1	1	1	1	1	1	1	1	1	1	1	1	1	1	1	1	1	1	1	1	1	1	1	1	1	1	1	1	1	1
SAL0000016	ydjP	AB hydrolase superfamily protein YdjP	1	1	1	1	1	1	1	1	1	1	1	1	1	1	1	1	1	1	1	1	1	1	1	1	1	1	1	1	1	1	1	1	1	1	1	1	1	1	1	1	1
SAL0000017	xerD	Tyrosine recombinase XerD	1	1	1	1	1	1	1	1	1	1	1	1	1	1	1	1	1	1	1	1	1	1	1	1	1	1	1	1	1	1	1	1	1	1	1	1	1	1	1	1	2
SAL0000018	oppD_1	Oligopeptide transport ATP-binding protein OppD	1	1	1	1	1	1	1	1	1	1	1	1	1	1	1	1	1	1	1	1	1	1	1	1	1	1	1	1	1	1	1	1	1	1	1	1	1	1	1	1	1
SAL0000019	group_1525	transcriptional regulator SlyA	1	1	1	1	1	1	1	1	1	1	1	1	1	1	1	1	1	1	1	1	1	1	1	1	1	1	1	1	1	1	1	1	1	1	1	1	1	1	1	1	1
SAL0000020	hp	hypothetical protein	1	1	1	1	1	1	1	1	1	1	1	1	1	1	1	1	1	1	1	1	1	1	1	1	1	1	1	1	1	1	1	1	1	1	1	2	1	1	1	1	1
SAL0000021	rplS	50S ribosomal protein L19	1	1	1	1	1	1	1	1	1	1	1	1	1	1	1	1	1	1	1	1	1	1	1	1	1	1	1	1	1	1	1	1	1	1	1	1	1	1	1	1	1
SAL0000022	group_1528	sensory histidine kinase DcuS	1	1	1	1	1	1	1	1	1	1	1	1	1	1	1	1	1	1	1	1	1	1	1	1	1	1	1	1	1	1	1	1	1	1	1	1	1	1	1	1	1
SAL0000023	hp	hypothetical protein	1	1	1	1	1	1	1	1	1	1	1	1	1	1	1	1	1	1	1	1	1	1	1	1	1	1	1	1	1	1	1	1	1	1	1	1	1	1	1	1	2
SAL0000024	yvgN_2	putative oxidoreductase/MSMEI_2347	1	1	3	2	1	1	1	1	1	1	1	1	1	1	1	1	1	1	1	1	1	1	1	1	4	1	2	1	1	1	1	1	1	1	1	1	1	1	1	1	5
SAL0000025	dut	Deoxyuridine 5'-triphosphate nucleotidohydrolase	1	1	1	1	1	1	1	1	1	1	1	1	1	1	1	1	1	1	1	1	1	1	1	1	1	1	1	1	1	1	1	1	1	1	1	1	1	1	1	1	2
SAL0000026	group_1531	Enterocin A Immunity	1	1	1	1	1	1	1	2	1	1	1	1	1	1	1	1	1	1	1	1	1	1	1	1	1	1	1	1	1	1	1	1	1	1	1	1	1	1	1	1	1
SAL0000027	recO	DNA repair protein RecO	1	1	1	1	1	1	1	1	1	1	1	1	1	1	1	1	1	1	1	1	1	1	1	1	1	1	1	1	1	1	1	1	1	1	1	1	1	1	1	1	1
SAL0000028	hp	hypothetical protein	1	1	1	1	1	1	1	1	1	1	1	1	1	1	1	1	1	1	1	1	1	1	1	1	1	1	1	1	1	1	1	1	1	1	1	1	1	1	1	1	1
SAL0000029	hp	hypothetical protein	1	1	1	1	1	1	1	1	1	1	1	1	1	1	1	1	1	1	1	1	1	1	1	1	1	1	1	1	1	1	1	1	1	1	1	1	1	1	1	1	1
SAL0000030	rpmF	50S ribosomal protein L32	1	1	1	1	1	1	1	1	1	1	1	1	1	1	1	1	1	1	1	1	1	1	1	1	1	1	1	1	1	1	1	1	1	1	1	1	1	1	1	1	1
SAL0000031	group_1536	camphor resistance protein CrcB	1	1	1	1	1	1	1	1	1	1	1	1	1	1	1	1	1	1	1	1	1	1	1	1	1	1	1	1	1	1	1	1	1	1	1	1	1	1	1	1	2
SAL0000032	ribF	Riboflavin biosynthesis protein RibF	1	1	1	1	1	1	1	1	1	1	1	1	1	1	1	1	1	1	1	1	1	1	1	1	1	1	1	1	1	1	1	1	1	1	1	1	1	1	1	1	1
SAL0000033	rluB_1	Ribosomal large subunit pseudouridine synthase B	1	1	1	1	1	1	1	1	1	1	1	1	1	1	1	1	1	1	1	1	1	1	1	1	1	1	1	1	1	1	1	1	1	1	1	1	1	1	1	1	1
SAL0000034	yttA	putative membrane protein YttA	1	1	1	1	1	1	1	1	1	1	1	1	1	1	1	1	1	1	1	1	1	1	1	1	1	1	1	1	1	1	1	1	1	1	1	1	1	1	1	1	1
SAL0000035	hp	hypothetical protein	1	1	1	1	1	1	1	1	1	1	1	1	1	1	1	1	1	1	1	1	1	1	1	1	1	1	1	1	1	1	1	1	1	1	1	1	1	1	1	1	2
SAL0000036	hp	hypothetical protein	1	1	1	1	1	1	1	1	1	1	1	1	1	1	1	1	1	1	1	1	1	1	1	1	1	1	1	1	1	1	1	1	1	1	1	1	1	1	1	1	1
SAL0000037	group_1542	Enterocin A Immunity	1	1	1	1	1	1	1	1	1	1	1	1	1	1	1	1	1	1	1	1	1	1	1	1	1	1	1	1	1	1	1	1	1	1	1	1	1	1	1	1	1
SAL0000038	purQ	Phosphoribosylformylglycinamidine synthase 1	1	1	1	1	1	1	1	1	1	1	1	1	1	1	1	1	1	1	1	1	1	1	1	1	1	1	1	1	1	1	1	1	1	1	1	1	1	1	1	1	1
SAL0000039	tnpR	Transposon Tn3 resolvase	1	1	1	1	1	1	1	1	1	1	1	1	1	1	1	1	1	1	1	1	1	1	1	1	1	1	1	1	1	1	1	1	1	1	1	1	1	1	1	1	2
SAL0000040	purA	Adenylosuccinate synthetase	1	1	1	2	1	1	1	1	1	1	1	1	1	1	1	1	1	1	1	1	1	1	1	1	1	1	2	1	1	1	1	1	1	1	1	1	1	1	1	1	1
SAL0000041	group_1546	Zeta toxin	1	1	1	1	1	1	1	1	1	1	1	1	1	1	1	1	1	1	1	1	1	1	1	1	1	1	1	1	1	1	1	1	1	1	1	1	1	1	1	1	1
SAL0000042	hp	hypothetical protein	1	1	1	1	1	1	1	1	1	1	1	1	1	1	1	1	1	1	1	1	1	1	1	1	1	1	1	1	1	1	1	1	1	1	1	1	1	1	1	1	1
SAL0000043	def2_1	Peptide deformylase 2	1	1	1	1	1	1	1	1	1	1	1	1	1	1	1	1	1	1	1	1	1	1	1	1	1	1	1	1	1	1	1	1	1	1	1	1	1	1	1	1	1
SAL0000044	ruvB	Holliday junction ATP-dependent DNA helicase RuvB	1	1	1	1	1	1	1	1	1	1	1	1	1	1	1	1	1	1	1	1	1	1	1	1	1	1	1	1	1	1	1	1	1	1	1	1	1	1	1	1	2
SAL0000045	group_1552	guanine deaminase	1	1	1	1	1	1	1	1	1	1	1	1	1	1	1	1	1	1	1	1	1	1	1	1	1	1	1	1	1	1	1	1	1	1	1	1	1	1	1	1	2
SAL0000046	group_1553	ABC transporter transmembrane region	1	1	1	1	1	1	1	1	1	1	1	1	1	1	1	1	1	1	1	1	1	1	1	1	1	1	1	1	1	1	1	1	1	1	1	1	1	1	1	1	2
SAL0000047	group_1554	Phosphorylated carbohydrates phosphatase	1	1	1	1	1	1	1	1	1	1	1	1	1	1	1	1	1	1	1	1	1	1	1	1	1	1	1	1	1	1	1	1	1	1	1	1	1	1	1	1	1
SAL0000048	group_1555	Putative GTP cyclohydrolase 1 type 2	1	1	1	1	1	1	1	1	1	1	1	1	1	1	1	1	1	1	1	1	1	1	1	1	1	1	1	1	1	1	1	1	1	1	1	3	1	1	1	1	2
SAL0000049	hp	hypothetical protein	1	1	1	1	1	1	1	1	1	1	1	1	1	1	1	1	1	1	1	1	1	1	1	1	1	1	1	1	1	1	1	1	1	1	1	1	1	1	1	1	1
SAL0000050	nlhH_1	Carboxylesterase NlhH	1	1	1	1	1	1	1	1	1	1	1	1	1	1	1	1	1	1	1	1	1	1	1	1	1	1	1	1	1	1	1	1	1	1	1	1	1	1	1	1	1
SAL0000051	yjjG	Pyrimidine 5'-nucleotidase YjjG	1	1	1	1	1	1	1	1	1	1	1	1	1	1	1	1	1	1	1	1	1	1	1	1	1	1	1	1	1	1	1	1	1	1	1	1	1	1	1	1	1
SAL0000052	glvR_3	HTH-type transcriptional regulator GlvR	1	1	1	1	1	1	1	1	1	1	1	1	1	1	1	1	1	1	1	1	1	1	1	1	1	1	1	1	1	1	1	1	1	1	1	1	1	1	1	1	2
SAL0000053	group_1560	Phd_YefM	1	1	1	1	1	1	1	1	1	1	1	1	1	1	1	1	1	1	1	1	1	1	1	1	1	1	1	1	1	1	1	1	1	1	1	1	1	1	1	1	1
SAL0000054	rimM	Ribosome maturation factor RimM	1	1	1	1	1	1	1	1	1	1	1	1	1	1	1	1	1	1	1	1	1	1	1	1	1	1	1	1	1	1	1	1	1	1	1	1	1	1	1	1	1
SAL0000055	infA	Translation initiation factor IF-1	1	1	1	1	1	1	1	1	1	1	1	1	1	1	1	1	1	1	1	1	1	1	1	1	1	1	1	1	1	1	1	1	1	1	1	1	1	1	1	1	1
SAL0000056	group_1563	Pyridoxamine 5'-phosphate oxidase	1	1	1	1	1	1	1	1	1	1	1	1	1	1	1	1	1	1	1	1	1	1	1	1	1	1	1	1	1	1	1	1	1	1	1	1	1	1	1	1	2

SAL0000057	tagO	putative undecaprenyl-phosphate N-acetylglucosaminyl 1-phosphate transfer	1	1	1	1	1	1	1	1	1	1	1	1	1	1	1	1	1	1	1	1	1	1	1	1	1	1	1	1	1	1	1	1	1	1	1	1	1	1	1	1	1
SAL0000058	arlR	Response regulator ArlR	1	1	1	1	1	1	1	1	1	1	1	1	1	1	1	1	1	1	1	1	1	1	1	1	1	1	1	1	1	1	1	1	1	1	1	1	1	1	1	1	2
SAL0000059	rluB_2	Ribosomal large subunit pseudouridine synthase B	1	1	1	1	1	1	1	1	1	1	1	1	1	1	1	1	1	1	1	1	1	1	1	1	1	1	1	1	1	1	1	1	1	1	1	1	1	1	1	1	1
SAL0000060	rpsH	30S ribosomal protein S8	1	1	1	1	1	1	1	1	1	1	1	1	1	1	1	1	1	1	1	1	1	1	1	1	1	1	1	1	1	1	1	1	1	1	1	1	1	1	1	1	1
SAL0000061	hp	hypothetical protein	1	1	1	1	1	1	1	1	1	1	1	1	1	1	1	1	1	1	1	1	1	1	1	1	1	1	1	1	1	1	1	1	1	1	1	1	1	1	1	1	1
SAL0000062	adhR	HTH-type transcriptional regulator AdhR	1	1	1	1	1	1	1	1	1	1	1	1	1	1	1	1	1	1	1	1	1	1	1	1	1	1	1	1	1	1	1	1	1	1	1	1	1	1	1	1	2
SAL0000063	group_157	DegV domain-containing protein	1	1	2	3	6	1	5	7	1	5	1	4	1	6	2	1	1	1	1	1	1	1	1	1	1	1	3	9	4	2	1	2	8	1	1	1	1	1	1	1	1
SAL0000064	plsX	Phosphate acyltransferase	1	1	1	1	1	1	1	1	1	1	1	2	1	1	1	1	1	1	1	1	1	1	1	1	1	1	1	1	1	1	1	1	1	1	1	1	1	1	1	1	1
SAL0000065	artQ_2	Arginine transport system permease protein ArtQ	1	1	1	1	1	1	1	1	1	1	1	1	1	1	1	1	1	1	1	1	1	1	1	1	1	1	1	1	1	1	1	1	1	1	1	1	1	1	1	1	2
SAL0000066	group_1572	putative DNA-binding protein	1	1	1	1	1	1	1	1	1	1	1	1	1	1	1	1	1	1	1	1	1	1	1	1	1	1	1	1	1	1	1	1	1	1	1	1	1	1	1	1	1
SAL0000067	murR	HTH-type transcriptional regulator MurR	1	1	1	1	1	1	1	1	1	1	1	1	1	1	1	1	1	1	1	1	1	1	1	1	1	1	1	1	1	1	1	1	1	1	1	2	1	1	1	1	1
SAL0000068	glnM	putative glutamine ABC transporter permease protein GlnM	1	1	1	1	1	1	1	1	1	1	1	1	1	1	1	1	1	1	1	1	1	1	1	1	1	1	1	1	1	1	1	1	1	1	1	1	1	1	1	1	2
SAL0000069	hp	hypothetical protein	1	1	1	1	1	1	1	1	1	1	1	1	1	1	1	1	1	1	1	1	1	1	1	1	1	1	1	1	1	1	1	1	1	1	1	1	1	1	1	1	2
SAL0000070	rplT	50S ribosomal protein L20	1	1	1	1	1	1	1	1	1	1	1	1	1	1	1	1	1	1	1	1	1	1	1	1	1	1	1	1	1	1	1	1	1	1	1	1	1	1	1	1	1
SAL0000071	group_1577	Putative peptidoglycan binding domain protein	1	1	1	1	1	1	1	1	1	1	1	1	1	1	1	1	1	1	1	1	1	1	1	1	1	1	1	1	1	1	1	1	1	1	1	1	1	1	1	1	2
SAL0000072	folD	Bifunctional protein FolD protein	1	1	1	1	1	1	1	1	1	1	1	1	1	1	1	1	1	1	1	1	1	1	1	1	1	1	1	1	1	1	1	1	1	1	1	1	1	1	1	1	1
SAL0000073	hp	hypothetical protein	1	1	1	1	1	1	1	1	1	1	1	1	1	1	1	1	1	1	1	1	1	1	1	1	1	1	1	1	1	1	1	1	1	1	1	1	1	1	1	1	2
SAL0000074	ftsY	Signal recognition particle receptor FtsY	1	1	1	1	1	1	1	1	1	1	1	1	1	1	1	1	1	1	1	1	1	1	1	1	1	1	1	1	1	1	1	1	1	1	1	1	1	1	1	1	1
SAL0000075	rpmI	50S ribosomal protein L35	1	1	1	1	1	1	1	1	1	1	1	1	1	1	1	1	1	1	1	1	1	1	1	1	1	1	1	1	1	1	1	1	1	1	1	1	1	1	1	1	1
SAL0000076	greA_2	Transcription elongation factor GreA	1	1	1	1	1	1	1	1	1	1	1	1	1	1	1	1	1	1	1	1	1	1	1	1	1	1	1	1	1	1	1	1	1	1	1	1	1	1	1	1	2
SAL0000077	pip	Proline iminopeptidase	1	1	1	1	1	1	1	1	1	1	1	1	1	1	1	1	1	1	1	1	1	1	1	1	1	1	1	1	1	1	1	1	1	1	1	1	1	1	1	1	2
SAL0000078	truB	tRNA pseudouridine synthase B	1	1	1	1	1	1	1	1	1	1	1	1	1	1	1	1	1	1	1	1	1	1	1	1	1	1	1	1	1	1	1	1	1	1	1	1	1	1	1	1	1
SAL0000079	rimP	Ribosome maturation factor RimP	1	1	1	1	1	1	1	1	1	1	1	1	1	1	1	1	1	1	1	1	1	1	1	1	1	1	1	1	1	1	1	1	1	1	1	1	1	1	1	1	1
SAL0000080	yciB_1	Putative L,D-transpeptidase YciB precursor	1	1	1	1	1	1	1	1	1	1	1	1	1	1	1	1	1	1	1	1	1	1	1	1	1	1	1	1	1	1	1	1	1	1	1	1	1	1	1	1	1
SAL0000081	group_1587	Putative peptidyl-prolyl cis-trans isomerase	1	1	2	1	1	1	1	1	1	1	1	1	1	1	1	1	1	1	1	1	1	1	1	1	1	1	1	1	1	1	1	1	1	1	1	1	1	1	1	1	1
SAL0000082	group_1588	phosphatidylglycerophosphatase B	1	1	1	1	1	1	1	1	1	1	1	1	1	1	1	1	1	1	1	1	1	1	1	1	1	1	1	1	1	1	1	1	1	1	1	1	1	1	1	1	1
SAL0000083	rplU	50S ribosomal protein L21	1	1	1	1	1	1	1	1	1	1	1	1	1	1	1	1	1	1	1	1	1	1	1	1	1	1	1	1	1	1	1	1	1	1	1	1	1	1	1	1	1
SAL0000084	hp	hypothetical protein	1	1	3	1	1	1	1	1	1	1	1	4	1	1	1	1	1	1	1	1	1	1	1	1	1	1	1	1	1	1	1	1	1	1	1	1	1	1	1	1	2
SAL0000085	glvR_1	HTH-type transcriptional regulator GlvR	1	1	1	1	1	1	1	1	1	1	1	1	1	1	1	1	1	1	1	1	1	1	1	1	1	1	1	1	1	1	1	1	1	1	1	1	1	1	1	1	2
SAL0000086	rplN	50S ribosomal protein L14	1	1	1	1	1	1	1	1	1	1	1	1	1	1	1	1	1	1	1	1	1	1	1	1	1	1	1	1	1	1	1	1	1	1	1	1	1	1	1	1	1
SAL0000087	yjbC	Putative acetyltransferase YjbC	1	1	1	1	1	1	1	1	1	1	1	1	1	1	1	1	1	1	1	1	1	1	1	1	1	1	1	1	1	1	1	1	1	1	1	1	1	1	1	1	1
SAL0000088	hp	hypothetical protein	1	1	1	1	1	1	1	2	1	1	1	1	1	1	1	1	1	1	1	1	1	1	1	1	1	1	1	1	1	1	1	1	1	1	1	1	1	1	1	1	1
SAL0000089	pepDA_4	Dipeptidase A	1	1	1	1	1	1	1	2	1	1	1	1	1	1	1	1	1	1	1	1	1	1	1	1	1	1	1	1	1	1	1	1	1	1	1	1	1	1	1	1	1
SAL0000090	hp	hypothetical protein	1	1	1	1	1	1	1	1	1	1	1	1	1	1	1	1	1	1	1	1	1	1	1	1	1	1	1	1	1	1	1	1	1	1	1	1	1	1	1	1	1
SAL0000091	hp	hypothetical protein	1	1	1	1	1	1	1	1	1	1	1	1	1	1	1	1	1	1	1	1	1	1	1	1	1	1	1	1	1	1	1	1	1	1	1	1	1	1	1	1	1
SAL0000092	group_1597	Enterocin A Immunity	1	1	1	1	1	1	1	1	1	1	1	1	1	1	1	1	1	1	1	1	1	1	1	1	1	1	1	1	1	1	1	1	1	1	1	1	1	1	1	1	1
SAL0000093	smpB	SsrA-binding protein	1	1	1	1	1	1	1	1	1	1	1	1	1	1	1	1	1	1	1	1	1	1	1	1	1	1	1	1	1	1	1	1	1	1	1	1	1	1	1	1	1
SAL0000094	group_1599	CYTH domain protein	1	1	1	1	1	1	1	1	1	1	1	1	1	1	1	1	1	1	1	1	1	1	1	1	1	1	1	1	1	1	1	1	1	1	1	1	1	1	1	1	1
SAL0000095	hp	hypothetical protein	1	1	1	1	1	1	1	1	1	1	1	1	1	1	1	1	1	1	1	1	1	1	1	1	1	1	1	1	1	1	1	1	1	1	1	1	1	1	1	1	1
SAL0000096	pgsA	CDP-diacylglycerol--glycerol-3-phosphate 3-phosphatidyltransferase	1	1	1	1	1	1	1	1	1	1	1	1	1	1	1	1	1	1	1	1	1	1	1	1	1	1	1	1	1	1	1	1	1	1	1	1	1	1	1	1	1
SAL0000097	butA	Aminoglycoside 3'-phosphotransferase	1	1	1	1	1	1	1	1	1	1	1	1	1	1	1	1	1	1	1	1	1	1	1	1	1	1	1	1	1	1	1	1	1	1	1	1	1	1	1	1	1
SAL0000098	rplQ	50S ribosomal protein L17	1	1	1	1	1	1	1	1	1	1	1	1	1	1	1	1	1	1	1	1	1	1	1	1	1	1	1	1	1	1	1	1	1	1	1	1	1	1	1	1	1
SAL0000099	hp	hypothetical protein	1	1	1	1	1	1	1	1	1	1	1	1	1	1	1	1	1	1	1	1	1	1	1	1	1	1	1	1	1	1	1	1	1	1	1	1	1	1	1	1	1
SAL0000100	rpsQ	30S ribosomal protein S17	1	1	1	1	1	1	1	1	1	1	1	1	1	1	1	1	1	1	1	1	1	1	1	1	1	1	1	1	1	1	1	1	1	1	1	1	1	1	1	1	1
SAL0000101	potA	Spermidine/putrescine import ATP-binding protein PotA	1	1	1	1	1	1	1	1	1	1	1	2	1	1	1	1	1	1	1	1	1	1	1	1	1	1	1	1	1	1	1	1	1	1	1	1	1	1	1	1	3
SAL0000102	patA_2	Putative N-acetyl-LL-diaminopimelate aminotransferase	1	1	1	1	1	1	1	2	1	1	1	1	1	1	3	1	1	1	1	1	1	1	1	1	1	1	1	4	1	1	1	1	1	1	1	1	1	1	1	1	1
SAL0000103	group_1610	cobyric acid synthase	1	1	1	1	1	1	1	1	1	1	1	1	1	1	1	1	1	1	1	1	1	1	1	1	1	1	1	1	1	1	1	1	1	1	1	1	1	1	1	1	2
SAL0000104	rpsZ_2	30S ribosomal protein S14 type Z	1	1	1	1	1	1	1	1	1	1	1	1	1	1	1	1	1	1	1	1	1	1	1	1	1	1	1	1	1	1	1	1	1	1	1	1	1	1	1	1	1
SAL0000105	rplK	50S ribosomal protein L11	1	1	1	1	1	1	1	1	1	1	1	1	1	1	1	1	1	1	1	1	1	1	1	1	1	1	1	1	1	1	1	1	1	1	1	1	1	1	1	1	1
SAL0000106	group_1613	RelB antitoxin	1	1	1	1	1	1	1	1	1	1	1	1	1	1	1	1	1	1	1	1	1	1	1	1	1	1	1	1	1	1	1	1	1	1	1	1	1	1	1	1	1
SAL0000107	fusA	Elongation factor G	1	1	1	1	1	1	1	1	1	1	1	1	1	1	1	1	1	1	1	1	1	1	1	1	1	1	1	1	1	1	1	1	1	1	1	1	1	1	1	1	1
SAL0000108	era	GTPase Era	1	1	1	1	1	1	1	1	1	1	1	1	1	1	1	1	1	1	1	1	1	1	1	1	1	1	1	1	1	1	1	1	1	1	1	1	1	1	1	1	1
SAL0000109	parE	DNA topoisomerase 4 subunit B	1	1	1	1	1	3	1	2	1	1	1	1	1	1	1	1	1	1	1	1	1	1	1	1	1	1	1	2	1	1	1	1	1	1	1	1	1	1	1	1	1
SAL0000110	pspB_1	Putative phosphoserine phosphatase 2	1	1	1	1	1	1	1	1	1	1	1	1	1	1	1	1	1	1	1	1	1	1	1	1	1	1	1	1	1	1	1	1	1	1	1	1	1	1	1	1	1
SAL0000111	atpC	ATP synthase epsilon chain	1	1	1	1	1	1	1	1	1	1	1	1	1	1	1	1	1	1	1	1	1	1	1	1	1	1	1	1	1	1	1	1	1	1	1	1	1	1	1	1	1
SAL0000112	coaE	Dephospho-CoA kinase	1	1	1	1	1	1	1	1	1	1	1	1	1	1	1	1	1	1	1	1	1	1	1	1	1	1	1	1	1	1	1	1	1	1	1	1	1	1	1	1	2
SAL0000113	hp	hypothetical protein	1	1	1	1	1	1	1	1	1	1	1	1	1	1	1	1	1	1	1	1	1	1	1	1	1	1	1	1	1	1	1	1	1	1	1	1	1	1	1	1	1
SAL0000114	pheS	Phenylalanine--tRNA ligase alpha subunit	1	1	1	1	1	1	1	1	1	1	1	1	1	1	1	1	1	1	1	1	1	1	1	1	1	1	1	1	1	1	1	1	1	1	1	1	1	1	1	1	1
SAL0000115	hp	hypothetical protein	1	1	1	1	1	1	1	1	1	1	1	1	1	1	1	1	1	1	1	1	1	1	1	1	1	1	1	1	1	1	1	1	1	1	1	1	1	1	1	1	1
SAL0000116	hp	hypothetical protein	1	1	1	1	1	1	1	1	1	1	1	1	1	1	1	1	1	1	1	1	1	1	1	1	1	1	1	1	1	1	1	1	1	1	1	1	1	1	1	1	2
SAL0000117	glnQ_3	Glutamine transport ATP-binding protein GlnQ	1	1	1	1	1	1	1	3	1	1	1	1	1	1	1	1	1	1	1	1	1	1	1	1	1	1	1	1	1	1	1	1	1	1	1	1	1	1	1	1	2
SAL0000118	prsA	Foldase protein PrsA precursor	1	1	1	1	1	1	1	1	1	1	1	1	1	1	1	1	1	1	1	1	1	1	1	1	1	1	1	1	1	1	1	1	1	1	1	1	1	1	1	1	1
SAL0000119	dnaD	DNA replication protein DnaD	1	1	1	1	1	1	1	1	1	1	1	1	1	1	1	1	1	1	1	1	1	1	1	1	1	1	1	1	1	1	1	1	1	1	1	1	1	1	1	1	2
SAL0000120	hp	hypothetical protein	1	1	1	1	1	1	1	1	1	1	1	1	1	1	1	1	1	1	1	1	1	1	1	1	1	1	1	1	1	1	1	1	1	1	1	1	1	1	1	1	2

SAL0000121	atpH	ATP synthase subunit delta	1	1	1	1	1	1	1	1	1	1	1	1	1	1	1	1	1	1	1	1	1	1	1	1	1	1	1	1	1	1	1	1	1	1	1	1	1	1	1	1	1
SAL0000122	hp	hypothetical protein	1	1	1	1	1	1	1	1	1	1	1	1	1	1	1	1	1	1	1	1	1	1	1	1	1	1	1	1	1	1	1	1	1	1	1	1	1	1	1	1	2
SAL0000123	plsY_2	Glycerol-3-phosphate acyltransferase	1	1	1	1	1	2	1	1	1	1	1	1	1	1	1	1	1	1	1	1	1	1	1	1	1	1	1	1	1	1	1	1	1	1	1	1	1	1	1	1	3
SAL0000124	rpsT	30S ribosomal protein S20	1	1	1	1	1	1	1	1	1	1	1	1	1	1	1	1	1	1	1	1	1	1	1	1	1	1	1	1	1	1	1	1	1	1	1	1	1	1	1	1	1
SAL0000125	hp	hypothetical protein	1	1	1	1	1	1	1	1	1	1	1	1	1	1	1	1	1	1	1	1	1	1	1	1	1	1	1	1	1	1	1	1	1	1	1	1	1	1	1	1	1
SAL0000126	hp	hypothetical protein	1	1	1	1	1	1	1	1	1	1	1	1	1	1	1	1	1	1	1	1	1	1	1	1	1	1	1	1	1	1	1	1	1	1	1	1	1	1	1	1	1
SAL0000127	dnaX_2	DNA polymerase III subunit tau	1	1	1	1	1	1	1	1	1	1	1	1	1	1	1	1	1	1	1	1	1	1	1	1	1	1	1	1	1	1	1	1	1	1	1	1	1	1	1	1	1
SAL0000128	ccpA_2	Catabolite control protein A	1	1	1	1	1	1	1	1	1	1	1	3	1	1	1	1	1	1	1	1	1	1	1	1	1	1	1	1	1	1	1	1	1	1	1	1	1	1	1	1	2
SAL0000129	rlmCD_1	23S rRNA (uracil-C(5))-methyltransferase RlmCD	1	1	1	1	1	1	1	1	1	1	1	1	1	1	1	1	1	1	1	1	1	1	1	1	1	1	1	1	1	1	1	1	1	1	1	1	1	1	1	1	1
SAL0000130	mreB_2	Rod shape-determining protein MreB	1	1	1	1	1	1	1	1	1	1	1	1	1	1	1	1	1	1	1	1	1	1	1	1	1	1	1	1	1	1	1	1	1	1	1	1	1	1	1	1	1
SAL0000131	proC	Pyrroline-5-carboxylate reductase	1	1	1	1	1	1	1	1	1	1	1	1	1	1	1	1	1	1	1	1	1	1	1	1	1	1	1	1	1	1	1	1	1	1	1	1	1	1	1	1	1
SAL0000132	crr	Glucose-specific phosphotransferase enzyme IIA component	1	1	1	1	1	1	1	1	1	1	1	1	1	1	1	1	1	1	1	1	1	1	1	1	1	1	1	1	1	1	1	1	1	1	1	1	1	1	1	1	1
SAL0000133	group_1640	Aspartate racemase	1	1	1	1	1	1	1	1	1	1	1	1	1	1	1	1	1	1	1	1	1	1	1	1	1	1	1	1	1	1	1	1	1	1	1	1	1	1	1	1	2
SAL0000134	hp	hypothetical protein	1	1	2	1	1	1	1	2	1	1	1	1	1	1	2	1	1	1	1	1	1	1	1	1	1	1	1	2	1	2	1	2	1	1	1	1	1	1	1	1	3
SAL0000135	hp	hypothetical protein	1	1	1	1	1	1	1	1	1	1	1	1	1	1	1	1	1	1	1	1	1	1	1	1	1	1	2	1	1	1	1	1	1	1	1	1	1	1	1	1	1
SAL0000136	rsmG	Ribosomal RNA small subunit methyltransferase G	1	1	1	1	1	1	1	1	1	1	1	1	1	1	1	1	1	1	1	1	1	1	1	1	1	1	1	1	1	1	1	1	1	1	1	1	1	1	1	1	2
SAL0000137	hp	hypothetical protein	1	1	1	1	1	1	1	1	1	1	1	1	1	1	1	1	1	1	1	1	1	1	1	1	1	1	1	1	1	1	1	1	1	1	1	1	1	1	1	1	1
SAL0000138	groS	10 kDa chaperonin	1	1	1	1	1	1	1	1	1	1	1	1	1	1	1	1	1	1	1	1	1	1	1	1	1	1	1	1	1	1	1	1	1	1	1	1	1	1	1	1	2
SAL0000139	hp	hypothetical protein	1	1	1	1	1	1	1	1	1	1	1	1	1	1	1	1	1	1	1	1	1	1	1	1	1	1	1	1	1	1	1	1	1	1	1	1	1	1	1	1	1
SAL0000140	noc	Nucleoid occlusion protein	1	1	1	1	1	1	1	1	1	1	1	1	1	1	1	1	1	1	1	1	1	1	1	1	1	1	1	1	1	1	1	1	1	1	1	1	1	1	1	1	2
SAL0000141	manX_2	PTS system mannose-specific EIIAB component	1	1	1	1	1	1	1	1	1	1	1	1	1	1	1	1	1	1	1	1	1	1	1	1	1	1	1	1	1	1	1	1	1	1	1	1	1	1	1	1	2
SAL0000142	hp	hypothetical protein	1	1	1	1	1	1	1	1	1	1	1	1	1	1	1	1	1	1	1	1	1	1	1	1	1	1	1	1	1	1	1	1	1	1	1	1	1	1	1	1	1
SAL0000143	hp	hypothetical protein	1	1	1	1	1	1	1	1	1	1	1	2	1	1	1	1	1	1	1	1	1	1	1	1	1	1	1	1	1	1	1	1	1	1	1	1	1	1	1	1	3
SAL0000144	mecA	Adapter protein MecA	1	1	1	1	1	1	1	1	1	1	1	1	1	1	1	1	1	1	1	1	1	1	1	1	1	1	1	1	1	1	1	1	1	1	1	1	1	1	1	1	2
SAL0000145	rplX	50S ribosomal protein L24	1	1	1	1	1	1	1	1	1	1	1	1	1	1	1	1	1	1	1	1	1	1	1	1	1	1	1	1	1	1	1	1	1	1	1	1	1	1	1	1	1
SAL0000146	pheT_2	Phenylalanine--tRNA ligase beta subunit	1	1	1	1	1	1	1	1	1	1	1	1	1	1	1	1	1	1	1	1	1	1	1	1	1	1	1	1	1	1	1	1	1	1	1	1	1	1	1	1	1
SAL0000147	hp	hypothetical protein	1	1	1	1	1	1	1	1	1	1	1	1	1	1	1	1	1	1	1	1	1	1	1	1	1	1	1	1	1	1	1	1	1	1	1	1	1	1	1	1	1
SAL0000148	rplF	50S ribosomal protein L6	1	1	1	1	1	1	1	1	1	1	1	1	1	1	1	1	1	1	1	1	1	1	1	1	1	1	1	1	1	1	1	1	1	1	1	1	1	1	1	1	1
SAL0000149	hp	hypothetical protein	1	1	1	1	1	1	1	1	1	1	1	1	1	1	1	1	1	1	1	1	1	1	1	1	1	1	1	1	1	1	1	1	1	1	1	1	1	1	1	1	1
SAL0000150	pgcA	Phosphoglucomutase	1	1	1	1	1	1	1	2	1	1	1	1	1	1	1	1	1	1	1	1	1	1	1	1	1	1	1	1	1	1	1	1	1	1	1	1	1	1	1	1	3
SAL0000151	group_1657	L-2-hydroxyisocaproate dehydrogenase	1	1	1	1	1	1	1	1	1	1	1	1	1	1	1	1	1	1	1	1	1	1	1	1	1	1	1	1	1	1	1	1	1	1	1	1	1	1	1	1	2
SAL0000152	argS	Arginine--tRNA ligase	1	1	1	1	1	1	1	1	1	1	1	1	1	1	1	1	1	1	1	1	1	1	1	1	1	1	1	1	1	1	1	1	1	1	1	1	1	1	1	1	1
SAL0000153	hp	hypothetical protein	1	1	1	1	1	1	1	1	1	1	1	1	1	1	1	1	1	1	1	1	1	1	1	1	1	1	1	1	1	1	1	1	1	1	1	1	1	1	1	1	1
SAL0000154	thrS	Threonine--tRNA ligase	1	1	1	1	1	1	1	1	1	1	1	1	1	1	1	1	1	1	1	1	1	1	1	1	1	1	1	1	1	1	1	1	1	1	1	1	1	1	1	1	2
SAL0000155	hp	hypothetical protein	1	1	1	1	1	1	1	1	1	1	1	1	1	1	1	1	1	1	1	1	1	1	1	1	1	1	1	1	1	1	1	1	1	1	1	1	1	1	1	1	1
SAL0000156	group_1662	preprotein translocase subunit YajC	1	1	1	1	1	1	1	1	1	1	1	1	1	1	1	1	1	1	1	1	1	1	1	1	1	1	1	1	1	1	1	1	1	1	1	1	1	1	1	1	1
SAL0000157	hp	hypothetical protein	1	1	1	1	1	1	1	2	1	1	1	1	1	1	1	1	1	1	1	1	1	1	1	1	1	1	1	1	1	1	1	1	1	1	1	1	1	1	1	1	1
SAL0000158	hp	hypothetical protein	1	1	1	1	1	1	1	1	1	1	1	1	1	1	1	1	1	1	1	1	1	1	1	1	1	1	1	1	1	1	1	1	1	1	1	1	1	1	1	1	1
SAL0000159	catM	HTH-type transcriptional regulator CatM	1	1	1	1	1	1	1	1	1	1	1	1	1	1	1	1	1	1	1	1	1	1	1	1	1	1	1	1	1	1	1	1	1	1	1	1	1	1	1	1	2
SAL0000160	rpsB	30S ribosomal protein S2	1	1	1	1	1	1	1	1	1	1	1	1	1	1	1	1	1	1	1	1	1	1	1	1	1	1	1	1	1	1	1	1	1	1	1	1	1	1	1	1	1
SAL0000161	whiA	Putative sporulation transcription regulator WhiA	1	1	1	1	1	1	1	1	1	1	1	1	1	1	1	1	1	1	1	1	1	1	1	1	1	1	1	1	1	1	1	1	1	1	1	1	1	1	1	1	2
SAL0000162	hp	hypothetical protein	1	1	1	1	1	1	1	1	1	1	1	1	1	1	1	1	1	1	1	1	1	1	1	1	1	1	1	1	1	1	1	1	1	1	1	1	1	1	1	1	1
SAL0000163	gpmA_3	2,3-bisphosphoglycerate-dependent phosphoglycerate mutase	1	1	1	1	1	1	1	1	1	1	1	1	1	1	1	1	1	1	1	1	1	1	1	1	1	1	1	1	1	1	1	1	1	1	1	1	1	1	1	1	2
SAL0000164	hp	hypothetical protein	1	1	1	1	1	1	1	1	1	1	1	1	1	1	1	1	1	1	1	1	1	1	1	1	1	1	1	1	1	1	1	1	1	1	1	1	1	1	1	1	1
SAL0000165	hp	hypothetical protein	1	1	1	1	1	1	1	1	1	1	1	1	1	1	1	1	1	1	1	1	1	1	1	1	1	1	1	1	1	1	1	1	1	1	1	1	1	1	1	1	1
SAL0000166	hp	hypothetical protein	1	1	1	1	1	1	1	1	1	1	1	1	1	1	1	1	1	1	1	1	1	1	1	1	1	1	1	1	1	1	1	1	1	1	1	1	1	1	1	1	1
SAL0000167	citE	Citrate lyase subunit beta	1	1	1	1	1	1	1	1	1	1	1	1	1	1	1	1	1	1	1	1	1	1	1	1	1	1	1	1	1	1	1	1	1	1	1	1	1	1	1	1	1
SAL0000168	hp	hypothetical protein	1	1	1	1	1	1	1	1	1	1	1	1	1	1	1	1	1	1	1	1	1	1	1	1	1	1	1	1	1	1	1	1	1	1	1	1	1	1	1	1	1
SAL0000169	znuC	Zinc import ATP-binding protein ZnuC	1	1	1	1	1	1	1	1	1	1	1	1	1	1	2	1	1	1	1	1	1	1	1	1	1	1	1	1	1	1	1	1	1	1	1	1	1	1	1	1	3
SAL0000170	trpS2	Tryptophan--tRNA ligase 2	1	1	1	1	1	1	1	1	1	1	1	1	1	1	1	1	1	1	1	1	1	1	1	1	1	1	1	1	1	1	1	1	1	1	1	1	1	1	1	1	1
SAL0000171	glpK_2	Glycerol kinase	1	1	1	1	1	1	1	1	1	1	1	1	1	1	1	1	1	1	1	1	1	1	1	1	1	1	1	1	1	1	1	1	1	1	1	1	1	1	1	1	1
SAL0000172	hp	hypothetical protein	1	1	1	1	1	1	1	1	1	1	1	1	1	1	1	1	1	1	1	1	1	1	1	1	1	1	1	1	1	1	1	1	1	1	1	1	1	1	1	1	1
SAL0000173	oppC_2	Oligopeptide transport system permease protein OppC	1	1	1	1	1	1	1	1	1	1	1	1	1	1	1	1	1	1	1	1	1	1	1	1	1	1	1	1	1	1	1	1	1	1	1	1	1	1	1	1	1
SAL0000174	hp	hypothetical protein	1	1	1	1	1	1	1	1	1	1	1	1	1	1	1	1	1	1	1	1	1	1	1	1	1	1	1	1	1	1	1	1	1	1	1	1	1	1	1	1	1
SAL0000175	group_1681	Glycosyltransferase sugar-binding region containing DXD motif protein	1	1	1	1	1	1	1	1	1	1	1	1	1	1	1	1	1	1	1	1	1	1	1	1	1	1	1	1	1	1	1	1	1	1	1	1	1	1	1	1	2
SAL0000176	group_1682	putative oxidoreductase/MSMEI_2347	1	1	1	1	1	1	1	1	1	1	1	1	1	1	1	1	1	1	1	1	1	1	1	1	1	1	1	1	1	1	1	1	1	1	1	1	1	1	1	1	2
SAL0000177	gpsA	Glycerol-3-phosphate dehydrogenase [NAD(P)]	1	1	1	1	1	1	1	1	1	1	1	1	1	1	1	1	1	1	1	1	1	1	1	1	1	1	1	1	1	1	1	1	1	1	1	1	1	1	1	1	2
SAL0000178	msrA	Peptide methionine sulfoxide reductase MsrA	1	1	1	1	1	1	1	1	1	1	1	1	1	1	1	1	1	1	1	1	1	1	1	1	1	1	1	1	1	1	1	1	1	1	1	1	1	1	1	1	1
SAL0000179	coaD	Phosphopantetheine adenylyltransferase	1	1	1	1	1	1	1	1	1	1	1	2	1	1	1	1	1	1	1	1	1	1	1	1	1	1	1	1	1	1	1	1	1	1	1	1	1	1	1	1	1
SAL0000180	rpsM	30S ribosomal protein S13	1	1	1	1	1	1	1	1	1	1	1	1	1	1	1	1	1	1	1	1	1	1	1	1	1	1	1	1	1	1	1	1	1	1	1	1	1	1	1	1	1
SAL0000181	ilvE	Branched-chain-amino-acid aminotransferase	1	1	1	1	1	1	1	1	1	1	1	1	1	1	1	1	1	1	1	1	1	1	1	1	1	1	1	1	1	1	1	1	1	1	1	1	1	1	1	1	1
SAL0000182	pksG	Polyketide biosynthesis 3-hydroxy-3-methylglutaryl-ACP synthase PksG	1	1	1	1	1	1	1	1	1	1	1	1	1	1	1	1	1	1	1	1	1	1	1	1	1	1	1	1	1	1	1	1	1	1	1	1	1	1	1	1	2
SAL0000183	soj	Sporulation initiation inhibitor protein Soj	1	1	1	1	1	1	1	1	1	1	1	1	1	1	1	1	1	1	1	1	1	1	1	1	1	1	1	1	1	1	1	1	1	1	1	1	1	1	1	1	2
SAL0000184	rsmC	Ribosomal RNA small subunit methyltransferase C	1	1	3	1	1	2	1	1	1	1	1	4	1	1	1	1	1	1	1	1	1	1	1	1	1	1	1	1	1	1	1	1	1	1	1	1	1	1	1	1	1

SAL0000185	pepDA_1	Dipeptidase A	1	1	1	1	1	1	1	1	1	1	1	1	1	1	1	1	1	1	1	1	1	1	1	1	1	1	1	1	1	1	1	1	1	1	1	1	1	1	1	1	1
SAL0000186	ywaC	GTP pyrophosphokinase YwaC	1	1	1	1	1	1	1	1	1	1	1	1	1	1	1	1	1	1	1	1	1	1	1	1	1	1	1	1	1	1	1	1	1	1	1	1	1	1	1	1	1
SAL0000187	group_1692	Ribonuclease J 2	1	1	2	1	1	1	1	1	1	1	1	1	1	1	1	1	1	1	1	1	1	1	1	1	1	1	1	1	1	1	1	1	1	1	1	1	1	1	1	1	1
SAL0000188	hp	hypothetical protein	1	1	1	1	1	1	1	1	1	1	1	1	1	1	1	1	1	1	1	1	1	1	1	1	1	1	1	1	1	1	1	1	1	1	1	1	1	1	1	1	1
SAL0000189	citD	Citrate lyase acyl carrier protein	1	1	1	1	1	1	1	2	1	1	1	1	1	1	1	1	1	1	1	1	1	1	1	1	1	1	1	1	1	1	1	1	1	1	1	1	1	1	1	1	1
SAL0000190	oppD_2	Oligopeptide transport ATP-binding protein OppD	1	1	1	1	1	1	1	1	1	1	1	1	1	1	1	1	1	1	1	1	1	1	1	1	1	1	1	1	1	1	1	1	1	1	1	1	1	1	1	1	1
SAL0000191	group_1696	phosphoribosylformylglycinamidine synthase subunit PurS	1	1	1	1	1	1	1	1	1	1	1	1	1	1	1	1	1	1	1	1	1	1	1	1	1	1	1	1	1	1	1	1	1	1	1	1	1	1	1	1	1
SAL0000192	scpA	Segregation and condensation protein A	1	1	1	1	1	1	1	1	1	1	1	1	1	1	1	1	1	1	1	1	1	1	1	1	1	1	1	1	1	1	1	1	1	1	1	1	1	1	1	1	2
SAL0000193	hp	hypothetical protein	1	1	1	1	1	1	1	1	1	1	1	1	1	1	1	1	1	1	1	1	1	1	1	1	1	1	1	1	1	1	1	1	1	1	1	1	1	1	1	1	1
SAL0000194	hp	hypothetical protein	1	1	1	1	1	1	1	1	1	1	1	1	1	1	1	1	1	1	1	1	1	1	1	1	1	1	1	1	1	1	1	1	1	1	1	1	1	1	1	1	1
SAL0000195	rpsJ	30S ribosomal protein S10	1	1	1	1	1	1	1	1	1	1	1	1	1	1	1	1	1	1	1	1	1	1	1	1	1	1	1	1	1	1	1	1	1	1	1	1	1	1	1	1	1
SAL0000196	recR	Recombination protein RecR	1	1	1	1	1	1	1	1	1	1	1	1	1	1	1	1	1	1	1	1	1	1	1	1	1	1	1	1	1	1	1	1	1	1	1	1	1	1	1	1	1
SAL0000197	hp	hypothetical protein	1	1	1	1	1	1	1	1	1	1	1	1	1	1	1	1	1	1	1	1	1	1	1	1	1	1	1	1	1	1	1	1	1	1	1	1	1	1	1	1	1
SAL0000198	group_1703	Putative membrane protein insertion efficiency factor	1	1	1	1	1	1	1	1	1	1	1	1	1	1	1	1	1	1	1	1	1	1	1	1	1	1	1	1	1	1	1	1	1	1	1	1	1	1	1	1	2
SAL0000199	hp	hypothetical protein	1	1	1	1	1	1	1	2	1	1	1	1	1	1	1	1	1	1	1	1	1	1	1	1	1	1	1	1	1	1	1	1	1	1	1	1	1	1	1	1	1
SAL0000200	hp	hypothetical protein	1	1	1	1	1	1	1	1	1	1	1	1	1	1	1	1	1	1	1	1	1	1	1	1	1	1	1	1	1	1	1	1	1	1	1	1	1	1	1	1	2
SAL0000201	group_1706	putative HTH-type transcriptional regulator	1	1	1	1	1	1	1	1	1	1	1	1	1	1	1	1	1	1	1	1	1	1	1	1	1	1	1	1	1	1	1	1	1	1	1	1	1	1	1	1	1
SAL0000202	mutT	8-oxo-dGTP diphosphatase	1	1	1	1	1	1	1	1	1	1	1	1	1	1	1	1	1	1	1	1	1	1	1	1	1	1	1	1	1	1	1	1	1	1	1	1	1	1	1	1	1
SAL0000203	ribD	Riboflavin biosynthesis protein ribD	1	1	1	1	1	1	1	1	1	2	1	1	1	1	1	1	1	1	1	1	1	1	1	1	1	1	1	1	1	1	1	1	1	1	1	1	1	1	1	1	1
SAL0000204	group_1709	Membrane transport protein	1	1	1	2	1	1	1	1	1	1	1	1	1	1	1	1	1	1	1	1	1	1	1	1	1	1	2	1	1	1	1	1	1	1	1	1	1	1	1	1	1
SAL0000205	pepDA_2	Dipeptidase A	1	1	2	2	1	2	2	2	1	2	1	2	1	1	2	1	1	1	1	1	1	1	1	1	1	1	3	2	2	2	1	2	4	1	1	1	1	1	1	1	5
SAL0000206	map	Methionine aminopeptidase 1	1	1	1	1	1	1	1	1	1	1	1	1	1	1	1	1	1	1	1	1	1	1	1	1	1	1	1	1	1	1	1	1	1	1	1	1	1	1	1	1	2
SAL0000207	rpsI	30S ribosomal protein S9	1	1	1	1	1	1	1	1	1	1	1	2	1	1	1	1	1	1	1	1	1	1	1	1	1	1	1	1	1	1	1	1	1	1	1	1	1	1	1	1	1
SAL0000208	hp	hypothetical protein	1	1	1	1	1	1	1	1	1	1	1	1	1	1	1	1	1	1	1	1	1	1	1	1	1	1	1	1	1	1	1	1	1	1	1	1	1	1	1	1	1
SAL0000209	hp	hypothetical protein	1	1	1	1	1	1	1	1	1	1	1	1	1	1	1	1	1	1	1	1	1	1	1	1	1	1	1	1	1	1	1	1	1	1	1	1	1	1	1	1	2
SAL0000210	hp	hypothetical protein	1	1	1	1	1	1	1	1	1	1	1	1	1	1	1	1	1	1	1	1	1	1	1	1	1	1	1	1	1	1	1	1	1	1	1	1	1	1	1	1	1
SAL0000211	group_1717	Acid shock protein	1	1	1	1	1	1	1	1	1	1	1	1	1	1	1	1	1	1	1	1	1	1	1	1	1	1	1	1	1	1	1	1	1	1	1	1	1	1	1	1	1
SAL0000212	group_1718	Putative peptidoglycan binding domain protein	1	1	1	1	1	1	1	1	1	1	1	1	1	1	1	1	1	1	1	1	1	1	1	1	1	1	1	1	1	1	1	1	1	1	1	1	1	1	1	1	2
SAL0000213	hp	hypothetical protein	1	1	1	1	1	1	1	1	1	1	1	1	1	1	1	1	1	1	1	1	1	1	1	1	1	1	1	1	1	1	1	1	1	1	1	1	1	1	1	1	2
SAL0000214	hp	hypothetical protein	1	1	1	1	1	1	1	1	1	1	1	1	1	1	1	1	1	1	1	1	1	1	1	1	1	1	1	1	1	1	1	1	1	1	1	1	1	1	1	1	1
SAL0000215	tagD	Glycerol-3-phosphate cytidylyltransferase	1	1	1	1	1	1	1	1	1	1	1	1	1	1	1	1	1	1	1	1	1	1	1	1	1	1	1	1	1	1	1	1	1	1	1	1	1	1	1	1	2
SAL0000216	tdk	Thymidine kinase	1	1	1	2	1	1	1	1	1	1	1	1	1	1	1	1	1	1	1	1	1	1	1	1	1	1	2	1	1	1	1	1	1	1	1	1	1	1	1	1	1
SAL0000217	group_1723	PTS system glucitol/sorbitol-specific transporter subunit IIA	1	1	1	1	1	1	1	1	1	1	1	1	1	1	1	1	1	1	1	1	1	1	1	1	1	1	1	1	1	1	1	1	1	1	1	1	1	1	1	1	2
SAL0000218	rpoZ	DNA-directed RNA polymerase subunit omega	1	1	1	1	1	1	1	1	1	1	1	1	1	1	1	1	1	1	1	1	1	1	1	1	1	1	1	1	1	1	1	1	1	1	1	1	1	1	1	1	2
SAL0000219	greA_1	Transcription elongation factor GreA	1	1	1	1	1	1	1	1	1	1	1	1	1	1	1	1	1	1	1	1	1	1	1	1	1	1	1	1	1	1	1	1	1	1	1	1	1	1	1	1	1
SAL0000220	glgD	Glycogen biosynthesis protein GlgD	1	1	1	1	1	1	1	1	1	1	1	1	1	1	1	1	1	1	1	1	1	1	1	1	1	1	1	1	1	1	1	1	1	1	1	1	1	1	1	1	2
SAL0000221	hp	hypothetical protein	1	1	1	1	1	1	1	1	1	1	1	1	1	1	1	1	1	1	1	1	1	1	1	1	1	1	1	1	1	1	1	1	1	1	1	1	1	1	1	1	2
SAL0000222	group_1730	acyl-CoA esterase	1	1	1	1	1	1	1	1	1	1	1	1	1	1	1	1	1	1	1	1	1	1	1	1	1	1	1	1	1	1	1	1	1	1	1	1	1	1	1	1	2
SAL0000223	ccpA_3	Catabolite control protein A	1	1	1	1	1	1	1	1	1	1	1	1	1	1	1	1	1	1	1	1	1	1	1	1	1	1	1	1	1	1	1	1	1	1	1	1	1	1	1	1	2
SAL0000224	rpsP	30S ribosomal protein S16	1	1	1	1	1	1	1	1	1	1	1	1	1	1	1	1	1	1	1	1	1	1	1	1	1	1	1	1	1	1	1	1	1	1	1	1	1	1	1	1	2
SAL0000225	hp	hypothetical protein	1	1	1	1	1	1	1	1	1	1	1	1	1	1	1	1	1	1	1	1	1	1	1	1	1	1	1	1	1	1	1	1	1	1	1	1	1	1	2	1	1
SAL0000226	hp	hypothetical protein	1	1	1	1	1	1	1	1	1	1	1	1	1	1	1	1	1	1	1	1	1	1	1	1	1	1	1	1	1	1	1	1	1	1	1	1	1	1	1	1	1
SAL0000227	metK	S-adenosylmethionine synthase	1	1	1	1	1	1	1	1	1	1	1	1	1	1	1	1	1	1	1	1	1	1	1	1	1	1	1	1	1	1	1	1	1	1	1	1	1	1	1	1	1
SAL0000228	luxS	S-ribosylhomocysteine lyase	1	1	1	1	1	1	1	1	1	1	1	1	1	1	1	1	1	1	1	1	1	1	1	1	1	1	1	1	1	1	1	1	1	1	1	1	1	1	1	1	2
SAL0000229	ywlC	Threonylcarbamoyl-AMP synthase	1	1	1	1	1	1	1	1	1	1	1	1	1	1	1	1	1	1	1	1	1	1	1	1	1	1	1	1	1	1	1	1	1	1	1	1	1	1	1	1	1
SAL0000230	copZ	Copper chaperone CopZ	1	1	1	1	1	1	1	1	1	1	1	1	1	1	1	1	1	1	1	1	1	1	1	1	1	1	1	1	1	1	1	1	1	1	1	1	1	1	1	1	1
SAL0000231	hp	hypothetical protein	1	1	1	1	1	1	1	1	1	1	1	1	1	1	1	1	1	1	1	1	1	1	1	1	1	1	1	1	1	1	1	1	1	1	1	1	1	1	1	1	1
SAL0000232	hp	hypothetical protein	5	5	3	5	5	5	5	3	5	5	5	5	5	5	3	5	5	5	5	5	5	5	5	5	5	5	5	3	5	3	5	3	5	5	5	5	5	5	5	5	4
SAL0000233	hp	hypothetical protein	1	1	1	1	1	1	1	1	1	1	1	1	1	1	1	1	1	1	1	1	1	1	1	1	1	1	1	1	1	1	1	1	1	1	1	1	1	1	1	1	1
SAL0000234	polC_1	DNA polymerase III PolC-type	1	1	1	1	1	1	1	1	1	1	1	1	1	1	1	1	1	1	1	1	1	1	1	1	1	1	1	1	1	1	1	1	1	1	1	1	1	1	1	1	1
SAL0000235	hp	hypothetical protein	1	1	1	1	1	1	1	1	1	1	1	1	1	1	1	1	1	1	1	1	1	1	1	1	1	1	1	1	1	1	1	1	1	1	1	1	1	1	1	1	1
SAL0000236	hp	hypothetical protein	1	1	1	1	1	1	1	1	1	1	1	1	1	1	1	1	1	1	1	1	1	1	1	1	1	1	1	1	1	1	1	1	1	1	1	1	1	1	1	1	1
SAL0000237	mrnC	Mini-ribonuclease 3	1	1	1	1	1	1	1	1	1	1	1	1	1	1	1	1	1	1	1	1	1	1	1	1	1	1	1	1	1	1	1	1	1	1	1	1	1	1	1	1	1
SAL0000238	rplE	50S ribosomal protein L5	1	1	1	1	1	1	1	1	1	1	1	1	1	1	1	1	1	1	1	1	1	1	1	1	1	1	1	1	1	1	1	1	1	1	1	1	1	1	1	1	1
SAL0000239	btr_2	HTH-type transcriptional activator Btr	1	1	1	1	1	1	1	1	1	1	1	1	1	1	1	1	1	1	1	1	1	1	1	1	1	1	1	1	1	1	1	1	1	1	1	1	1	1	1	1	1
SAL0000240	hp	hypothetical protein	1	1	1	1	1	1	1	1	1	1	1	1	1	1	1	1	1	1	1	1	1	1	1	1	1	1	1	1	1	1	1	1	1	1	1	1	1	1	1	1	1
SAL0000241	group_1748	FMN-binding domain protein	1	1	1	1	1	1	1	1	1	1	1	1	1	1	1	1	1	1	1	1	1	1	1	1	1	1	1	1	1	1	1	1	1	1	1	1	1	1	1	1	1
SAL0000242	mreC	Cell shape-determining protein MreC precursor	1	1	1	1	1	1	1	1	1	1	1	1	1	1	1	1	1	1	1	1	1	1	1	1	1	1	1	1	1	1	1	1	1	1	1	1	1	1	1	1	1
SAL0000243	spxA_2	Regulatory protein Spx	1	1	1	1	1	1	1	1	1	1	1	1	1	1	1	1	1	1	1	1	1	1	1	1	1	1	1	1	1	1	1	1	1	1	1	1	1	1	1	1	1
SAL0000244	hp	hypothetical protein	1	1	1	1	1	1	1	1	1	1	1	1	1	1	1	1	1	1	1	1	1	1	1	1	1	1	1	1	1	1	1	1	1	1	1	1	1	1	1	1	1
SAL0000245	hp	hypothetical protein	1	1	1	1	1	1	1	1	1	1	1	1	1	1	1	1	1	1	1	1	1	1	1	1	1	1	1	1	1	1	1	1	1	1	1	1	1	1	1	1	2
SAL0000246	ycaM_1	Inner membrane transporter YcaM	1	1	1	1	1	1	1	1	1	1	1	1	1	1	1	1	1	1	1	1	1	1	1	1	1	1	1	1	1	1	1	1	1	1	1	1	1	1	1	1	1
SAL0000247	hp	hypothetical protein	1	1	1	1	1	1	1	1	1	1	1	1	1	1	1	1	1	1	1	1	1	1	1	1	1	1	1	1	1	1	1	1	1	1	1	1	1	1	1	1	1
SAL0000248	oppB	Oligopeptide transport system permease protein OppB	1	1	1	2	1	1	1	1	1	1	1	1	1	1	1	1	1	1	1	1	1	1	1	1	1	1	2	1	1	1	1	1	1	1	1	1	1	1	1	1	1

SAL0000249	apt	Adenine phosphoribosyltransferase	1	1	1	2	1	1	1	1	1	1	1	1	1	1	1	1	1	1	1	1	1	1	1	1	1	1	2	1	1	1	1	1	1	1	1	1	1	1	1	1	3
SAL0000250	rplM	50S ribosomal protein L13	1	1	2	1	1	1	1	1	1	1	1	1	1	1	2	1	1	1	1	1	1	1	1	1	1	1	1	1	1	2	1	2	1	1	1	1	1	1	1	1	1
SAL0000251	hp	hypothetical protein	1	1	1	1	1	1	1	1	1	1	1	1	1	1	1	1	1	1	1	1	1	1	1	1	1	1	1	1	1	1	1	1	1	1	1	1	1	1	1	1	1
SAL0000252	dhaM	PTS-dependent dihydroxyacetone kinase, phosphotransferase subunit DhaM	1	1	1	1	1	1	1	1	1	1	1	1	1	1	1	1	1	1	1	1	1	1	1	1	1	1	1	1	1	1	1	1	1	1	1	1	1	1	1	1	1
SAL0000253	group_1760	putative transcriptional regulatory protein	1	1	1	1	1	1	1	1	1	1	1	1	1	1	1	1	1	1	1	1	1	1	1	1	1	1	1	1	1	1	1	1	1	1	1	1	1	1	1	1	1
SAL0000254	ftsL_2	Cell division protein FtsL	1	1	1	1	1	1	1	1	1	1	1	1	1	1	1	1	1	1	1	1	1	1	1	1	1	1	1	1	1	1	1	1	1	1	1	1	1	1	1	1	1
SAL0000255	yidA_3	Sugar phosphatase YidA	1	1	1	1	1	1	1	1	1	1	1	1	1	1	1	1	1	1	1	1	1	1	1	1	1	1	1	1	1	1	1	1	1	1	1	1	1	1	1	1	1
SAL0000256	mazF9	mRNA interferase MazF9	1	1	1	1	1	1	1	1	1	1	1	1	1	1	1	1	1	1	1	1	1	1	1	1	1	1	1	1	1	1	1	1	1	1	1	1	1	1	1	1	1
SAL0000257	rarA_1	Replication-associated recombination protein A	1	1	1	1	1	1	1	1	1	1	1	1	1	1	1	1	1	1	1	1	1	1	1	1	1	1	1	1	1	1	1	1	1	1	1	1	1	1	1	1	2
SAL0000258	hp	hypothetical protein	1	1	1	1	1	1	1	1	1	1	1	1	1	1	1	1	1	1	1	1	1	1	1	1	1	1	1	1	1	1	1	1	1	1	1	1	1	1	1	1	2
SAL0000259	murI	Glutamate racemase	1	1	1	1	1	1	1	1	1	1	1	1	1	1	1	1	1	1	1	1	1	1	1	1	1	1	1	1	1	1	1	1	1	1	1	1	1	1	1	1	1
SAL0000260	ppaC	putative manganese-dependent inorganic pyrophosphatase	1	1	1	1	1	1	1	1	1	1	1	1	1	1	1	1	1	1	1	1	1	1	1	1	1	1	1	1	1	1	1	1	1	1	1	1	1	1	1	1	1
SAL0000261	group_1768	3-methyladenine DNA glycosylase	1	1	1	1	1	1	1	1	1	1	1	1	1	1	1	1	1	1	1	1	1	1	1	1	1	1	1	1	1	1	1	1	1	1	1	1	1	1	1	1	2
SAL0000262	hp	hypothetical protein	1	1	1	1	1	1	1	1	1	1	1	1	1	1	1	1	1	1	1	1	1	1	1	1	1	1	1	1	1	1	1	1	1	1	1	1	1	1	1	1	1
SAL0000263	group_1770	DSBA-like thioredoxin domain protein	1	1	1	1	1	1	1	1	1	1	1	1	1	1	1	1	1	1	1	1	1	1	1	1	1	1	1	1	1	1	1	1	1	1	1	1	1	1	1	1	1
SAL0000264	group_1771	preprotein translocase subunit SecG	1	1	1	1	1	1	1	1	1	1	1	1	1	1	1	1	1	1	1	1	1	1	1	1	1	1	1	1	1	1	1	1	1	1	1	1	1	1	1	1	1
SAL0000265	hp	hypothetical protein	1	1	1	1	1	1	1	1	1	1	1	1	1	1	1	1	1	1	1	1	1	1	1	1	1	1	1	1	1	1	1	1	1	1	1	1	1	1	1	1	2
SAL0000266	rpmC	50S ribosomal protein L29	1	1	1	1	1	1	1	2	1	1	1	1	1	1	1	1	1	1	1	1	1	1	1	1	1	1	1	1	1	1	1	1	1	1	1	1	1	1	1	1	1
SAL0000267	ruvA	Holliday junction ATP-dependent DNA helicase RuvA	1	1	1	1	1	1	1	1	1	1	1	1	1	1	1	1	1	1	1	1	1	1	1	1	1	1	1	1	1	1	1	1	1	1	1	1	1	1	1	1	1
SAL0000268	paiA	Protease synthase and sporulation negative regulatory protein PAI 1	1	1	1	1	1	1	1	1	1	1	1	1	1	1	1	1	1	1	1	1	1	1	1	1	1	1	1	1	1	1	1	1	1	1	1	1	1	1	1	1	2
SAL0000269	pspB_2	Putative phosphoserine phosphatase 2	1	1	1	1	1	1	1	1	1	1	1	1	1	1	1	1	1	1	1	1	1	1	1	1	1	1	1	1	1	1	1	1	1	1	1	1	1	1	1	1	1
SAL0000270	hp	hypothetical protein	1	1	1	1	1	1	1	1	1	1	1	1	1	1	1	1	1	1	1	1	1	1	1	1	1	1	1	1	1	1	1	1	1	1	1	1	1	1	1	1	2
SAL0000271	glnP_3	putative glutamine ABC transporter permease protein GlnP	1	1	1	1	1	1	1	1	1	1	1	1	1	1	1	1	1	1	1	1	1	1	1	1	1	1	1	1	1	1	1	1	1	1	1	1	1	1	1	1	1
SAL0000272	group_1779	putative type I restriction enzymeP M protein	1	1	1	1	1	1	1	1	1	1	1	1	1	1	1	1	1	1	1	1	1	1	1	1	1	1	1	1	2	1	1	1	1	1	1	1	1	1	1	1	3
SAL0000273	group_178	major facilitator superfamily transporter	1	1	1	1	1	1	1	2	1	1	1	1	1	1	1	1	1	1	1	1	1	1	3	1	1	1	1	1	1	1	1	1	1	1	1	1	1	1	1	1	1
SAL0000274	purC	Phosphoribosylaminoimidazole-succinocarboxamide synthase	1	1	1	1	1	1	1	1	1	1	1	1	1	1	1	1	1	1	1	1	1	1	1	1	1	1	1	1	1	1	1	1	1	1	1	1	1	1	1	1	1
SAL0000275	yycF_1	Transcriptional regulatory protein YycF	1	1	1	1	1	1	1	1	1	1	1	1	1	1	1	1	1	1	1	1	1	1	1	1	1	1	1	1	1	1	1	1	1	1	1	1	1	1	1	1	1
SAL0000276	purL	Phosphoribosylformylglycinamidine synthase 2	1	1	1	1	1	1	1	1	1	1	1	1	1	1	1	1	1	1	1	1	1	1	1	1	1	1	1	1	2	1	1	1	1	1	1	1	1	1	1	1	1
SAL0000277	lon	Lon protease	1	1	1	1	1	1	1	1	1	1	1	1	1	1	1	1	1	1	1	1	1	1	1	1	1	1	1	1	1	1	1	1	1	1	1	1	1	1	1	1	1
SAL0000278	tpiA	Triosephosphate isomerase	1	1	1	1	1	1	1	1	1	1	1	1	1	1	1	1	1	1	1	1	1	1	1	1	1	1	1	1	1	1	1	1	1	1	1	1	1	1	1	1	1
SAL0000279	lacC	Tagatose-6-phosphate kinase	1	1	1	1	1	1	1	1	1	1	1	1	1	1	1	1	1	1	1	1	1	1	1	1	1	1	1	1	1	1	1	1	1	1	1	1	1	1	1	1	1
SAL0000280	group_1786	putative acetyltransferase	1	1	1	1	1	1	1	1	1	1	1	1	1	1	1	1	1	1	1	1	1	1	1	1	1	1	1	1	1	1	1	1	1	1	1	1	1	1	1	1	1
SAL0000281	hp	hypothetical protein	1	1	1	1	1	1	1	1	1	1	1	1	1	1	1	1	1	1	1	1	1	1	1	1	1	1	1	1	1	1	1	1	1	1	1	1	1	1	1	1	1
SAL0000282	hp	hypothetical protein	1	1	1	1	1	1	1	1	1	1	1	1	1	1	1	1	1	1	1	1	1	1	1	1	1	1	1	1	1	1	1	1	1	1	1	1	1	1	1	1	2
SAL0000283	artQ_1	Arginine transport system permease protein ArtQ	1	1	1	1	1	1	1	1	1	1	1	1	1	1	3	1	1	1	1	1	1	1	1	1	1	1	1	1	1	1	1	1	1	1	1	1	1	1	1	1	2
SAL0000284	group_1790	preprotein translocase subunit SecE	1	1	1	1	1	1	1	1	1	1	1	1	1	1	1	1	1	1	1	1	1	1	1	1	1	1	1	1	1	1	1	1	1	1	1	1	1	1	1	1	1
SAL0000285	znuB	High-affinity zinc uptake system membrane protein ZnuB	1	1	1	1	1	1	1	1	1	1	1	1	1	1	1	1	1	1	1	1	1	1	1	1	1	1	1	1	1	1	1	1	1	1	1	1	1	1	1	1	2
SAL0000286	rpmA	50S ribosomal protein L27	1	1	1	1	1	1	1	1	1	1	1	1	1	1	1	1	1	1	1	1	1	1	1	1	1	1	1	1	1	1	1	1	1	1	1	1	1	1	1	1	1
SAL0000287	group_1793	pyrroline-5-carboxylate reductase	1	1	1	1	1	1	1	1	1	1	1	1	1	1	1	1	1	1	1	1	1	1	1	1	1	1	1	1	1	1	1	1	1	1	1	1	1	1	1	1	1
SAL0000288	group_1794	putative tautomerase	1	1	1	1	1	1	1	1	1	1	1	1	1	1	1	1	1	1	1	1	1	1	1	1	1	1	1	1	1	1	1	1	1	1	1	1	1	1	1	1	1
SAL0000289	glpF	Glycerol uptake facilitator protein	1	1	1	1	1	1	1	1	1	1	1	1	1	1	1	1	1	1	1	1	1	1	1	1	1	1	1	1	1	1	1	1	1	1	1	1	1	1	1	1	1
SAL0000290	rpmGA	50S ribosomal protein L33 1	1	1	1	1	1	1	1	1	1	1	1	1	1	1	1	1	1	1	1	1	1	1	1	1	1	1	1	1	1	1	1	1	1	1	1	1	1	1	1	1	1
SAL0000291	steT_3	Serine/threonine exchanger SteT	1	1	1	1	1	1	1	1	1	1	1	3	1	1	1	1	1	1	1	1	1	1	1	1	1	1	1	1	1	1	1	1	1	1	1	1	1	1	1	1	2
SAL0000292	ybjI_1	Flavin mononucleotide phosphatase YbjI	1	1	1	1	1	1	1	1	1	1	1	1	1	1	1	1	1	1	1	1	1	1	1	1	1	1	1	1	1	1	1	1	1	1	1	1	1	1	1	1	1
SAL0000293	atpF	ATP synthase subunit b	1	1	1	1	1	1	1	1	1	1	1	1	1	1	1	1	1	1	1	1	1	1	1	1	1	1	1	1	1	1	1	1	1	1	1	1	1	1	1	1	2
SAL0000294	hp	hypothetical protein	1	1	1	1	1	1	1	1	1	1	1	1	1	1	1	1	1	1	1	1	1	1	1	1	1	1	1	1	1	1	1	1	1	1	1	1	1	1	1	1	1
SAL0000295	recU	Holliday junction resolvase RecU	1	1	1	1	1	1	1	1	1	1	1	1	1	1	1	1	1	1	1	1	1	1	1	1	1	1	1	1	1	1	1	1	1	1	1	1	1	1	1	1	2
SAL0000296	hrcA	Heat-inducible transcription repressor HrcA	1	1	1	1	1	1	1	1	1	1	1	1	1	1	1	1	1	1	1	1	1	1	1	1	1	1	1	1	1	1	1	1	1	1	1	1	1	1	1	1	2
SAL0000297	asnB	Asparagine synthetase [glutamine-hydrolyzing] 1	1	1	1	2	1	1	3	1	1	3	1	1	1	1	1	1	1	1	1	1	1	1	1	1	1	1	2	1	1	1	1	1	1	1	1	1	1	1	1	1	4
SAL0000298	hp	hypothetical protein	1	1	1	1	1	1	1	1	1	1	1	1	1	1	1	1	1	1	1	1	1	1	1	1	1	1	1	1	1	1	1	1	1	1	1	1	1	1	1	1	1
SAL0000299	hp	hypothetical protein	1	1	1	1	1	1	1	1	1	1	1	1	1	1	1	1	1	1	1	1	1	1	1	1	1	1	1	1	1	1	1	1	1	1	1	1	1	1	1	1	1
SAL0000300	group_1806	Luciferase-like monooxygenase	1	1	1	1	1	1	1	1	1	1	1	1	1	1	1	1	1	1	1	1	1	1	1	1	1	1	1	1	1	1	1	1	1	1	1	1	1	1	1	1	1
SAL0000301	hp	hypothetical protein	1	1	1	1	1	1	1	1	1	1	1	1	1	1	1	1	1	1	1	1	1	1	1	1	1	1	1	1	1	1	1	1	1	1	1	1	1	1	1	1	1
SAL0000302	carA_1	Carbamoyl-phosphate synthase small chain	1	1	1	1	1	1	1	1	1	1	1	1	1	1	1	1	1	1	1	1	1	1	1	1	1	1	1	1	1	1	1	1	1	1	1	1	1	1	1	1	2
SAL0000303	pgi	Glucose-6-phosphate isomerase	1	1	1	1	1	1	1	1	1	1	1	1	1	1	1	1	1	1	1	1	1	1	1	1	1	1	1	1	1	1	1	1	1	1	1	1	1	1	1	1	2
SAL0000304	group_1810	formimidoylglutamase	1	1	1	1	1	1	1	1	1	1	1	1	1	1	1	1	1	1	1	1	1	1	1	1	1	1	1	1	1	1	1	1	1	1	1	1	1	1	1	1	1
SAL0000305	spo0C	Chromosome-partitioning protein Spo0J	1	1	1	1	1	1	1	1	1	1	1	1	1	1	1	1	1	1	1	1	1	1	1	1	1	1	1	1	1	1	1	1	1	1	1	1	1	1	1	1	2
SAL0000306	acpP	Acyl carrier protein	1	1	1	1	1	1	1	1	1	1	1	1	1	1	1	1	1	1	1	1	1	1	1	1	1	1	1	1	1	1	1	1	1	1	1	1	1	1	1	1	1
SAL0000307	brnQ_1	Branched-chain amino acid transport system 2 carrier protein	1	1	1	1	1	1	1	2	1	1	1	1	1	1	1	1	1	1	1	1	1	1	1	1	1	1	1	1	1	1	1	1	1	1	1	1	1	1	1	1	3
SAL0000308	hp	hypothetical protein	1	1	1	1	1	1	1	1	1	1	1	1	1	1	1	1	1	1	1	1	1	1	1	1	1	1	1	1	1	1	1	1	1	1	1	1	1	1	1	1	1
SAL0000309	hp	hypothetical protein	1	1	1	1	1	1	1	1	1	1	1	1	1	1	1	1	1	1	1	1	1	1	1	1	1	1	1	1	1	1	1	1	1	1	1	1	1	1	1	1	2
SAL0000310	metG	Methionine--tRNA ligase	1	1	1	1	1	1	1	1	1	1	1	1	1	1	1	1	1	1	1	1	1	1	1	1	1	1	1	1	1	1	1	1	1	1	1	1	1	1	1	1	2
SAL0000311	cspA	Cold shock-like protein CspA	1	1	1	1	1	1	1	1	1	1	1	1	1	1	1	1	1	1	1	1	1	1	1	1	1	1	1	1	1	1	1	1	1	1	1	1	1	1	1	1	1
SAL0000312	group_1818	cell division protein MraZ	1	1	1	1	1	1	1	2	1	1	1	1	1	1	1	1	1	1	1	1	1	1	1	1	1	1	1	1	1	1	1	1	1	1	1	1	1	1	1	1	3

SAL0000313	hp	hypothetical protein	1	1	1	1	1	1	1	1	1	1	1	1	1	1	1	1	1	1	1	1	1	1	1	1	1	1	1	1	1	1	1	1	1	1	1	1	1	1	1	1	1
SAL0000314	rluD_3	Ribosomal large subunit pseudouridine synthase D	1	1	1	1	1	1	1	1	1	1	1	1	1	1	1	1	1	1	1	1	1	1	1	1	1	1	1	1	1	1	1	1	1	1	1	1	1	1	1	1	1
SAL0000315	hp	hypothetical protein	1	1	1	1	1	1	1	1	1	1	1	1	1	1	1	1	1	1	1	1	1	1	1	1	1	1	1	1	1	1	1	1	1	1	1	1	1	1	1	1	1
SAL0000316	tsaE	tRNA threonylcarbamoyladenosine biosynthesis protein TsaE	1	1	1	1	1	1	1	1	1	1	1	1	1	1	1	1	1	1	1	1	1	1	1	1	1	1	1	1	1	1	1	1	1	1	1	1	1	1	1	1	1
SAL0000317	rplV	50S ribosomal protein L22	1	1	1	1	1	1	1	1	1	1	1	1	1	1	1	1	1	1	1	1	1	1	1	1	1	1	1	1	1	1	1	1	1	1	1	1	1	1	1	1	1
SAL0000318	ybiV	Sugar phosphatase YbiV	1	1	1	1	1	1	1	1	1	1	1	1	1	1	1	1	1	1	1	1	1	1	1	1	1	1	1	1	1	1	1	1	1	1	1	1	1	1	1	1	1
SAL0000319	xerC_1	Tyrosine recombinase XerC	1	1	1	2	1	1	1	1	1	1	1	1	1	1	1	1	1	1	1	1	1	1	1	1	1	1	2	1	1	1	1	1	1	1	1	1	1	1	1	1	1
SAL0000320	rsmD	Ribosomal RNA small subunit methyltransferase D	1	1	1	1	1	1	1	1	1	1	1	1	1	1	1	1	1	1	1	1	1	1	1	1	1	1	1	1	1	1	1	1	1	1	1	1	1	1	1	1	1
SAL0000321	rplGA	putative ribosomal protein YlxQ	1	1	1	1	1	1	1	1	1	1	1	1	1	1	1	1	1	1	1	1	1	1	1	1	1	1	1	1	1	1	1	1	1	1	1	1	1	1	1	1	1
SAL0000322	group_1828	nitroreductase A	1	1	1	1	1	1	1	1	1	1	1	1	1	1	1	1	1	1	1	1	1	1	1	1	1	1	1	1	1	1	1	1	1	1	1	1	1	1	1	1	1
SAL0000323	pspA_1	Phosphoserine phosphatase 1	1	1	1	1	1	1	1	1	1	1	1	1	1	1	1	1	1	1	1	1	1	1	1	1	1	1	1	1	1	1	1	1	1	1	1	1	1	1	1	1	1
SAL0000324	rplC	50S ribosomal protein L3	1	1	1	1	1	1	1	1	1	1	1	1	1	1	1	1	1	1	1	1	1	1	1	1	1	1	1	1	1	1	1	1	1	1	1	1	1	1	1	1	2
SAL0000325	group_1831	anaerobic benzoate catabolism transcriptional regulator	1	1	1	1	1	1	1	1	1	1	1	1	1	1	1	1	1	1	1	1	1	1	1	1	1	1	1	1	1	1	1	1	1	1	1	1	1	1	1	1	1
SAL0000326	group_1834	Helix-turn-helix	1	1	1	1	1	1	1	1	1	1	1	1	1	1	1	1	1	1	1	1	1	1	1	1	1	1	1	1	1	1	1	1	1	1	1	1	1	1	1	1	1
SAL0000327	hp	hypothetical protein	1	1	1	1	1	1	1	1	1	1	1	1	1	1	1	1	1	1	1	1	1	1	1	1	1	1	1	1	1	1	1	1	1	1	1	1	1	1	1	1	1
SAL0000328	cggR	Central glycolytic genes regulator	1	1	1	1	1	1	1	1	1	1	1	2	1	1	1	1	1	1	1	1	1	1	1	1	1	1	1	1	1	1	1	1	1	1	1	1	1	1	1	1	1
SAL0000329	group_1837	glutamine amidotransferase	1	1	1	1	1	1	1	1	1	1	1	1	1	1	1	1	1	1	1	1	1	1	1	1	1	1	1	1	1	1	1	1	1	1	1	1	1	1	1	1	1
SAL0000330	yghB	Inner membrane protein YghB	1	1	1	1	1	1	1	1	1	1	1	2	1	1	1	1	1	1	1	1	1	1	1	1	1	1	1	1	1	1	1	1	1	1	1	1	1	1	1	1	1
SAL0000331	group_1839	Oleate hydratase	1	1	1	1	1	1	1	1	1	1	1	1	1	1	1	1	1	1	1	1	1	1	1	1	1	1	1	1	1	1	1	1	1	1	1	1	1	1	1	1	2
SAL0000332	hp	hypothetical protein	1	1	1	1	1	1	1	1	1	1	1	1	1	1	1	1	1	1	1	1	1	1	1	1	1	1	1	1	1	1	1	1	1	1	1	1	1	1	1	1	1
SAL0000333	hp	hypothetical protein	1	1	1	1	1	1	1	1	1	1	1	1	1	1	1	1	1	1	1	1	1	1	1	1	1	1	1	1	1	1	1	1	1	1	1	1	1	1	1	1	1
SAL0000334	oppA_1	Periplasmic oligopeptide-binding protein precursor	1	1	1	1	1	1	1	1	1	1	1	1	1	1	1	1	1	1	1	1	1	1	1	1	1	1	1	1	1	1	1	1	1	1	1	1	1	1	1	1	1
SAL0000335	pepC	Aminopeptidase C	1	1	1	1	1	1	1	1	1	1	1	1	1	1	1	1	1	1	1	1	1	1	1	1	1	1	1	1	1	1	1	1	1	1	1	1	1	1	1	1	2
SAL0000336	hp	hypothetical protein	1	1	1	1	1	1	1	1	1	1	1	1	1	1	1	1	1	1	1	1	1	1	1	1	1	1	1	1	1	1	1	1	1	1	1	1	1	1	1	1	1
SAL0000337	malP_1	PTS system maltose-specific EIICB component	1	1	1	1	1	2	1	1	1	1	1	1	1	1	1	1	1	1	1	1	1	1	1	1	1	1	1	1	1	1	1	1	1	1	1	1	1	1	1	1	1
SAL0000338	hp	hypothetical protein	1	1	1	1	1	1	1	1	1	1	1	1	1	1	1	1	1	1	1	1	1	1	1	1	1	1	1	1	1	1	1	1	1	1	1	1	1	1	1	1	2
SAL0000339	xseB	Exodeoxyribonuclease 7 small subunit	1	1	1	1	1	1	1	1	1	1	1	1	1	1	1	1	1	1	1	1	1	1	1	1	1	1	1	1	1	1	1	1	1	1	1	1	1	1	1	1	1
SAL0000340	group_1850	Bacterial extracellular solute-binding proteins, family 5 Middle	1	1	1	1	1	1	1	1	1	1	1	1	1	1	1	1	1	1	1	1	1	1	1	1	1	1	1	1	1	1	1	1	1	1	1	1	1	1	1	1	1
SAL0000341	ykoE	Putative HMP/thiamine permease protein YkoE	1	1	1	1	1	1	1	1	1	1	1	1	1	1	1	1	1	1	1	1	1	1	1	1	1	1	1	1	1	1	1	1	1	1	1	1	1	1	1	1	1
SAL0000342	bmr3_1	Multidrug resistance protein 3	1	1	1	1	1	1	1	1	1	1	1	1	1	1	1	1	1	1	1	1	1	1	1	1	1	1	1	1	1	1	1	1	1	1	1	1	1	1	1	1	2
SAL0000343	hp	hypothetical protein	1	1	1	1	1	1	1	1	1	1	1	1	1	1	1	1	1	1	1	1	1	1	1	1	1	1	1	1	1	1	1	1	1	1	1	1	1	1	1	1	1
SAL0000344	aspS	Aspartate--tRNA ligase	1	1	1	1	1	1	1	1	1	1	1	1	1	1	1	1	1	1	1	1	1	1	1	1	1	1	1	1	1	1	1	1	1	1	1	1	1	1	1	1	1
SAL0000345	cbh_2	Choloylglycine hydrolase	1	1	1	1	1	1	1	1	1	1	1	1	1	1	1	1	1	1	1	1	1	1	1	1	1	1	1	1	1	1	1	1	1	1	1	1	1	1	1	1	1
SAL0000346	hp	hypothetical protein	1	1	1	1	1	1	1	1	1	1	1	1	1	1	1	1	1	1	1	1	1	1	1	1	1	1	1	1	1	1	1	1	1	1	1	1	1	1	1	1	1
SAL0000347	potB	Spermidine/putrescine transport system permease protein PotB	1	1	1	1	1	1	1	1	1	1	1	1	1	1	1	1	1	1	1	1	1	1	1	1	1	1	1	1	1	1	1	1	1	1	1	1	1	1	1	1	1
SAL0000348	hp	hypothetical protein	1	1	1	1	1	1	1	1	1	1	1	1	1	1	1	1	1	1	1	1	1	1	1	1	1	1	1	1	1	1	1	1	1	1	1	1	1	1	1	1	1
SAL0000349	hp	hypothetical protein	1	1	1	1	1	1	1	1	1	1	1	1	1	1	1	1	1	1	1	1	1	1	1	1	1	1	3	1	1	1	1	1	1	1	1	1	1	1	1	1	2
SAL0000350	group_186	drug efflux system protein MdtG	1	1	1	1	1	1	1	1	1	1	1	1	1	1	1	1	1	1	1	1	1	1	1	1	1	1	1	1	1	1	1	1	1	1	1	1	1	1	1	1	2
SAL0000351	hp	hypothetical protein	1	1	1	1	1	1	1	1	1	1	1	1	1	1	1	1	1	1	1	1	1	1	1	1	1	1	1	1	1	1	1	1	1	1	1	1	1	1	1	1	1
SAL0000352	group_1861	Uracil DNA glycosylase superfamily protein	1	1	1	1	1	1	1	1	1	1	1	1	1	1	1	1	1	1	1	1	1	1	1	1	1	1	1	1	1	1	1	1	1	1	1	1	1	1	1	1	1
SAL0000353	yvyD	Putative sigma-54 modulation protein	1	1	1	1	1	1	1	1	1	1	1	1	1	1	1	1	1	1	1	1	1	1	1	1	1	1	1	1	1	1	1	1	1	1	1	1	1	1	1	1	2
SAL0000354	hup	DNA-binding protein HU	1	1	1	1	1	1	1	1	1	1	1	1	1	1	1	1	1	1	1	1	1	1	1	1	1	1	1	1	1	1	1	1	1	1	1	1	1	1	1	1	1
SAL0000355	group_1864	putative HTH-type transcriptional regulator	1	1	1	1	1	1	1	1	1	1	1	1	1	1	1	1	1	1	1	1	1	1	1	1	1	1	1	1	1	1	1	1	1	1	1	1	1	1	1	1	2
SAL0000356	ldh_1	L-lactate dehydrogenase	1	1	1	1	1	1	1	1	1	1	1	1	1	1	1	1	1	1	1	1	1	1	1	1	1	1	1	1	1	1	1	1	1	1	1	1	1	1	1	1	1
SAL0000357	prmC	Release factor glutamine methyltransferase	1	1	1	1	1	1	1	1	1	1	1	1	1	1	1	1	1	1	1	1	1	1	1	1	1	1	1	1	1	1	1	1	1	1	1	1	1	1	1	1	2
SAL0000358	group_1867	MarR family protein	1	1	1	1	1	1	1	1	1	1	1	1	1	1	1	1	1	1	1	1	1	1	1	1	1	1	1	1	1	1	1	1	1	1	1	1	1	1	1	1	1
SAL0000359	group_1868	UDP-N-acetylmuramate--L-alanine ligase	1	1	1	1	1	1	1	1	1	1	1	1	1	1	1	1	1	1	1	1	1	1	1	1	1	1	1	1	1	1	1	1	1	1	1	1	1	1	1	1	1
SAL0000360	group_1869	GtrA-like protein	1	1	1	1	1	1	1	3	1	1	1	1	1	1	1	1	1	1	1	1	1	1	1	1	1	1	1	1	1	1	1	1	1	1	1	1	1	1	1	1	2
SAL0000361	hp	hypothetical protein	1	1	1	1	1	1	1	1	1	1	1	1	1	1	1	1	1	1	1	1	1	1	1	1	1	1	1	1	1	1	1	1	1	1	1	1	1	1	1	1	1
SAL0000362	rplD	50S ribosomal protein L4	1	1	1	1	1	1	1	1	1	1	1	1	1	1	1	1	1	1	1	1	1	1	1	1	1	1	1	1	1	1	1	1	1	1	1	1	1	1	1	1	1
SAL0000363	rplA	50S ribosomal protein L1	1	1	1	1	1	1	1	1	1	1	1	1	1	1	1	1	1	1	1	1	1	1	1	1	1	1	1	1	1	1	1	1	1	1	1	1	1	1	1	1	1
SAL0000364	hp	hypothetical protein	1	1	1	1	1	1	1	1	1	1	1	1	1	1	1	1	1	1	1	1	1	1	1	1	1	1	1	1	1	1	1	1	1	1	1	1	1	1	1	1	1
SAL0000365	ptsH	Phosphocarrier protein HPr	1	1	1	1	1	1	1	1	1	1	1	1	1	1	1	1	1	1	1	1	1	1	1	1	1	1	1	1	1	1	1	1	1	1	1	1	1	1	1	1	1
SAL0000366	cpoA	Alpha-galactosylglucosyldiacylglycerol synthase	1	1	1	1	1	1	1	1	1	1	1	1	1	1	1	1	1	1	1	1	1	1	1	1	1	1	1	1	1	1	1	1	1	1	1	1	1	1	2	1	1
SAL0000367	scpB	Segregation and condensation protein B	1	1	1	1	1	1	1	1	1	1	1	1	1	1	1	1	1	1	1	1	1	1	1	1	1	1	1	1	1	1	1	1	1	1	1	1	1	1	1	1	2
SAL0000368	fpaP_1	Proline iminopeptidase	1	1	1	1	1	1	1	1	1	1	1	1	1	1	1	1	1	1	1	1	1	1	1	1	1	1	1	1	1	1	1	1	1	1	1	1	1	1	1	1	1
SAL0000369	uppS	Ditrans,polycis-undecaprenyl-diphosphate synthase ((2E,6E)-farnesyl-diphosp	1	1	1	1	1	1	1	1	1	1	1	1	1	1	1	1	1	1	1	1	1	1	1	1	1	1	1	1	1	1	1	1	1	1	1	1	1	1	1	1	1
SAL0000370	bceA	Bacitracin export ATP-binding protein BceA	1	1	1	1	1	1	1	1	1	1	1	1	1	1	1	1	1	1	1	1	1	1	1	1	1	1	1	1	1	1	1	1	1	1	1	1	1	1	1	1	1
SAL0000371	atpB	ATP synthase subunit a	1	1	1	1	1	1	1	1	1	1	1	1	1	1	1	1	1	1	1	1	1	1	1	1	1	1	1	1	1	1	1	1	1	1	1	1	1	1	1	1	2
SAL0000372	mutT4	Putative mutator protein MutT4	1	1	1	1	1	1	1	1	1	1	1	1	1	1	1	1	1	1	1	1	1	1	1	1	1	1	1	1	1	1	1	1	1	1	1	1	1	1	1	1	2
SAL0000373	guaA	GMP synthase [glutamine-hydrolyzing]	1	1	1	1	1	2	1	1	1	1	1	1	1	1	1	1	1	1	1	1	1	1	1	1	1	1	1	1	1	1	1	1	1	1	1	1	1	1	1	1	3
SAL0000374	efp_2	Elongation factor P	1	1	1	1	1	1	1	1	1	1	1	1	1	1	1	1	1	1	1	1	1	1	1	1	1	1	1	1	3	1	1	1	1	1	1	1	1	1	1	1	2
SAL0000375	pncB2	Nicotinate phosphoribosyltransferase pncB2	1	1	1	1	1	1	1	1	1	1	1	2	1	1	1	1	1	1	1	1	1	1	1	1	1	1	1	1	3	1	1	1	1	1	1	1	1	1	1	1	4
SAL0000376	group_1885	Glutamine transport system permease protein GlnP	1	1	1	1	1	1	1	2	1	1	1	1	1	1	1	1	1	1	1	1	1	1	1	1	1	1	1	1	1	1	1	1	1	1	1	1	1	1	1	1	1

SAL0000377	hp	hypothetical protein	1	1	2	1	1	1	1	1	1	1	1	1	1	1	2	1	1	1	1	1	1	1	1	1	1	1	1	1	1	2	1	2	1	1	1	1	1	1	1	1	1
SAL0000378	atpD	ATP synthase subunit beta	1	1	2	1	1	1	1	2	1	1	1	1	1	1	2	1	1	1	1	1	1	1	1	1	1	1	1	2	1	2	1	2	1	1	1	3	1	1	1	1	4
SAL0000379	glcR	HTH-type transcriptional repressor GlcR	1	1	1	1	1	1	1	1	1	1	1	1	1	1	1	1	1	1	1	1	1	1	1	1	1	1	1	1	1	1	1	1	1	1	1	1	1	1	1	1	2
SAL0000380	group_1889	Helix-turn-helix domain protein	1	1	1	1	1	1	1	1	1	1	1	1	1	1	1	1	1	1	1	1	1	1	1	1	1	1	1	1	1	1	1	1	1	1	1	1	1	1	1	1	2
SAL0000381	group_1890	pheromone autoinducer 2 transporter	1	1	1	1	1	1	1	1	1	1	1	1	1	1	1	1	1	1	1	1	1	1	1	1	1	1	1	1	1	1	1	1	1	1	1	1	1	1	1	1	2
SAL0000382	group_1891	4-diphosphocytidyl-2-C-methyl-D-erythritol kinase	1	1	1	1	1	1	1	1	1	1	1	1	1	1	1	1	1	1	1	1	1	1	1	1	1	1	1	1	1	1	1	1	1	1	1	1	1	1	1	1	2
SAL0000383	ywpJ	Putative phosphatase YwpJ	1	1	1	1	1	1	1	3	1	1	1	1	1	1	1	1	1	1	1	1	1	1	1	1	1	1	1	1	1	1	1	1	1	1	1	1	1	1	1	1	2
SAL0000384	ltaS2	Lipoteichoic acid synthase 2	1	1	2	1	1	1	3	1	1	3	1	1	1	1	2	1	1	1	1	1	1	1	1	1	1	1	1	1	5	2	1	2	1	1	1	1	1	1	1	1	4
SAL0000385	gtfA_1	Sucrose phosphorylase	1	1	1	1	1	1	1	1	1	1	1	3	1	1	1	1	1	1	1	1	1	1	1	1	1	1	1	1	1	1	1	1	1	1	1	1	1	1	1	1	2
SAL0000386	hp	hypothetical protein	1	1	1	1	1	1	1	1	1	1	1	1	1	1	1	1	1	1	1	1	1	1	1	1	1	1	1	1	1	1	1	1	1	1	1	1	1	1	1	2	1
SAL0000387	hp	hypothetical protein	1	1	1	1	1	1	1	1	1	1	1	1	1	1	1	1	1	1	1	1	1	1	1	1	1	1	1	1	1	1	1	1	1	1	1	1	1	1	1	1	2
SAL0000388	sdhB	L-serine dehydratase, beta chain	1	1	6	1	1	1	1	3	1	1	1	1	1	1	1	1	1	1	1	1	1	1	1	1	1	1	1	2	5	1	1	1	1	1	1	1	1	1	1	1	4
SAL0000389	group_1898	putative nicotinate-nucleotide adenylyltransferase	1	1	1	1	1	1	1	3	1	1	1	1	1	1	1	1	1	1	1	1	1	1	1	1	1	1	1	1	1	1	1	1	1	1	1	1	1	1	1	1	2
SAL0000390	bglF_3	PTS system beta-glucoside-specific EIIBCA component	1	1	1	1	1	1	1	2	1	1	1	1	1	1	1	1	1	1	1	1	1	1	1	1	1	1	1	1	1	1	1	1	1	1	1	1	1	1	1	1	3
SAL0000391	group_1900	D-aspartate ligase	1	1	1	1	1	1	1	1	1	1	1	1	1	1	1	1	1	1	1	1	1	1	1	1	1	1	1	1	1	1	1	1	1	1	1	1	1	1	1	1	2
SAL0000392	rpsG	30S ribosomal protein S7	1	1	2	1	1	1	1	2	1	1	1	1	1	1	2	1	1	1	1	1	1	1	1	1	1	1	1	2	1	2	1	2	1	1	1	1	1	1	1	1	1
SAL0000393	potD	Spermidine/putrescine-binding periplasmic protein precursor	1	1	1	1	1	4	1	1	1	1	1	1	1	1	1	1	1	1	1	1	1	1	1	1	1	1	1	3	1	1	1	1	1	1	1	1	1	1	1	1	2
SAL0000394	hp	hypothetical protein	1	1	1	1	1	1	1	1	1	1	1	1	1	1	1	1	1	1	1	1	1	1	1	1	1	1	1	1	1	1	1	1	1	1	1	1	1	1	1	1	2
SAL0000395	group_1904	N-acetyldiaminopimelate deacetylase	1	1	1	1	1	1	1	1	1	1	1	1	1	1	1	1	1	1	1	1	1	1	1	1	1	1	1	1	1	1	1	1	1	1	1	1	1	1	1	1	2
SAL0000396	yicI_3	Alpha-xylosidase	1	1	2	3	1	2	2	6	1	2	1	4	1	1	2	1	1	1	1	1	1	1	1	1	1	1	3	2	2	2	1	2	1	1	1	1	1	1	1	1	5
SAL0000397	group_1906	Peptidase propeptide and YPEB domain protein	1	1	1	1	1	1	1	1	1	1	1	1	1	1	1	1	1	1	1	1	1	1	1	1	1	1	1	1	1	1	1	1	1	1	1	1	1	1	1	1	2
SAL0000398	dnaE	DNA polymerase III subunit alpha	1	1	3	1	1	6	1	7	1	1	1	1	1	1	2	1	1	1	1	1	1	1	1	1	1	1	4	1	1	2	1	2	1	1	1	1	1	1	1	1	5
SAL0000399	group_1908	putative acetyltransferase	1	1	1	1	1	1	1	1	1	1	1	2	1	1	1	1	1	1	1	1	1	1	1	1	1	1	1	1	1	1	1	1	1	1	1	1	1	1	1	1	3
SAL0000400	hp	hypothetical protein	1	1	2	1	1	1	1	1	1	1	1	1	1	1	1	1	1	1	1	1	1	1	1	1	1	1	1	1	1	1	1	1	1	1	1	1	1	1	1	1	1
SAL0000401	group_1910	DNA polymerase III subunit delta	1	1	1	1	1	1	1	2	1	1	1	1	1	1	1	1	1	1	1	1	1	1	1	1	1	1	1	2	3	1	1	1	1	1	1	1	1	1	1	1	1
SAL0000402	yvoA_1	HTH-type transcriptional repressor YvoA	1	1	1	1	1	1	1	1	1	1	1	2	1	1	1	1	1	1	1	1	1	1	1	1	1	1	1	1	1	1	1	1	1	1	1	1	1	1	1	1	3
SAL0000403	mnmA	tRNA-specific 2-thiouridylase MnmA	1	1	1	1	1	1	1	1	1	1	1	1	1	1	1	1	1	1	1	1	1	1	1	1	1	1	1	1	2	1	1	1	1	1	1	1	1	1	1	1	1
SAL0000404	hp	hypothetical protein	1	1	2	1	1	1	1	2	1	1	1	1	1	1	2	1	1	1	1	1	1	1	1	1	1	1	1	2	1	2	1	2	1	1	1	1	1	1	1	1	1
SAL0000405	hp	hypothetical protein	1	1	1	1	1	1	1	1	1	1	1	1	1	1	1	1	1	1	1	1	1	1	1	1	1	1	1	1	1	1	1	1	1	1	1	1	1	1	1	1	2
SAL0000406	hp	hypothetical protein	1	1	1	1	1	1	1	3	1	1	1	1	1	1	1	1	1	1	1	1	1	1	1	1	1	1	1	1	1	1	1	1	1	1	1	1	1	1	1	1	2
SAL0000407	rplJ	50S ribosomal protein L10	1	1	1	1	1	1	2	1	1	2	1	1	1	1	1	1	1	1	1	1	1	1	1	1	1	1	1	1	1	1	1	1	1	1	1	1	1	1	1	1	3
SAL0000408	clpP	ATP-dependent Clp protease proteolytic subunit	1	1	1	1	1	1	1	1	1	1	1	1	1	1	1	1	1	1	1	1	1	1	1	1	1	1	1	1	1	1	1	1	1	1	1	1	1	1	1	1	2
SAL0000409	penA	Penicillin-binding protein 2B	1	1	1	1	1	3	1	1	1	1	1	1	1	1	1	1	1	1	1	1	1	1	1	1	1	1	1	1	1	1	1	1	1	1	1	1	1	1	1	1	2
SAL0000410	purN	Phosphoribosylglycinamide formyltransferase	1	1	1	1	1	1	1	1	1	2	1	1	1	1	1	1	1	1	1	1	1	1	1	1	1	1	1	1	1	1	1	1	1	1	1	1	1	1	1	1	3
SAL0000411	pbpF	Penicillin-binding protein 1F	1	1	1	2	1	1	1	1	1	1	1	1	1	1	1	1	1	1	1	1	1	1	1	1	1	1	1	1	1	1	1	1	1	1	1	1	1	1	1	1	3
SAL0000412	yheS_3	putative ABC transporter ATP-binding protein YheS	1	1	1	1	1	1	1	2	1	1	1	1	1	1	1	1	1	1	1	1	1	1	1	1	1	1	1	1	1	1	1	1	1	1	1	1	1	1	1	1	3
SAL0000413	yedK	Putative SOS response-associated peptidase YedK	1	1	1	1	1	1	1	1	1	1	1	1	1	1	1	1	1	1	1	1	1	1	1	1	1	1	1	1	2	1	1	1	1	1	1	1	1	1	1	1	1
SAL0000414	group_1924	Putative protein phosphatase 2C-type	1	1	1	1	1	1	1	1	1	1	1	1	1	1	1	1	1	1	1	1	1	1	1	1	1	1	1	2	4	1	1	1	1	1	1	1	1	1	1	1	3
SAL0000415	group_1925	Acyltransferase family protein	1	1	1	1	1	1	1	1	1	1	1	1	1	1	1	1	1	1	1	1	1	1	1	1	1	1	1	1	1	1	1	1	1	1	1	1	1	1	1	1	2
SAL0000416	ligA	DNA ligase	1	1	1	1	1	1	1	1	1	1	1	1	1	1	1	1	1	1	1	1	1	1	1	1	1	1	1	1	1	1	1	1	1	1	1	1	1	1	1	1	2
SAL0000417	cycA_1	D-serine/D-alanine/glycine transporter	1	1	1	1	1	1	1	2	1	1	1	1	1	1	1	1	1	1	1	1	1	1	1	1	1	1	1	1	4	1	1	1	1	1	1	1	1	1	1	1	3
SAL0000418	smc	Chromosome partition protein Smc	1	1	1	1	1	3	1	4	1	1	1	2	1	1	1	1	1	1	1	1	1	1	1	1	1	1	1	1	1	1	1	1	1	1	1	1	1	1	1	1	5
SAL0000419	rodA	Rod shape-determining protein RodA	1	1	1	1	1	1	1	1	1	1	1	1	1	1	1	1	1	1	1	1	1	1	1	1	1	1	1	1	2	1	1	1	1	1	1	1	1	1	1	1	3
SAL0000420	obg	GTPase Obg	1	1	1	1	1	1	2	1	1	2	1	4	1	1	1	1	1	1	1	1	1	1	1	1	1	1	1	1	1	1	1	1	1	1	1	1	1	1	1	1	3
SAL0000421	group_1931	DegV domain-containing protein	1	1	1	1	1	1	1	1	1	1	1	1	1	1	1	1	1	1	1	1	1	1	1	1	1	1	1	1	1	1	1	1	1	1	1	1	1	1	1	1	2
SAL0000422	miaA	tRNA dimethylallyltransferase	1	1	2	1	1	1	1	2	1	1	1	1	1	1	2	1	1	1	1	1	1	1	1	1	1	1	1	2	1	2	1	2	1	1	1	1	1	1	1	1	1
SAL0000423	hp	hypothetical protein	1	1	1	1	1	1	1	1	1	1	1	1	1	1	1	1	1	1	1	1	1	1	1	1	1	1	2	1	1	1	1	1	1	1	1	1	1	1	1	1	3
SAL0000424	group_1934	putative acetyltransferase	1	1	1	1	1	1	1	1	1	1	1	1	1	1	1	1	1	1	1	1	1	1	1	1	1	1	1	1	1	1	1	1	1	1	1	1	1	1	1	1	2
SAL0000425	group_1935	type IV pilin biogenesis protein	1	1	1	1	1	1	1	1	1	1	1	1	1	1	1	1	1	1	1	1	1	1	1	1	1	1	1	1	1	1	1	1	1	1	1	1	1	1	1	1	2
SAL0000426	ackA_2	Acetate kinase	1	1	1	1	1	1	1	1	1	1	1	3	1	1	2	1	1	1	1	1	1	1	1	1	1	1	1	1	1	2	1	2	1	1	1	1	1	1	1	1	4
SAL0000427	plsC	1-acyl-sn-glycerol-3-phosphate acyltransferase	1	1	1	1	1	1	1	1	1	1	1	1	1	1	1	1	1	1	1	1	1	1	1	1	1	1	1	1	1	1	1	1	1	1	1	1	1	1	1	1	2
SAL0000428	yrrK	Putative Holliday junction resolvase	1	1	1	1	1	1	1	2	1	1	1	1	1	1	1	1	1	1	1	1	1	1	1	1	1	1	1	1	1	1	1	1	1	1	1	1	1	1	1	1	1
SAL0000429	pepX	Xaa-Pro dipeptidyl-peptidase	1	1	4	1	1	1	3	1	1	3	1	1	1	1	2	1	1	1	1	1	1	1	1	1	1	1	1	1	6	2	1	2	1	1	1	1	1	1	1	1	5
SAL0000430	der	GTPase Der	1	1	1	1	1	1	1	1	1	1	1	2	1	1	1	1	1	1	1	1	1	1	1	1	1	1	1	1	1	1	1	1	1	1	1	1	1	1	1	1	1
SAL0000431	slpA_3	S-layer protein precursor	1	1	1	1	1	1	1	2	1	1	1	1	1	1	1	1	1	1	1	1	1	1	1	1	1	1	1	3	1	1	1	1	1	1	1	1	1	1	1	1	4
SAL0000432	hflK	Modulator of FtsH protease HflK	1	1	1	1	1	1	1	1	1	1	1	1	1	1	1	1	1	1	1	1	1	1	1	1	1	1	1	3	1	2	1	2	1	1	1	1	1	1	1	1	4
SAL0000433	dnaG	DNA primase	1	1	1	1	1	1	1	1	1	1	1	1	1	1	1	1	1	1	1	1	1	1	1	1	1	1	1	1	3	1	1	1	1	1	1	4	1	1	1	1	2
SAL0000434	hp	hypothetical protein	1	1	1	1	1	1	1	1	1	1	1	1	1	1	1	1	1	1	1	1	1	1	1	1	1	1	1	1	1	1	1	1	1	1	1	1	1	1	1	1	2
SAL0000435	group_1945	cyclic 3',5'-adenosine monophosphate phosphodiesterase	1	1	4	1	1	1	1	1	1	1	1	5	1	1	1	1	1	1	1	1	1	1	1	1	1	1	1	1	3	1	1	1	1	1	1	1	1	1	1	1	2
SAL0000436	iap	putative endopeptidase p60 precursor	1	1	1	1	1	1	1	1	1	1	1	3	1	1	1	1	1	1	1	1	1	1	1	1	1	1	1	1	1	1	1	1	1	1	1	1	1	1	1	1	2
SAL0000437	divIVA	Septum site-determining protein DivIVA	1	1	1	1	1	1	1	4	1	1	1	2	1	1	1	1	1	1	1	1	1	1	1	1	1	1	1	3	1	1	1	1	1	1	1	1	1	1	1	1	5
SAL0000438	yicL_3	putative inner membrane transporter YicL	1	1	1	1	1	1	1	1	1	1	1	1	1	1	1	1	1	1	1	1	1	1	1	1	1	1	1	1	1	1	1	1	1	1	1	1	1	1	1	1	2
SAL0000439	tadA	tRNA-specific adenosine deaminase	1	1	1	2	1	1	1	1	1	1	1	1	1	1	1	1	1	1	1	1	1	1	1	1	1	1	2	1	1	1	1	1	1	1	1	1	1	1	1	1	3
SAL0000440	polA	DNA polymerase I	1	1	1	1	1	1	1	1	1	1	1	1	1	1	1	1	1	1	1	1	1	1	1	1	1	1	1	1	1	1	1	1	1	1	1	1	1	1	1	1	2

SAL0000441	cshA_1	DEAD-box ATP-dependent RNA helicase CshA	1	1	4	1	1	1	1	2	1	1	1	1	1	1	1	1	1	1	1	1	1	1	1	1	1	1	1	1	1	1	1	1	1	1	1	1	1	1	1	1	3
SAL0000442	corC_2	Magnesium and cobalt efflux protein CorC	1	1	1	1	1	1	1	1	1	1	1	1	1	1	1	1	1	1	1	1	1	1	1	1	1	1	1	1	1	1	1	1	1	1	1	1	1	1	1	1	2
SAL0000443	pyrF	Orotidine 5'-phosphate decarboxylase	1	1	1	1	1	1	1	2	1	1	1	1	1	1	1	1	1	1	1	1	1	1	1	1	1	1	1	1	1	1	1	1	1	1	1	1	1	1	1	1	3
SAL0000444	ynfM	Inner membrane transport protein YnfM	1	1	2	1	1	1	1	1	1	1	1	4	1	1	2	1	1	1	1	1	1	1	1	1	1	1	1	1	1	2	1	2	1	1	1	1	1	1	1	1	3
SAL0000445	group_1955	ASCH domain protein	1	1	1	1	1	1	1	1	1	1	1	1	1	1	1	1	1	1	1	1	1	1	1	1	1	1	1	1	1	1	1	1	1	1	1	2	1	1	1	1	3
SAL0000446	coaBC	Coenzyme A biosynthesis bifunctional protein CoaBC	1	1	1	1	1	1	1	3	1	1	1	1	1	1	1	1	1	1	1	1	1	1	1	1	1	1	1	1	1	1	1	1	1	1	1	1	1	1	1	1	2
SAL0000447	msbA_1	Lipid A export ATP-binding/permease protein MsbA	1	1	2	1	1	2	3	2	1	3	1	5	1	1	2	1	1	1	1	1	1	1	1	1	1	1	1	2	6	2	1	2	1	1	1	1	1	1	1	1	4
SAL0000448	gap	Glyceraldehyde-3-phosphate dehydrogenase	1	1	1	1	1	1	2	1	1	2	1	1	1	1	1	1	1	1	1	1	1	1	1	1	1	1	1	1	1	1	1	1	1	1	1	1	1	1	1	1	1
SAL0000449	adiC	Arginine/agmatine antiporter	1	1	1	1	1	1	1	2	1	1	1	1	1	1	1	1	1	1	1	1	1	1	1	1	1	1	1	2	1	1	1	1	1	1	1	1	1	1	1	1	3
SAL0000450	prfA	Peptide chain release factor 1	1	1	1	1	1	1	1	1	1	1	1	1	1	1	1	1	1	1	1	1	1	1	1	1	1	1	1	1	1	2	1	2	1	1	1	1	1	1	1	1	1
SAL0000451	hp	hypothetical protein	1	1	2	1	1	1	1	1	1	1	1	3	1	1	2	1	1	1	1	1	1	1	1	1	1	1	1	1	1	2	1	2	1	1	1	1	1	1	1	1	4
SAL0000452	prs_1	Ribose-phosphate pyrophosphokinase	1	1	1	1	1	1	1	1	1	1	1	1	1	1	2	1	1	1	1	1	1	1	1	1	1	1	1	1	1	2	1	2	1	1	1	1	1	1	1	1	3
SAL0000453	nrdD	Anaerobic ribonucleoside-triphosphate reductase	1	1	1	1	1	1	1	2	1	1	1	3	1	1	1	1	1	1	1	1	1	1	1	1	1	1	1	1	1	1	1	1	1	1	1	1	1	1	1	1	4
SAL0000454	nadD	Nicotinate-nucleotide adenylyltransferase	1	1	2	1	1	1	3	2	1	3	1	1	1	1	2	1	1	1	1	1	1	1	1	1	1	1	1	2	1	2	1	2	1	1	1	1	1	1	1	1	4
SAL0000455	pstS1	Phosphate-binding protein PstS 1 precursor	1	1	1	1	1	1	1	1	1	1	1	1	1	1	1	1	1	1	1	1	1	1	1	1	1	1	1	1	1	1	1	1	1	1	1	1	1	1	1	1	2
SAL0000456	hp	hypothetical protein	1	1	1	1	1	1	1	4	1	1	1	1	1	1	1	1	1	1	1	1	1	1	1	1	1	1	1	1	2	1	1	1	1	1	1	1	1	1	1	1	3
SAL0000457	hp	hypothetical protein	1	1	1	1	1	1	1	1	1	1	1	1	1	1	1	1	1	1	1	1	1	1	1	1	1	1	1	1	1	1	1	1	1	1	1	1	1	1	1	1	2
SAL0000458	group_1968	Putative phosphatase	1	1	1	1	1	1	2	1	1	2	1	1	1	1	1	1	1	1	1	1	1	1	1	1	1	1	1	1	1	1	1	1	1	1	1	1	1	1	1	1	1
SAL0000459	glyA	Serine hydroxymethyltransferase	1	1	1	1	1	1	1	1	1	1	1	1	1	1	1	1	1	1	1	1	1	1	1	1	1	1	1	1	1	1	1	1	1	1	1	1	1	1	1	1	2
SAL0000460	hp	hypothetical protein	1	1	1	2	1	1	1	2	1	1	1	1	1	1	1	1	1	1	1	1	1	1	1	1	1	1	2	1	1	1	1	1	1	1	1	1	1	1	1	1	3
SAL0000461	oppC_1	Oligopeptide transport system permease protein OppC	1	1	1	1	1	1	1	1	1	1	1	1	1	1	1	1	1	1	1	1	1	1	1	1	1	1	1	1	1	1	1	1	1	1	1	1	1	1	1	1	2
SAL0000462	glnQ_1	Glutamine transport ATP-binding protein GlnQ	1	1	1	1	1	1	1	1	1	1	1	1	1	1	1	1	1	1	1	1	1	1	1	1	1	1	1	1	1	1	1	1	1	1	1	1	1	1	1	1	2
SAL0000463	qacA	Antiseptic resistance protein	1	1	1	1	1	1	1	1	1	1	1	1	1	1	1	1	1	1	1	1	1	1	1	1	1	1	1	1	1	1	1	1	1	1	1	1	1	1	1	1	2
SAL0000464	dapF	Diaminopimelate epimerase	1	1	1	1	1	1	1	1	1	1	1	1	1	1	1	1	1	1	1	1	1	1	1	1	1	1	1	1	1	1	1	1	1	1	1	1	1	1	1	1	2
SAL0000465	bglF_1	PTS system beta-glucoside-specific EIIBCA component	1	1	2	1	1	1	1	3	1	1	1	4	1	1	2	1	1	1	1	1	1	1	1	1	1	1	1	1	1	2	1	2	1	1	1	1	1	1	1	1	5
SAL0000466	appA_2	Oligopeptide-binding protein AppA precursor	1	1	5	1	1	1	3	1	1	3	1	1	1	1	2	1	1	1	1	1	1	1	1	1	1	1	1	1	1	2	1	2	1	1	1	1	1	1	4	1	6
SAL0000467	group_1976	Putative tRNA (cytidine(34)-2'-O)-methyltransferase	1	1	1	1	1	1	1	1	1	1	1	1	1	1	1	1	1	1	1	1	1	1	1	1	1	1	1	1	3	1	1	1	1	1	1	1	1	1	1	1	2
SAL0000468	patA_1	Putative N-acetyl-LL-diaminopimelate aminotransferase	1	1	1	1	1	1	1	2	1	1	1	1	1	1	1	1	1	1	1	1	1	1	1	1	1	1	1	2	1	1	1	1	1	1	1	1	1	1	1	1	3
SAL0000469	rpsC	30S ribosomal protein S3	1	1	1	1	1	1	1	1	1	1	1	1	1	1	1	1	1	1	1	1	1	1	1	1	1	1	1	1	1	1	1	1	1	1	1	2	1	1	1	1	1
SAL0000470	hslV	ATP-dependent protease subunit HslV	1	1	1	1	1	1	1	2	1	1	1	3	1	1	1	1	1	1	1	1	1	1	1	1	1	1	1	2	1	1	1	1	1	1	1	1	1	1	1	1	4
SAL0000471	msmE_2	Multiple sugar-binding protein precursor	1	1	1	1	1	1	1	1	1	1	1	1	1	1	1	1	1	1	1	1	1	1	1	1	1	1	1	1	1	1	1	1	1	1	1	1	1	1	1	1	2
SAL0000472	rpsD	30S ribosomal protein S4	1	1	1	1	1	1	1	1	1	1	1	1	1	1	1	1	1	1	1	1	1	1	1	1	1	1	1	1	1	1	1	1	1	1	1	1	1	1	1	1	2
SAL0000473	group_1982	C factor cell-cell signaling protein	1	1	1	1	1	1	1	1	1	1	1	1	1	1	1	1	1	1	1	1	1	1	1	1	1	1	1	1	1	1	1	1	1	1	1	1	1	1	1	1	2
SAL0000474	ecsA	ABC-type transporter ATP-binding protein EcsA	1	1	1	2	1	1	1	1	1	1	1	1	1	1	1	1	1	1	1	1	1	1	1	1	1	1	2	1	1	1	1	1	1	1	1	1	1	1	1	1	3
SAL0000475	infB	Translation initiation factor IF-2	1	1	2	1	1	1	1	3	1	1	1	1	1	1	2	1	1	1	1	1	1	1	1	1	1	1	1	2	1	2	1	2	1	1	1	1	1	1	1	1	1
SAL0000476	ydcV	Inner membrane ABC transporter permease protein YdcV	1	1	1	1	1	1	1	1	1	1	1	3	1	1	1	1	1	1	1	1	1	1	1	1	1	1	1	1	1	1	1	1	1	1	1	1	1	1	1	1	2
SAL0000477	group_1986	FtsX-like permease family protein	1	1	1	1	1	4	1	3	1	1	1	2	1	1	1	1	1	1	1	1	1	1	1	1	1	1	1	1	1	1	1	1	1	1	1	1	1	1	1	1	5
SAL0000478	thrC	Threonine synthase	1	1	1	4	1	1	1	1	1	1	1	1	1	1	1	1	1	1	1	1	1	1	1	1	1	1	1	1	1	1	1	1	2	1	1	1	1	1	1	1	3
SAL0000479	hp	hypothetical protein	1	1	1	1	1	1	1	1	1	1	1	1	1	1	1	1	1	1	1	1	1	1	1	1	1	1	1	1	1	1	1	1	1	1	1	1	1	1	1	1	2
SAL0000480	xpkA	Xylulose-5-phosphate phosphoketolase	1	1	1	3	1	1	2	4	1	2	1	1	1	1	1	1	1	1	1	1	1	1	1	1	1	1	3	1	1	1	1	1	1	1	1	1	1	1	1	1	1
SAL0000481	hp	hypothetical protein	1	1	1	1	1	1	1	3	1	1	1	2	1	1	1	1	1	1	1	1	1	1	1	1	1	1	1	1	1	1	1	1	1	1	1	1	1	1	1	1	4
SAL0000482	czcD	Cadmium, cobalt and zinc/H( )-K( ) antiporter	1	1	2	1	1	1	1	3	1	1	1	1	1	1	2	1	1	1	1	1	1	1	1	1	1	1	1	1	1	2	1	2	1	1	1	1	1	1	1	1	4
SAL0000483	araC	Arabinose operon regulatory protein	1	1	1	2	1	1	1	1	1	1	1	1	1	1	1	1	1	1	1	1	1	1	1	1	1	1	2	1	1	1	1	1	1	1	1	1	1	1	1	1	3
SAL0000484	group_1992	YibE/F-like protein	1	1	1	2	1	1	1	1	1	1	1	1	1	1	1	1	1	1	1	1	1	1	1	1	1	1	2	1	1	1	1	1	1	1	1	1	1	1	1	1	3
SAL0000485	glyS	Glycine--tRNA ligase beta subunit	1	1	1	1	1	1	2	1	1	2	1	1	1	1	1	1	1	1	1	1	1	1	1	1	1	1	1	1	1	1	1	1	1	1	1	1	1	1	1	1	3
SAL0000486	hp	hypothetical protein	1	1	1	1	1	1	1	1	1	1	1	2	1	1	1	1	1	1	1	1	1	1	1	1	1	1	1	1	1	1	1	1	1	1	1	1	1	1	1	1	1
SAL0000487	group_1995	esterase	1	1	1	1	1	1	1	1	1	1	1	1	1	1	1	1	1	1	1	1	1	1	1	1	1	1	1	1	1	1	1	1	1	1	1	1	1	1	1	1	2
SAL0000488	alr	Alanine racemase	1	1	1	2	1	1	1	1	1	1	1	1	1	1	1	1	1	1	1	1	1	1	1	1	1	1	2	1	4	1	1	1	1	1	1	1	1	1	1	1	3
SAL0000489	mtaD	5-methylthioadenosine/S-adenosylhomocysteine deaminase	1	1	1	1	1	1	1	1	1	1	1	1	1	1	1	1	1	1	1	1	1	1	1	1	1	1	1	1	1	1	1	1	1	1	1	1	1	1	1	1	2
SAL0000490	yicL_2	putative inner membrane transporter YicL	1	1	1	1	1	1	1	3	1	1	1	1	1	1	1	1	1	1	1	1	1	1	1	1	1	1	1	1	1	1	1	1	1	1	1	1	1	1	1	1	2
SAL0000491	yhaO	putative metallophosphoesterase YhaO	1	1	2	1	1	1	1	1	1	1	1	1	1	1	1	1	1	1	1	1	1	1	1	1	1	1	1	1	1	1	1	1	1	1	1	1	1	1	1	1	3
SAL0000492	polC_2	DNA polymerase III PolC-type	1	1	2	1	1	1	1	3	1	1	1	4	1	1	1	1	1	1	1	1	1	1	1	1	1	1	1	1	1	1	1	1	1	1	1	1	1	1	1	1	1
SAL0000493	citG	2-(5''-triphosphoribosyl)-3'-dephosphocoenzyme-A synthase	1	1	1	1	1	1	1	2	1	1	1	1	1	1	1	1	1	1	1	1	1	1	1	1	1	1	1	2	1	1	1	1	1	1	1	1	1	1	1	1	3
SAL0000494	rps1	30S ribosomal protein S1	1	1	1	1	1	1	1	1	1	1	1	1	1	1	1	1	1	1	1	1	1	1	1	1	1	1	1	1	2	1	1	1	1	1	1	1	1	1	1	1	1
SAL0000495	hp	hypothetical protein	1	1	1	1	1	1	1	1	1	1	1	1	1	1	1	1	1	1	1	1	1	1	1	1	1	1	1	1	2	1	1	1	1	1	1	1	1	1	1	1	1
SAL0000496	mreB_1	Rod shape-determining protein MreB	1	1	2	1	1	1	1	1	1	1	1	1	1	1	1	1	1	1	1	1	1	1	1	1	1	1	1	3	1	1	1	1	1	1	1	1	1	1	1	1	4
SAL0000497	gmuR_3	HTH-type transcriptional regulator GmuR	1	1	2	1	1	1	1	3	1	1	1	1	1	1	2	1	1	1	1	1	1	1	1	1	1	1	1	3	1	2	1	2	1	1	1	1	1	1	1	1	4
SAL0000498	eno	Enolase	1	1	2	1	1	1	3	1	1	3	1	1	1	1	2	1	1	1	1	1	1	1	1	1	1	1	1	1	1	2	1	2	1	1	1	1	1	1	1	1	1
SAL0000499	group_2007	putative ABC transporter ATP-binding protein	1	1	1	1	1	1	1	2	1	1	1	1	1	1	1	1	1	1	1	1	1	1	1	1	1	1	1	1	1	1	1	1	1	1	1	1	1	1	1	1	1
SAL0000500	hp	hypothetical protein	1	1	1	1	1	1	1	1	1	1	1	1	1	1	1	1	1	1	1	1	1	1	1	1	1	1	1	1	1	1	1	1	1	1	1	1	1	1	1	1	2
SAL0000501	addA	ATP-dependent helicase/nuclease subunit A	4	1	1	1	1	1	1	2	1	1	1	3	1	1	1	1	1	1	1	1	1	1	1	1	1	1	1	1	1	1	1	1	1	1	1	1	1	1	1	1	5
SAL0000502	group_2010	recombinase A	1	1	2	1	1	1	1	1	1	1	1	1	1	1	1	1	1	1	1	1	1	1	1	1	1	1	1	1	1	1	1	1	1	1	1	1	1	1	1	1	3
SAL0000503	bbmA	Intracellular maltogenic amylase	1	1	1	1	1	1	1	1	1	1	1	1	1	1	1	1	1	1	1	1	1	1	1	1	1	1	1	1	1	1	1	1	1	1	1	1	1	1	1	1	2
SAL0000504	bmr3_3	Multidrug resistance protein 3	1	1	2	1	1	1	1	2	1	1	1	1	1	1	2	1	1	1	1	1	1	1	1	1	1	1	1	2	1	2	1	2	1	1	1	1	1	1	1	1	3

SAL0000505	group_2015	acyl-CoA esterase	1	1	1	1	1	1	1	1	1	1	1	1	1	1	1	1	1	1	1	1	1	1	1	1	1	1	1	1	1	1	1	1	1	1	1	1	1	1	1	1	2
SAL0000506	efp_1	Elongation factor P	1	1	1	1	1	1	2	1	1	2	1	1	1	1	1	1	1	1	1	1	1	1	1	1	1	1	1	1	1	1	1	1	1	1	1	3	1	1	1	1	1
SAL0000507	rpoA	DNA-directed RNA polymerase subunit alpha	1	1	1	1	1	3	1	2	1	1	1	1	1	1	1	1	1	1	1	1	1	1	1	1	1	1	1	1	1	1	1	1	1	1	1	1	1	1	1	1	1
SAL0000508	treR	Trehalose operon transcriptional repressor	1	1	1	1	1	1	1	2	1	1	1	1	1	1	1	1	1	1	1	1	1	1	1	1	1	1	3	1	1	1	1	1	1	1	1	1	1	1	1	1	4
SAL0000509	pyrR_2	Bifunctional protein pyrR	1	1	1	1	1	1	1	1	1	1	1	2	1	1	1	1	1	1	1	1	1	1	1	1	1	1	1	1	1	1	1	1	1	1	1	1	1	1	1	1	3
SAL0000510	ftsH	ATP-dependent zinc metalloprotease FtsH	1	1	2	7	1	8	1	6	1	1	1	4	9	1	1	1	1	1	1	1	1	1	1	1	1	1	1	1	5	1	1	1	1	1	1	1	1	1	1	1	3
SAL0000511	hp	hypothetical protein	1	1	1	2	1	1	1	1	1	1	1	1	1	1	1	1	1	1	1	1	1	1	1	1	1	1	2	1	1	1	1	1	1	1	1	1	1	1	1	1	3
SAL0000512	acpS	Holo-[acyl-carrier-protein] synthase	1	1	1	1	1	1	1	1	1	1	1	1	1	1	1	1	1	1	1	1	1	1	1	1	1	1	1	1	1	1	1	1	1	1	1	1	1	1	1	2	3
SAL0000513	gpmA_4	2,3-bisphosphoglycerate-dependent phosphoglycerate mutase	1	1	1	1	1	1	1	2	1	1	1	1	1	1	1	1	1	1	1	1	1	1	1	1	1	1	1	1	1	1	1	1	1	1	1	1	1	1	1	1	3
SAL0000514	gspK	Glucosamine kinase GspK	1	1	1	1	1	1	1	1	1	1	1	1	1	1	1	1	1	1	1	1	1	1	1	1	1	1	1	1	1	1	1	1	1	1	1	1	1	1	1	1	2
SAL0000515	artM_1	Arginine transport ATP-binding protein ArtM	1	1	1	4	1	1	2	1	1	2	1	1	1	1	1	1	1	1	1	1	1	1	1	1	1	1	1	1	1	1	1	1	1	1	1	1	1	1	1	1	3
SAL0000516	group_2026	Helix-turn-helix domain protein	1	1	3	1	1	1	1	2	1	1	1	1	1	1	1	1	1	1	1	1	1	1	1	1	1	1	1	1	1	1	1	1	1	1	1	1	1	1	1	1	1
SAL0000517	iphP_2	Tyrosine-protein phosphatase precursor	1	1	1	1	1	1	1	1	1	1	1	1	1	1	1	1	1	1	1	1	1	1	1	1	1	1	1	1	1	1	1	1	1	1	1	1	1	1	1	1	2
SAL0000518	iscS_2	Cysteine desulfurase	1	1	1	1	1	1	2	1	1	2	1	1	1	1	1	1	1	1	1	1	1	1	1	1	1	1	1	1	1	1	1	1	1	1	1	1	1	1	1	1	3
SAL0000519	group_2029	Fumarate reductase flavoprotein subunit precursor	1	1	1	1	1	2	1	3	1	1	1	1	1	1	1	1	1	1	1	1	1	1	1	1	1	1	1	1	1	1	1	1	1	1	1	1	1	1	1	1	4
SAL0000520	dagK	Diacylglycerol kinase	1	1	1	1	1	2	1	1	1	1	1	1	1	1	1	1	1	1	1	1	1	1	1	1	1	1	1	1	1	1	1	1	1	1	1	1	1	1	1	1	1
SAL0000521	pgk	Phosphoglycerate kinase	1	1	1	1	1	1	1	2	1	1	1	1	1	1	1	1	1	1	1	1	1	1	1	1	1	1	1	1	1	1	1	1	1	1	1	1	1	1	1	1	1
SAL0000522	hp	hypothetical protein	1	1	1	1	1	1	1	1	1	1	1	1	1	1	1	1	1	1	1	1	1	1	1	1	1	1	1	1	1	1	1	1	1	1	1	1	1	1	1	1	2
SAL0000523	group_2033	flavodoxin	1	1	1	1	1	1	1	2	1	1	1	1	1	1	1	1	1	1	1	1	1	1	1	1	1	1	1	1	1	1	1	1	1	1	1	1	1	1	1	1	1
SAL0000524	sph	Oleate hydratase	1	1	2	1	1	1	1	4	1	1	1	3	1	1	2	1	1	1	1	1	1	1	1	1	1	1	1	1	1	2	1	2	1	1	1	1	1	1	1	1	5
SAL0000525	uvrB	UvrABC system protein B	1	1	1	1	1	1	1	3	1	1	1	1	1	1	4	1	1	1	1	1	1	1	1	1	1	1	1	1	5	1	1	1	1	1	1	1	1	1	1	1	2
SAL0000526	group_2036	Bacterial transcription activator, effector binding domain	1	1	1	1	1	1	1	1	1	1	1	1	1	1	1	1	1	1	1	1	1	1	1	1	1	1	1	1	1	1	1	1	1	1	1	1	1	1	1	1	2
SAL0000527	group_2037	GIY-YIG nuclease superfamily protein	1	1	1	1	1	2	1	1	1	1	1	3	1	1	1	1	1	1	1	1	1	1	1	1	1	1	1	1	1	1	1	1	1	1	1	1	1	1	1	1	1
**SAL0000528**	**secY**	**preprotein translocase subunit secY**	1	1	1	1	1	1	1	3	1	2	1	1	1	1	1	1	1	1	1	1	1	1	1	1	1	1	1	1	1	1	1	1	1	1	1	1	1	1	1	1	1
SAL0000529	zwf	Glucose-6-phosphate 1-dehydrogenase	1	1	3	1	1	1	2	4	1	2	1	1	1	1	1	1	1	1	1	1	1	1	1	1	1	1	1	1	1	1	1	1	1	1	1	1	1	1	1	1	5
SAL0000530	disA	DNA integrity scanning protein DisA	1	1	1	1	1	1	1	2	1	1	1	1	1	1	1	1	1	1	1	1	1	1	1	1	1	1	1	1	1	1	1	1	1	1	1	1	1	1	1	1	1
SAL0000531	bglH_3	Aryl-phospho-beta-D-glucosidase BglH	1	1	1	1	1	1	1	2	1	1	1	1	1	1	1	1	1	1	1	1	1	1	1	1	1	1	1	2	1	1	1	1	1	1	1	1	1	1	1	1	3
**SAL0000532**	**uvrA_1**	**UvrABC system protein A**	1	1	2	1	1	1	1	5	1	3	1	1	1	1	2	1	1	1	1	1	1	1	1	1	1	1	1	1	1	2	1	2	1	1	1	1	1	1	1	1	4
SAL0000533	group_2043	autoinducer-2 (AI-2) modifying protein LsrG	1	1	1	1	1	1	1	1	1	1	1	1	1	1	1	1	1	1	1	1	1	1	1	1	1	1	1	1	1	1	1	1	1	1	1	1	1	1	1	1	2
SAL0000534	group_2044	Peptidase propeptide and YPEB domain protein	1	1	1	1	1	1	1	3	1	1	1	1	1	1	1	1	1	1	1	1	1	1	1	1	1	1	1	1	1	1	1	1	1	1	1	1	1	1	1	1	2
SAL0000535	group_2045	Sugar phosphatase YidA	1	1	1	1	1	1	1	1	1	1	1	1	1	1	1	1	1	1	1	1	1	1	1	1	1	1	1	1	3	1	1	1	1	1	1	1	1	1	1	1	2
SAL0000536	group_2046	ABC-2 family transporter protein	1	1	1	2	1	1	1	1	1	1	1	1	1	1	1	1	1	1	1	1	1	1	1	1	1	1	2	1	1	1	1	1	1	1	1	1	1	1	1	1	3
SAL0000537	dxs_1	1-deoxy-D-xylulose-5-phosphate synthase	1	1	3	1	1	1	1	1	1	1	1	1	1	1	1	1	1	1	1	1	1	1	1	1	1	1	1	1	1	1	1	1	1	1	1	1	1	1	1	1	2
SAL0000538	prmA	Ribosomal protein L11 methyltransferase	1	1	1	2	1	1	1	1	1	1	1	1	1	1	1	1	1	1	1	1	1	1	1	1	1	1	2	1	1	1	1	1	1	1	1	1	1	1	1	1	3
SAL0000539	ychF	Ribosome-binding ATPase YchF	1	1	1	1	1	1	1	2	1	1	1	1	1	1	1	1	1	1	1	1	1	1	1	1	1	1	1	1	1	1	1	1	1	1	1	1	1	1	1	1	3
SAL0000540	cpdB	2',3'-cyclic-nucleotide 2'-phosphodiesterase/3'-nucleotidase precursor	1	1	2	1	1	1	1	1	1	1	1	1	1	1	1	1	1	1	1	1	1	1	1	1	1	1	1	1	1	1	1	1	1	1	1	1	1	1	1	1	3
**SAL0000541**	**tr**	**Transcriptional regulator**	1	1	1	1	1	1	1	1	2	1	1	1	1	1	1	2	2	2	2	1	1	2	2	2	2	2	1	1	1	1	1	1	1	3	2	1	3	2	2	1	4
SAL0000542	purH	Bifunctional purine biosynthesis protein PurH	1	1	1	1	1	1	1	1	1	1	1	1	1	1	1	1	1	1	1	1	1	1	1	1	1	1	1	1	1	1	1	1	1	1	1	1	1	1	1	1	2
SAL0000543	hslO	33 kDa chaperonin	1	1	1	1	1	1	1	1	1	1	1	1	1	1	1	1	1	1	1	1	1	1	1	1	1	1	1	1	1	1	1	1	1	1	1	1	1	1	1	1	2
SAL0000544	yifK_1	putative transport protein YifK	1	1	1	1	1	1	2	1	1	2	1	1	1	1	1	1	1	1	1	1	1	1	1	1	1	1	1	1	1	1	1	1	1	1	1	1	1	1	1	1	1
SAL0000545	hp	hypothetical protein	1	1	1	1	1	1	1	3	1	1	1	1	1	1	1	1	1	1	1	1	1	1	1	1	1	1	1	1	1	1	1	1	1	1	1	1	1	1	1	1	2
SAL0000546	tsf	Elongation factor Ts	1	1	1	1	1	1	1	1	1	1	1	1	1	1	1	1	1	1	1	1	1	1	1	1	1	1	1	1	1	1	1	1	1	1	1	1	1	1	1	1	2
SAL0000547	tcrY	putative sensor histidine kinase TcrY	1	1	1	2	1	1	3	1	1	3	1	1	1	1	1	1	1	1	1	1	1	1	1	1	1	1	2	1	1	1	1	1	1	1	1	1	1	1	1	1	4
SAL0000548	iscS_1	Cysteine desulfurase	1	1	1	1	1	1	1	1	1	1	1	1	1	1	1	1	1	1	1	1	1	1	1	3	1	1	1	1	1	1	1	1	1	1	1	1	1	1	1	1	2
SAL0000549	rnz	Ribonuclease Z	1	1	1	1	1	1	1	1	1	1	1	1	1	1	1	1	1	1	1	1	1	1	1	1	1	1	1	1	1	2	1	2	1	1	1	1	1	1	1	1	1
SAL0000550	rbfA	Ribosome-binding factor A	1	1	1	1	1	1	1	1	1	1	1	1	1	1	1	1	1	1	1	1	1	1	1	1	1	1	1	1	1	1	1	1	1	1	1	1	1	1	1	1	2
SAL0000551	zosA_1	Zinc-transporting ATPase	1	1	1	2	1	1	1	3	1	1	1	1	1	1	1	1	1	1	1	1	1	1	1	1	1	1	2	1	1	1	1	1	1	1	1	1	1	1	1	1	4
SAL0000552	upp	Uracil phosphoribosyltransferase	1	1	1	1	1	1	2	1	1	2	1	1	1	1	1	1	1	1	1	1	1	1	1	1	1	1	1	1	1	1	1	1	1	1	1	1	1	1	1	1	1
SAL0000553	hp	hypothetical protein	1	1	1	1	1	1	1	1	1	1	1	1	1	1	1	1	1	1	1	1	1	1	1	1	1	1	1	1	1	1	1	1	1	1	1	1	1	1	1	1	2
SAL0000554	rafA	Alpha-galactosidase	1	1	2	1	1	1	1	2	1	1	1	1	1	1	2	1	1	1	1	1	1	1	1	1	1	1	1	2	1	2	1	2	1	1	1	1	1	1	1	1	3
SAL0000555	galE	UDP-glucose 4-epimerase	1	1	2	1	1	1	1	4	1	1	1	1	1	1	2	1	1	1	1	1	1	1	1	1	1	1	1	1	1	2	1	2	1	1	1	1	1	1	1	1	3
SAL0000556	dapB	4-hydroxy-tetrahydrodipicolinate reductase	1	1	1	1	1	1	1	2	1	1	1	1	1	1	1	1	1	1	1	1	1	1	1	1	1	1	1	2	1	1	1	1	1	1	1	1	1	1	1	1	3
SAL0000557	glmM	Phosphoglucosamine mutase	1	1	1	1	1	1	1	1	1	1	1	1	1	1	1	1	1	1	1	1	1	1	1	1	1	1	1	1	1	1	1	1	1	1	1	1	1	1	1	1	2
SAL0000558	atpA	ATP synthase subunit alpha	1	1	2	2	1	2	2	2	1	2	1	2	1	1	2	1	1	1	1	1	1	1	1	1	1	1	2	2	2	2	1	2	1	1	1	1	1	1	1	1	3
SAL0000559	group_2077	Nitroreductase family protein	1	1	1	2	1	4	1	1	1	1	1	1	1	1	1	1	1	1	1	1	1	1	1	1	1	1	2	1	1	1	1	1	1	1	1	1	1	1	1	1	3
SAL0000560	pcrA	ATP-dependent DNA helicase PcrA	1	1	1	1	1	1	1	1	1	1	1	1	1	1	1	1	1	1	1	1	1	1	1	1	1	1	1	1	1	1	1	1	1	1	1	1	1	1	1	1	2
SAL0000561	sad	Succinate semialdehyde dehydrogenase [NAD(P)] Sad	1	1	1	1	1	1	1	1	1	1	1	2	1	1	1	1	1	1	1	1	1	1	1	1	1	1	1	1	1	1	1	1	1	1	1	1	1	1	1	1	3
SAL0000562	rarA_2	Replication-associated recombination protein A	1	1	1	1	1	1	1	1	1	1	1	1	1	1	1	1	1	1	1	1	1	1	1	1	1	1	1	1	2	1	1	1	1	1	1	1	1	1	1	1	3
SAL0000563	azr	NADPH azoreductase	1	1	1	1	1	1	1	2	1	1	1	1	1	1	1	1	1	1	1	1	1	1	1	1	1	1	1	2	3	1	1	1	1	1	1	1	1	1	1	1	4
SAL0000564	group_2082	putative HTH-type transcriptional regulator	1	1	1	1	1	1	1	1	1	1	1	1	1	1	1	1	1	1	1	1	1	1	1	1	1	1	1	1	1	1	1	1	1	1	1	1	1	1	1	1	2
SAL0000565	truA	tRNA pseudouridine synthase A	1	1	1	1	1	1	1	1	1	1	1	1	1	1	1	1	1	1	1	1	1	1	1	1	1	1	1	1	1	1	1	1	1	1	1	1	1	1	1	1	2
SAL0000566	group_2084	putative hydrolase	1	1	1	1	1	1	1	2	1	1	1	4	1	1	1	1	1	1	1	1	1	3	1	1	1	1	1	1	1	1	1	1	1	1	1	5	1	1	1	1	6
SAL0000567	gatC	Glutamyl-tRNA(Gln) amidotransferase subunit C	1	1	1	1	1	1	1	1	1	1	1	1	1	1	1	1	1	1	1	1	1	1	1	1	1	1	1	1	1	1	1	1	1	1	1	1	1	1	1	1	2
SAL0000568	glnA	Glutamine synthetase	1	1	1	1	1	1	1	1	1	1	1	1	1	1	1	1	1	1	1	1	1	1	1	1	1	1	1	1	1	1	1	1	1	1	1	1	1	1	1	1	2

SAL0000569	dppE_1	Dipeptide-binding protein DppE precursor	1	1	1	1	1	1	1	1	1	1	1	1	1	1	1	1	1	1	1	1	1	1	1	1	1	1	1	1	1	1	1	1	1	1	1	1	1	1	1	1	2
SAL0000570	group_2088	Hemerythrin HHE cation binding domain protein	1	1	1	1	1	1	1	1	1	1	1	1	1	1	1	1	1	1	1	1	1	1	1	1	1	1	1	1	1	1	1	1	1	1	1	1	1	1	1	1	2
SAL0000571	rsfS	Ribosomal silencing factor RsfS	1	1	1	1	1	1	1	1	1	1	1	1	1	1	1	1	1	1	1	1	1	1	1	1	1	1	1	1	1	1	1	1	1	1	1	1	1	1	1	1	2
SAL0000572	bca_2	C protein alpha-antigen precursor	1	1	2	1	1	1	1	7	1	1	1	5	1	1	2	1	1	3	3	1	1	1	6	1	1	1	1	1	1	2	1	2	1	1	1	1	1	1	1	1	4
SAL0000573	group_2090	putative oxidoreductase	1	1	1	1	1	2	1	1	1	1	1	1	1	1	1	1	1	1	1	1	1	1	1	1	1	1	1	1	1	1	1	1	1	1	1	1	1	1	1	1	1
SAL0000574	group_2091	EAL domain protein	1	1	1	1	1	1	1	2	1	1	1	1	1	1	1	1	1	1	1	1	1	1	1	1	1	1	1	1	1	1	1	1	1	1	1	1	1	1	1	1	3
SAL0000575	glcA	Glycolate permease GlcA	1	1	1	1	1	1	2	1	1	2	1	1	1	1	1	1	1	1	1	1	1	1	1	1	1	1	1	1	1	1	1	1	1	1	1	1	1	1	1	1	3
SAL0000576	cobC	Alpha-ribazole phosphatase	1	1	1	1	1	1	1	1	1	1	1	1	1	1	1	1	1	1	1	1	1	1	1	1	1	1	1	1	1	1	1	1	1	1	1	1	1	1	1	1	2
SAL0000577	group_2094	YibE/F-like protein	1	1	1	1	1	1	1	1	1	1	1	1	1	1	1	1	1	1	1	1	1	1	1	1	1	1	1	1	1	1	1	1	1	1	1	1	1	1	1	1	2
SAL0000578	hp	hypothetical protein	1	1	1	1	1	1	1	1	1	1	1	1	1	1	1	1	1	1	1	1	1	1	1	1	1	1	1	1	2	1	1	1	1	1	1	1	1	1	1	1	3
SAL0000579	frc_2	Formyl-coenzyme A transferase	1	1	1	1	1	1	1	1	1	1	1	1	1	1	1	1	1	1	1	1	1	1	1	1	1	1	1	1	1	1	1	1	1	1	1	1	1	1	1	1	1
SAL0000580	rpsL	30S ribosomal protein S12	1	1	1	1	1	2	1	1	1	1	1	1	1	1	1	1	1	1	1	1	1	1	1	1	1	1	1	1	1	1	1	1	1	1	1	1	1	1	1	1	1
SAL0000581	typA	GTP-binding protein TypA/BipA	1	1	1	4	1	2	1	1	1	1	1	3	1	1	1	1	1	1	1	1	1	1	1	1	1	1	1	1	1	1	1	1	1	1	1	1	1	1	1	1	1
SAL0000582	group_2100	ComEC family competence protein	1	1	1	1	1	1	2	3	1	2	1	1	1	1	1	1	1	1	1	1	1	1	1	1	1	1	1	1	1	1	1	1	1	1	1	1	1	1	1	1	1
SAL0000583	tuf	Elongation factor Tu	1	1	2	1	1	1	1	1	1	1	1	1	1	1	2	1	1	1	1	1	1	1	1	1	1	1	1	1	1	2	1	2	1	1	1	1	1	1	1	1	1
SAL0000584	mgs	Alpha-monoglucosyldiacylglycerol synthase	1	1	1	1	1	1	1	2	1	1	1	1	1	1	1	1	1	1	1	1	1	1	1	1	1	1	1	1	4	1	1	1	1	1	1	1	1	1	1	1	3
SAL0000585	group_2103	putative ABC transporter ATP-binding protein	1	1	1	1	1	1	1	2	1	1	1	3	1	1	1	1	1	1	1	1	1	1	1	1	1	1	1	2	1	1	1	1	1	1	1	1	1	1	1	1	1
SAL0000586	ytpP	Thioredoxin-like protein YtpP	1	1	1	1	1	1	1	1	1	1	1	1	1	1	1	1	1	1	1	1	1	1	1	1	1	1	1	1	1	1	1	1	1	1	1	2	1	1	1	1	3
SAL0000587	pth	Peptidyl-tRNA hydrolase	1	1	1	1	1	1	1	1	1	1	1	1	1	1	1	1	1	1	1	1	1	1	1	1	1	1	1	1	1	1	1	1	1	1	1	1	1	1	1	1	2
SAL0000588	fabG	3-oxoacyl-[acyl-carrier-protein] reductase FabG	1	1	1	1	1	1	1	1	1	1	1	1	1	1	1	1	1	1	1	1	1	1	1	1	1	1	1	1	1	1	1	1	1	1	1	1	1	1	1	1	2
SAL0000589	cmk	Cytidylate kinase	1	1	1	1	1	2	1	1	1	1	1	1	1	1	1	1	1	1	1	1	1	1	1	1	1	1	1	1	1	1	1	1	1	1	1	1	1	1	1	1	1
SAL0000590	garK	Glycerate 2-kinase	1	1	3	1	1	1	1	1	1	1	1	1	1	1	1	1	1	1	1	1	1	1	1	1	1	1	1	1	2	1	1	1	1	1	1	1	1	1	1	1	4
SAL0000591	ybbL	putative ABC transporter ATP-binding protein YbbL	1	1	1	1	1	1	1	1	1	1	1	1	1	1	1	1	1	1	1	1	1	1	1	1	1	1	1	1	1	1	1	1	1	1	1	1	1	1	1	1	2
SAL0000592	hp	hypothetical protein	1	1	1	1	1	1	1	1	1	1	1	1	1	1	1	1	1	1	1	1	1	1	1	1	1	1	1	1	1	1	1	1	1	1	1	1	1	1	1	1	2
SAL0000593	yfbR	5'-deoxynucleotidase YfbR	1	1	1	1	1	1	1	1	1	1	1	2	1	1	1	1	1	1	1	1	1	1	1	1	1	1	1	1	3	1	1	1	1	1	1	1	1	1	1	1	4
SAL0000594	epsF	Putative glycosyltransferase EpsF	1	1	1	1	1	1	1	1	1	1	1	1	1	1	1	1	1	1	1	1	1	1	1	1	1	1	1	1	1	1	1	1	1	1	1	1	1	1	1	1	2
SAL0000595	dppE_4	Dipeptide-binding protein DppE precursor	1	1	3	1	1	1	4	5	1	4	1	1	1	1	2	1	1	1	1	1	1	1	1	1	1	1	1	3	1	2	1	2	1	1	1	1	1	1	1	1	6
SAL0000596	yjeM	Inner membrane transporter YjeM	1	1	1	1	1	1	1	1	1	1	1	1	1	1	1	1	1	1	1	1	1	1	1	1	1	1	1	1	1	1	1	1	1	1	1	1	1	1	1	1	2
SAL0000597	topA	DNA topoisomerase 1	1	1	1	1	1	1	1	2	1	1	1	1	1	1	1	1	1	1	1	1	1	1	1	1	1	1	1	1	1	1	1	1	1	1	1	1	1	1	1	1	3
SAL0000598	hp	hypothetical protein	1	1	1	1	1	1	1	1	1	1	1	1	1	1	1	1	1	1	1	1	1	1	1	1	1	1	1	1	1	1	1	1	1	1	1	2	1	1	1	1	1
SAL0000599	trxB_1	Thioredoxin reductase	1	1	1	1	1	1	1	1	1	1	1	1	1	1	1	1	1	1	1	1	1	1	1	1	1	1	1	1	1	1	1	1	1	1	1	1	1	1	1	1	2
SAL0000600	hp	hypothetical protein	1	1	1	1	1	4	1	1	1	1	1	3	1	1	1	1	1	1	1	1	1	1	1	1	1	1	1	1	1	1	1	1	1	1	1	1	1	1	1	1	2
SAL0000601	slpA_1	S-layer protein precursor	1	1	1	2	1	1	1	1	1	1	1	3	1	1	1	1	1	1	1	1	1	1	1	1	1	1	1	1	1	1	1	1	1	1	1	1	1	1	1	1	4
SAL0000602	rutD	Putative aminoacrylate hydrolase RutD	1	1	2	1	1	1	1	2	1	1	1	1	1	1	2	1	1	1	1	1	1	1	1	1	1	1	1	2	1	2	1	2	1	1	1	1	1	1	1	1	3
SAL0000603	bglK_2	Beta-glucoside kinase	1	1	1	1	1	1	1	3	1	1	1	2	1	1	1	1	1	1	1	1	1	1	1	1	1	1	1	1	1	1	1	1	1	1	1	1	1	1	1	1	4
SAL0000604	oppF_2	Oligopeptide transport ATP-binding protein OppF	1	1	1	1	1	1	1	1	1	1	1	1	2	1	1	1	1	1	1	1	1	1	1	1	1	1	1	1	1	1	1	1	1	1	1	1	1	1	1	1	1
SAL0000605	hp	hypothetical protein	1	1	1	1	1	1	1	1	1	1	1	1	1	1	1	1	1	1	1	1	1	1	1	1	1	1	1	1	1	1	1	1	3	1	1	1	1	1	1	1	2
SAL0000606	rssA	NTE family protein RssA	1	1	1	1	1	3	1	4	1	1	1	1	1	1	2	1	1	1	1	1	1	1	1	1	1	1	1	1	1	2	1	2	1	1	1	1	1	1	1	1	1
SAL0000607	endA_1	DNA-entry nuclease	1	1	1	1	1	1	1	1	1	1	1	1	1	1	1	1	1	1	1	1	1	1	1	1	1	1	1	1	1	1	1	1	1	1	1	1	1	1	1	1	2
**SAL0000608**	**tilS**	**tRNA(Ile)-lysidine synthase**	1	1	1	1	1	1	1	1	1	3	1	1	1	1	1	1	1	1	1	1	1	1	1	1	1	1	1	1	1	1	1	1	1	1	1	1	1	1	1	1	2
SAL0000609	dusC	tRNA-dihydrouridine synthase C	1	1	1	1	1	1	1	1	1	1	1	1	1	1	1	1	1	1	1	1	1	1	1	1	1	1	1	1	1	1	1	1	1	1	1	1	1	1	1	1	2
SAL0000610	atpG	ATP synthase gamma chain	1	1	1	1	1	1	1	1	1	1	1	1	1	1	1	1	1	1	1	1	1	1	1	1	1	1	1	1	1	1	1	1	1	1	1	1	1	1	1	1	2
SAL0000611	purR_1	Pur operon repressor	1	1	1	1	1	1	1	2	1	1	1	1	1	1	1	1	1	1	1	1	1	1	1	1	1	1	1	2	1	1	1	1	1	1	1	1	1	1	1	1	3
SAL0000612	hp	hypothetical protein	1	1	1	1	1	1	1	1	1	1	1	3	1	1	1	1	1	1	1	1	1	1	1	1	1	1	1	1	1	1	1	1	1	1	1	1	1	1	1	1	2
SAL0000613	pgl	6-phosphogluconolactonase	1	1	1	1	1	1	1	2	1	1	1	1	1	1	1	1	1	1	1	1	1	1	1	1	1	1	1	1	1	1	1	1	1	1	1	1	1	1	1	1	3
SAL0000614	nrdR	Transcriptional repressor NrdR	1	1	1	1	1	1	1	1	1	1	1	1	1	1	1	1	1	1	1	1	1	1	1	1	1	1	1	1	3	1	1	1	1	1	1	1	1	1	1	1	2
SAL0000615	group_2133	phosphatidylserine decarboxylase	1	1	1	1	1	1	1	3	1	1	1	1	1	1	1	1	1	1	1	1	1	1	1	1	1	1	1	1	1	1	1	1	1	1	1	1	1	1	1	1	2
SAL0000616	mvaA	3-hydroxy-3-methylglutaryl-coenzyme A reductase	1	1	1	2	1	1	1	1	1	1	1	1	1	1	1	1	1	1	1	1	1	1	1	1	1	1	2	1	1	1	1	1	1	1	1	1	1	1	1	1	1
SAL0000617	group_2135	Amidohydrolase	1	1	1	1	1	1	1	2	1	1	1	3	1	1	1	1	1	1	1	1	1	1	1	1	1	1	1	2	1	1	1	1	1	1	1	1	1	1	1	1	1
SAL0000618	murD	UDP-N-acetylmuramoylalanine--D-glutamate ligase	1	1	1	1	1	1	1	1	1	1	1	1	1	1	1	1	1	1	1	1	1	1	1	1	1	1	2	1	1	1	1	1	1	1	1	1	1	1	1	1	3
SAL0000619	cdsA	Phosphatidate cytidylyltransferase	1	1	1	1	1	1	1	4	1	1	1	2	1	1	1	1	1	1	1	1	1	1	1	1	1	1	1	1	1	1	1	1	3	1	1	1	1	1	1	1	1
SAL0000620	rpsE	30S ribosomal protein S5	1	1	1	1	1	1	1	1	1	1	1	1	1	1	1	1	1	1	1	1	1	1	1	1	1	1	1	1	2	1	1	1	1	1	1	1	1	1	1	1	1
SAL0000621	hp	hypothetical protein	1	1	1	1	1	1	1	2	1	1	1	4	1	1	1	1	1	1	1	1	1	1	1	1	1	1	1	2	1	1	1	1	1	1	1	1	1	1	1	1	3
SAL0000622	apbE	Thiamine biosynthesis lipoprotein ApbE precursor	1	1	1	1	1	1	1	1	1	1	1	1	1	1	1	1	1	1	1	1	1	1	1	1	1	1	1	1	1	1	1	1	1	1	1	1	1	1	1	1	2
SAL0000623	yjjP_2	Inner membrane protein YjjP	1	1	2	3	1	1	1	2	1	1	1	1	1	1	2	1	1	1	1	1	1	1	1	1	1	1	3	2	1	2	1	2	1	1	1	1	1	1	1	1	4
SAL0000624	pyrG	CTP synthase	1	1	3	1	1	1	1	1	1	1	1	4	1	1	1	1	1	1	1	1	1	1	1	1	1	1	1	1	1	1	1	1	1	1	1	1	1	1	1	1	2
SAL0000625	rny	Ribonuclease Y	1	1	2	2	1	4	2	3	1	2	1	2	1	1	2	1	1	1	1	1	1	1	1	1	1	1	2	3	2	2	1	2	1	1	1	1	1	1	1	1	2
SAL0000626	group_2144	acid-resistance membrane protein	1	1	1	1	1	1	1	1	1	2	1	1	1	1	1	1	1	1	1	1	1	1	1	1	1	1	1	1	1	1	1	1	1	1	1	1	1	1	1	1	1
SAL0000627	hp	hypothetical protein	1	1	1	1	1	1	1	1	1	1	1	1	1	1	1	1	1	1	1	1	1	1	1	1	1	1	2	1	1	1	1	1	1	1	1	1	1	1	1	1	1
SAL0000628	oxc	Oxalyl-CoA decarboxylase	1	1	1	1	1	3	1	1	1	1	1	1	1	1	1	1	1	1	1	1	1	1	1	1	1	1	1	1	1	1	1	1	1	1	1	1	1	1	1	1	2
SAL0000629	hp	hypothetical protein	1	1	1	1	1	1	1	1	1	1	1	1	1	1	1	1	1	1	1	1	1	1	1	1	1	1	1	1	1	1	1	1	1	1	1	1	1	1	1	1	2
SAL0000630	nagAa	Ferredoxin--NAD(P)( ) reductase (naphthalene dioxygenase/salicylate 5-hydro	1	1	1	1	1	1	1	1	1	1	1	1	1	1	1	1	1	1	1	1	1	1	1	1	1	1	1	1	1	1	1	1	1	1	1	1	1	1	1	1	2
SAL0000631	hp	hypothetical protein	1	1	1	1	1	1	1	4	1	1	1	2	1	1	1	1	1	1	1	1	1	1	1	1	1	1	1	1	1	1	1	1	1	1	1	1	1	1	1	1	3
SAL0000632	gluP	Rhomboid protease GluP	1	1	1	1	1	1	1	1	1	1	1	2	1	1	1	1	1	1	1	1	1	1	1	1	1	1	1	1	3	1	1	1	1	1	1	1	1	1	1	1	1

SAL0000633	hp	hypothetical protein	1	1	1	1	1	1	1	1	1	1	1	1	1	1	1	1	1	1	1	1	1	1	1	1	1	1	1	1	1	1	1	1	1	1	1	1	1	1	1	1	2
SAL0000634	ezrA	Septation ring formation regulator EzrA	1	1	1	1	1	1	1	2	1	1	1	1	1	1	1	1	1	1	1	1	1	1	1	1	1	1	1	2	1	1	1	1	1	1	1	3	1	1	1	1	1
SAL0000635	mraY	Phospho-N-acetylmuramoyl-pentapeptide-transferase	1	1	1	1	1	3	1	2	1	1	1	1	1	1	1	1	1	1	1	1	1	1	1	1	1	1	1	2	1	1	1	1	1	1	1	1	1	1	1	1	4
SAL0000636	cca	CCA-adding enzyme	1	1	2	1	1	1	1	4	1	1	1	1	1	1	2	1	1	1	1	1	1	1	1	1	1	1	1	1	1	2	1	2	1	1	1	1	1	1	1	1	3
SAL0000637	ponA	Penicillin-binding protein 1A	1	1	1	1	1	1	1	1	1	1	1	1	1	1	1	1	1	1	1	1	1	1	1	1	1	1	1	1	2	1	1	1	1	1	1	3	1	1	1	1	4
SAL0000638	group_2157	putative hydrolase	1	1	1	1	1	1	1	1	1	1	1	2	1	1	1	1	1	1	1	1	1	1	1	1	1	1	1	1	1	1	1	1	1	1	1	1	1	1	1	1	1
SAL0000639	gmk_1	Guanylate kinase	1	1	1	1	1	1	2	1	1	2	1	1	1	1	1	1	1	1	1	1	1	1	1	1	1	1	1	1	1	1	1	1	1	1	1	1	1	1	1	1	3
SAL0000640	group_2159	Peptidase M16 inactive domain protein	1	1	1	1	1	1	1	1	1	1	1	1	1	1	1	1	1	1	1	1	1	1	1	1	1	1	1	1	1	2	1	2	1	1	1	1	1	1	1	1	1
SAL0000641	hp	hypothetical protein	1	1	1	1	1	1	1	1	1	1	1	1	1	1	1	1	1	1	1	1	1	1	1	1	1	1	1	1	1	1	1	1	1	1	1	1	1	1	1	1	1
SAL0000642	oppF_1	Oligopeptide transport ATP-binding protein OppF	1	1	1	1	1	1	1	1	1	1	1	1	1	1	1	1	1	1	1	1	1	1	1	1	1	1	1	1	2	1	1	1	1	1	1	1	1	1	1	1	1
SAL0000643	ung	Uracil-DNA glycosylase	1	1	1	1	1	1	2	1	1	2	1	1	1	1	1	1	1	1	1	1	1	1	1	1	1	1	1	1	1	1	1	1	1	1	1	1	1	1	1	1	1
SAL0000644	trmFO	Methylenetetrahydrofolate--tRNA-(uracil-5-)-methyltransferase TrmFO	1	1	1	2	1	1	1	1	1	1	1	1	1	1	1	1	1	1	1	1	1	1	1	1	1	1	2	1	1	1	1	1	3	1	1	1	1	1	1	1	4
SAL0000645	group_2165	OsmC-like protein	1	1	1	1	1	1	1	3	1	1	1	1	1	1	1	1	1	1	1	1	1	1	1	1	1	1	1	1	1	1	1	1	1	1	1	1	1	1	1	1	2
SAL0000646	malG	Maltose transport system permease protein MalG	1	1	1	1	1	1	2	1	1	2	1	1	1	1	1	1	1	1	1	1	1	1	1	1	1	1	1	1	1	1	1	1	1	1	1	1	1	1	1	1	3
SAL0000647	group_2167	Calcium-transporting ATPase 1	1	1	1	1	1	1	1	3	1	1	1	1	1	1	1	1	1	1	1	1	1	1	1	1	1	1	1	1	1	1	1	1	1	1	1	1	1	1	1	1	2
SAL0000648	group_2168	putative multidrug resistance ABC transporter ATP-binding/permease protein	1	1	1	1	1	1	1	2	1	1	1	1	1	1	1	1	1	1	1	1	1	1	1	1	1	1	1	2	1	1	1	1	1	1	1	1	1	1	1	1	3
SAL0000649	dhfR	Dihydrofolate reductase	1	1	2	1	1	1	1	1	1	1	1	1	1	1	2	1	1	1	1	1	1	1	1	1	1	1	1	1	1	2	1	2	1	1	1	1	1	1	1	1	1
SAL0000650	ddlA	D-alanine--D-alanine ligase A	1	1	1	1	1	1	1	1	1	3	1	1	1	1	1	1	1	1	1	1	1	1	1	1	1	1	1	1	1	1	1	1	1	1	1	1	1	1	1	1	2
SAL0000651	lytG_2	Exo-glucosaminidase LytG precursor	1	1	1	1	1	1	1	1	1	1	1	1	1	1	2	1	1	1	1	1	1	1	1	1	1	1	1	1	1	1	1	1	1	1	1	1	1	1	1	1	3
SAL0000652	dnaB	Replication initiation and membrane attachment protein	1	1	1	1	1	1	3	2	1	3	1	1	1	1	1	1	1	5	1	1	1	1	1	1	1	1	1	2	1	1	1	1	1	1	1	1	1	1	1	1	4
SAL0000653	hp	hypothetical protein	1	1	1	1	1	1	1	1	1	1	1	1	1	1	1	1	1	1	1	1	1	1	1	1	1	1	1	1	1	1	1	1	1	1	1	1	1	1	1	1	2
SAL0000654	ybjI_2	Flavin mononucleotide phosphatase YbjI	1	1	2	1	1	1	1	3	1	1	1	1	1	1	2	1	1	1	1	1	1	1	1	1	1	1	1	1	1	2	1	2	1	1	1	1	1	1	1	1	1
SAL0000655	murE	UDP-N-acetylmuramoyl-L-alanyl-D-glutamate--L-lysine ligase	1	1	1	1	1	1	1	1	1	1	1	1	1	1	1	1	1	1	1	1	1	1	1	1	1	1	1	1	1	1	1	1	1	1	1	1	1	1	1	1	2
SAL0000656	albF	Putative zinc protease AlbF	1	1	2	1	1	1	1	5	1	1	1	1	1	1	2	1	1	1	1	1	1	1	1	1	1	1	1	1	3	2	1	2	1	1	1	1	1	1	1	1	4
SAL0000657	rlmL	Ribosomal RNA large subunit methyltransferase L	1	1	3	1	1	1	1	1	1	1	1	1	1	1	1	1	1	1	1	1	1	1	1	1	1	1	1	1	1	1	1	1	1	1	1	1	1	1	1	1	2
SAL0000658	rnr	Ribonuclease R	1	1	1	1	1	1	1	2	1	1	1	1	1	1	1	1	1	1	1	1	1	1	1	1	1	1	1	1	1	1	1	1	1	1	1	1	1	1	1	1	1
SAL0000659	hp	hypothetical protein	1	1	1	1	1	1	1	1	1	1	1	1	1	1	1	1	1	1	1	1	1	1	1	1	1	1	1	1	1	2	1	2	1	1	1	1	1	1	1	1	3
SAL0000660	hp	hypothetical protein	1	1	1	1	1	1	1	3	2	1	1	1	2	1	1	2	2	2	2	1	1	2	2	2	2	2	1	1	1	1	1	1	2	2	2	1	2	2	2	1	4
SAL0000661	ldh_2	L-lactate dehydrogenase	1	1	1	1	1	1	1	2	1	1	1	1	1	1	1	1	1	1	1	1	1	1	1	1	1	1	1	1	1	1	1	1	1	1	1	1	1	1	1	1	1
SAL0000662	dacA	D-alanyl-D-alanine carboxypeptidase DacA precursor	1	1	1	4	1	1	2	3	1	2	1	5	1	1	1	1	1	1	1	1	1	1	1	1	1	1	1	1	1	1	1	1	1	1	1	1	1	1	1	1	1
SAL0000663	trxA_1	Thioredoxin	1	1	1	1	1	1	1	1	1	1	1	1	1	1	1	1	1	1	1	1	1	1	1	1	1	1	1	1	1	1	1	1	1	1	1	1	1	1	1	1	2
SAL0000664	ntpJ	Potassium/sodium uptake protein NtpJ	1	1	1	1	1	1	1	1	1	1	1	1	1	1	1	1	1	1	1	1	1	1	1	1	1	1	1	3	1	1	1	1	1	1	1	1	1	1	1	1	2
SAL0000665	uvrC	UvrABC system protein C	1	1	1	3	1	1	1	2	1	1	1	1	1	1	1	1	1	1	1	1	1	1	1	1	1	1	3	2	1	1	1	1	1	1	1	1	1	1	1	1	4
SAL0000666	rsmA	Ribosomal RNA small subunit methyltransferase A	1	1	1	1	1	1	1	1	1	1	1	1	1	1	1	1	1	1	1	1	1	1	1	1	1	1	1	1	3	1	1	1	1	1	1	1	1	1	1	1	2
SAL0000667	group_2189	potassium transport protein Kup	1	1	1	1	1	1	2	5	1	2	1	1	1	1	1	1	1	1	1	1	1	1	1	1	1	1	1	3	1	1	1	1	1	1	1	4	1	1	1	1	1
SAL0000668	glyQ	Glycine--tRNA ligase alpha subunit	1	1	2	1	1	1	1	3	1	1	1	1	1	1	2	1	1	1	1	1	1	1	1	1	1	1	1	3	1	2	1	2	1	1	1	1	1	1	1	1	1
SAL0000669	pstC	Phosphate transport system permease protein PstC	1	1	1	1	1	1	2	4	1	2	1	1	1	1	1	1	1	1	1	1	1	1	1	1	1	1	1	3	1	1	1	1	1	1	1	1	1	1	1	1	1
SAL0000670	fba	Fructose-bisphosphate aldolase	1	1	2	2	1	2	2	2	1	2	1	3	1	1	2	1	1	1	1	1	1	1	1	1	1	1	2	2	2	2	1	2	1	1	1	1	1	1	1	1	2
SAL0000671	dhaL	PTS-dependent dihydroxyacetone kinase, ADP-binding subunit DhaL	1	1	2	1	1	1	1	1	1	1	1	1	1	1	1	1	1	1	1	1	1	1	1	1	1	1	1	1	1	1	1	1	1	1	1	1	1	1	1	1	1
SAL0000672	recN	DNA repair protein RecN	1	1	1	1	1	1	1	1	1	1	1	1	1	1	1	1	1	1	1	1	1	1	1	1	1	1	1	1	1	1	1	1	1	1	1	1	1	1	1	1	2
SAL0000673	spoIIIE	DNA translocase SpoIIIE	1	1	1	1	1	1	1	1	1	1	1	1	1	1	1	1	1	1	1	1	1	1	1	1	1	1	1	1	1	1	1	1	1	1	1	1	1	1	1	1	2
SAL0000674	lgt	Prolipoprotein diacylglyceryl transferase	1	1	1	1	1	1	1	1	1	1	1	1	1	1	1	1	3	1	1	1	1	1	1	1	1	1	1	2	1	1	1	1	1	1	1	1	1	1	1	1	4
SAL0000675	tlyA	16S/23S rRNA (cytidine-2'-O)-methyltransferase TlyA	1	1	1	1	1	1	1	1	1	1	1	1	1	1	1	1	1	1	1	1	1	1	1	1	1	1	1	1	1	1	1	1	1	1	1	1	1	1	1	1	2
SAL0000676	ycaM_3	Inner membrane transporter YcaM	1	1	1	1	1	1	1	3	1	1	1	4	1	1	2	1	1	1	1	1	1	1	1	1	1	1	1	1	1	1	1	1	1	1	1	1	1	1	1	1	5
SAL0000677	hp	hypothetical protein	1	1	2	1	1	1	1	1	1	1	1	1	1	1	2	1	1	1	1	1	1	1	1	1	1	1	1	1	1	2	1	2	1	1	1	1	1	1	1	1	1
**SAL0000678**	**ybhL**	**Inner membrane protein YbhL**	1	1	1	5	2	1	1	3	1	1	1	10	7	2	1	1	1	1	1	1	1	1	1	1	1	1	1	8	1	1	1	1	1	1	1	4	1	1	1	9	6
SAL0000679	pepN	Aminopeptidase N	1	1	1	1	1	4	1	2	1	1	1	1	1	1	1	1	1	1	1	1	1	1	1	1	1	1	1	2	1	1	1	1	1	1	1	1	1	1	1	1	3
SAL0000680	ywqD	Tyrosine-protein kinase YwqD	1	1	2	3	1	5	1	2	1	1	1	1	1	1	2	1	1	1	1	1	1	1	1	1	1	1	3	2	1	2	1	2	1	1	1	1	1	1	1	1	4
SAL0000681	exoA_1	Exodeoxyribonuclease	1	1	2	1	1	1	1	1	1	1	1	1	1	1	2	1	1	1	1	1	1	1	1	1	1	1	1	1	1	2	1	2	1	1	1	1	1	1	1	1	3
SAL0000682	xpt	Xanthine phosphoribosyltransferase	1	1	1	1	1	1	1	1	1	1	1	1	1	1	1	1	1	1	1	1	1	1	1	1	1	1	1	1	1	1	1	1	1	1	1	3	1	1	1	1	2
SAL0000683	pbpB	Penicillin-binding protein 2B	1	1	1	1	1	1	1	1	1	1	1	1	1	1	1	1	1	1	1	1	1	1	1	1	1	1	1	2	1	1	1	1	1	1	1	1	1	1	1	1	3
SAL0000684	pepE_3	Aminopeptidase E	1	1	1	1	1	1	1	2	1	1	1	1	1	1	1	1	1	1	1	1	1	1	1	1	1	1	1	1	1	1	1	1	1	1	1	1	1	1	1	1	1
SAL0000685	parC	DNA topoisomerase 4 subunit A	1	1	2	1	1	1	1	3	1	1	1	5	1	1	1	1	1	1	1	1	1	1	1	1	1	1	1	1	4	1	1	1	1	1	1	1	1	1	1	1	1
SAL0000686	group_2207	heat shock protein GrpE	1	1	1	1	1	1	1	1	1	1	1	2	1	1	1	1	1	1	1	1	1	1	1	1	1	1	1	1	1	1	1	1	1	1	1	1	1	1	1	1	3
SAL0000687	rsmH	Ribosomal RNA small subunit methyltransferase H	1	1	1	1	1	1	1	1	1	1	1	1	1	1	1	1	1	1	1	1	1	1	1	1	1	1	1	1	1	1	1	1	1	1	1	1	1	1	1	1	2
SAL0000688	citF	Citrate lyase alpha chain	1	1	2	1	1	1	3	1	1	3	1	1	1	1	2	1	1	1	1	1	1	1	1	1	1	1	1	1	1	2	1	2	1	1	1	1	1	1	1	1	4
SAL0000689	yajL	Chaperone protein YajL	1	1	2	1	1	1	1	1	1	1	1	1	1	1	2	1	1	1	1	1	1	1	1	1	1	1	1	1	1	2	1	2	1	1	1	1	1	1	1	1	1
SAL0000690	group_2211	putative ABC transporter ATP-binding protein	1	1	1	1	1	1	1	3	1	1	1	5	1	1	1	1	1	1	1	1	1	1	1	1	1	1	1	2	1	1	1	1	1	1	1	1	1	1	1	1	4
SAL0000691	ybeY	Endoribonuclease YbeY	1	1	1	1	1	1	1	1	1	1	1	1	1	1	1	1	1	1	1	1	1	1	1	1	1	1	1	1	2	1	1	1	1	1	1	1	1	1	1	1	1
SAL0000692	yihX	Alpha-D-glucose-1-phosphate phosphatase YihX	1	1	1	1	1	1	1	1	1	1	1	1	1	1	1	1	1	1	1	1	1	1	1	1	1	1	1	1	2	1	1	1	1	1	1	1	1	1	1	1	3
SAL0000693	group_2214	mevalonate kinase	1	1	1	1	1	1	1	1	1	1	1	1	1	1	1	1	1	1	1	1	1	1	1	1	1	1	1	1	1	1	1	1	1	1	1	1	1	1	1	1	2
SAL0000694	group_2215	Putative zinc metalloprotease	1	1	1	1	1	1	2	1	1	2	1	1	1	1	1	1	1	1	1	1	1	1	1	1	1	1	1	1	3	1	1	1	1	1	1	1	1	1	1	1	1
SAL0000695	group_2216	Putative 2-hydroxyacid dehydrogenase	1	1	1	1	1	1	1	1	1	1	1	1	1	1	1	1	1	1	1	1	1	1	1	1	1	1	1	1	1	1	1	1	1	1	1	1	1	1	1	1	2
SAL0000696	hp	hypothetical protein	1	1	2	1	1	4	1	6	1	1	1	1	1	1	2	1	1	1	1	1	1	1	1	1	1	1	1	1	1	2	1	2	1	1	1	3	1	1	1	1	5

SAL0000697	dppE_2	Dipeptide-binding protein DppE precursor	1	1	1	1	1	1	1	2	1	1	1	4	1	1	1	1	1	1	1	1	1	1	1	1	1	1	1	2	3	1	1	1	1	1	1	1	1	1	1	1	1
SAL0000698	murF	UDP-N-acetylmuramoyl-tripeptide--D-alanyl-D-alanine ligase	1	1	1	1	1	1	1	2	1	4	1	1	1	1	1	1	1	1	1	1	1	1	1	1	1	1	1	3	1	1	1	1	1	1	1	1	1	1	1	1	1
SAL0000699	hp	hypothetical protein	1	1	1	1	1	1	1	1	1	1	1	1	1	1	1	1	1	1	1	1	1	1	1	1	1	1	1	1	1	1	1	1	1	1	1	1	1	1	1	1	2
SAL0000700	gltX	Glutamate--tRNA ligase	1	1	1	1	1	1	1	1	1	1	1	1	1	1	1	1	1	1	1	1	1	1	1	1	1	1	1	1	1	1	1	1	1	1	1	2	1	1	1	1	1
SAL0000701	group_2222	CamS sex pheromone cAM373 precursor	1	1	1	1	1	1	1	1	1	1	1	1	1	1	1	1	1	1	1	1	1	1	1	1	1	1	1	1	1	1	1	1	1	1	1	1	1	1	1	1	2
SAL0000702	pepO_1	Neutral endopeptidase	1	1	2	1	1	1	1	6	1	1	1	1	1	1	1	1	1	1	1	1	1	1	1	1	1	1	1	3	1	1	1	1	1	1	1	1	1	1	5	1	4
SAL0000703	yqfL	Putative pyruvate, phosphate dikinase regulatory protein	1	1	2	3	1	5	1	4	1	1	1	1	1	1	2	1	1	1	1	1	1	1	1	1	1	1	3	2	1	2	1	2	1	1	1	1	1	1	1	1	1
SAL0000704	aspT	Aspartate/alanine antiporter	1	1	1	1	1	1	1	2	1	1	1	1	1	1	1	1	1	1	1	1	1	1	1	1	1	1	1	4	1	1	1	1	1	1	1	1	1	1	1	1	3
SAL0000705	group_2226	CAAX amino terminal protease self- immunity	1	1	2	1	1	1	1	1	1	1	1	1	1	1	2	1	1	1	1	1	1	1	1	1	1	1	1	1	1	2	1	2	1	1	1	1	1	1	1	1	1
SAL0000706	walK_2	Sensor protein kinase WalK	1	1	1	2	1	1	1	1	1	1	1	4	1	1	1	1	1	1	1	1	1	1	1	1	1	1	2	1	5	1	1	1	1	1	1	1	1	1	1	1	3
SAL0000707	rplL	50S ribosomal protein L7/L12	1	1	1	1	1	1	1	1	1	1	1	2	1	1	1	1	1	1	1	1	1	1	1	1	1	1	1	1	1	1	1	1	1	1	1	1	1	1	1	1	1
SAL0000708	araQ_1	L-arabinose transport system permease protein AraQ	1	1	5	2	1	1	1	1	1	1	1	3	1	1	1	1	1	1	1	1	1	1	1	1	1	1	2	1	1	1	1	1	1	1	1	1	1	1	1	1	4
SAL0000709	xerC_2	Tyrosine recombinase XerC	1	1	2	1	1	1	1	1	1	1	1	1	1	1	1	1	1	1	1	1	1	1	1	1	1	1	1	1	1	1	1	1	1	1	1	1	1	1	1	1	3
SAL0000710	serS	Serine--tRNA ligase	1	1	1	1	1	1	1	1	1	1	1	1	1	1	1	1	1	1	1	1	1	1	1	1	1	1	1	1	1	1	1	1	1	1	1	1	1	1	1	1	2
SAL0000711	gatB	Aspartyl/glutamyl-tRNA(Asn/Gln) amidotransferase subunit B	1	1	1	1	1	1	1	1	1	1	1	4	1	1	2	1	1	1	1	1	1	1	1	1	1	1	1	1	1	2	1	2	1	1	1	1	1	1	1	1	3
SAL0000712	ytgP_1	putative cell division protein YtgP	1	1	1	1	1	1	1	1	1	1	1	3	1	1	1	1	1	1	1	1	1	1	1	1	1	1	1	1	1	1	1	1	1	1	1	1	1	1	1	1	2
SAL0000713	hp	hypothetical protein	1	1	1	1	1	1	2	1	1	2	1	1	1	1	1	1	1	1	1	1	1	1	1	1	1	1	1	1	1	1	1	1	1	1	1	1	1	1	1	1	3
SAL0000714	malP_2	Maltose phosphorylase	1	1	1	1	1	1	1	1	1	1	1	1	1	1	1	1	1	1	1	1	1	1	1	1	1	1	1	1	1	1	1	1	1	1	1	1	1	1	1	1	2
SAL0000715	ftsA	Cell division protein FtsA	1	1	2	1	1	3	1	1	1	1	1	1	1	1	1	1	1	1	1	1	1	1	1	1	1	1	1	1	4	1	1	1	1	1	1	1	1	1	1	1	1
SAL0000716	rluD_2	Ribosomal large subunit pseudouridine synthase D	1	1	1	1	1	1	1	1	1	1	1	1	1	1	1	1	1	1	1	1	1	1	1	1	1	1	1	1	1	1	1	1	1	1	1	1	1	1	1	1	2
SAL0000717	group_2238	Sortase family protein	1	1	1	1	1	1	1	1	1	1	1	3	1	1	1	1	1	1	1	1	1	1	1	1	1	1	1	1	1	1	1	1	1	1	1	1	1	1	1	1	2
SAL0000718	galT	Galactose-1-phosphate uridylyltransferase	1	1	1	1	1	1	1	1	1	1	1	1	1	1	1	1	1	1	1	1	1	1	1	1	1	1	1	1	2	1	1	1	1	1	1	1	1	1	1	1	3
SAL0000719	infC	Translation initiation factor IF-3	2	2	2	2	2	2	2	2	2	2	2	2	2	2	2	2	2	2	2	2	2	2	2	2	2	2	2	2	2	2	2	2	2	2	2	2	2	2	2	2	3
SAL0000720	rpoB	DNA-directed RNA polymerase subunit beta	1	1	1	1	1	4	1	1	1	1	1	2	1	1	1	1	1	1	1	1	1	1	1	1	1	1	1	3	1	1	1	1	1	1	1	1	1	1	1	1	5
SAL0000721	nadE	NH(3)-dependent NAD( ) synthetase	1	1	1	1	1	1	1	2	1	1	1	1	1	1	1	1	1	1	1	1	1	1	1	1	1	1	1	1	1	1	1	1	1	1	1	1	1	1	1	1	3
SAL0000722	hp	hypothetical protein	1	1	1	1	1	1	1	1	1	1	1	1	1	1	1	1	1	1	1	1	1	1	1	1	1	1	1	1	1	1	1	1	1	1	1	1	1	1	1	1	2
SAL0000723	pspA_3	Phosphoserine phosphatase 1	1	1	2	1	1	1	1	1	1	1	1	1	1	1	2	1	1	1	1	1	1	1	1	1	1	1	1	1	1	2	1	2	1	1	1	1	1	1	1	1	1
SAL0000724	rfbX_1	Putative O-antigen transporter	1	1	2	1	1	1	1	1	1	1	1	1	1	1	1	1	1	1	1	1	1	1	1	1	1	1	1	1	1	1	1	1	1	1	1	1	1	1	1	1	3
SAL0000725	group_2246	putative ABC transporter ATP-binding protein	1	1	1	2	1	4	1	1	1	1	1	1	1	1	1	1	1	1	1	1	1	1	1	1	1	1	2	5	1	1	1	1	1	1	1	1	1	1	1	1	3
SAL0000726	hp	hypothetical protein	1	1	1	1	1	1	1	1	1	1	1	1	1	1	1	1	1	1	1	1	1	1	1	1	1	1	1	1	1	1	1	1	1	1	1	1	1	1	1	1	2
SAL0000727	glnP_2	Glutamine transport system permease protein GlnP	1	1	1	1	1	1	1	1	1	1	1	1	1	1	1	1	1	1	1	1	1	1	1	1	1	1	1	1	1	1	1	1	1	1	1	1	1	1	1	1	2
SAL0000728	fhs1_2	Formate--tetrahydrofolate ligase 1	1	1	1	1	1	1	1	1	1	1	1	1	1	1	1	1	1	1	1	1	1	1	1	1	1	1	1	1	1	1	1	1	1	1	1	1	1	1	1	1	2
SAL0000729	hp	hypothetical protein	1	1	1	1	1	1	1	1	1	1	1	2	1	1	1	1	1	1	1	1	1	1	1	1	1	1	3	1	1	1	1	1	1	1	1	1	1	1	1	1	1
SAL0000730	pucK	Uric acid permease PucK	1	1	1	2	1	1	1	1	1	1	1	1	1	1	1	1	1	1	1	1	1	1	1	1	1	1	1	1	1	1	1	1	1	1	1	1	1	1	1	1	3
SAL0000731	licT_3	Transcription antiterminator LicT	1	1	1	1	1	1	1	1	1	1	1	1	1	1	2	1	1	1	1	1	1	1	1	1	1	1	1	1	1	1	1	1	1	1	1	1	1	1	1	1	3
SAL0000732	group_2253	ribosomal-protein-alanine N-acetyltransferase	1	1	2	1	1	1	1	1	1	1	1	1	1	1	2	1	1	1	1	1	1	1	1	1	1	1	1	1	1	2	1	2	1	1	1	1	1	1	1	1	3
SAL0000733	nrnA_2	putative bifunctional oligoribonuclease and PAP phosphatase NrnA	1	1	1	1	1	1	1	1	1	1	1	1	1	1	1	1	1	1	1	1	1	1	1	1	1	1	1	1	1	1	1	1	1	1	1	1	1	1	1	1	2
SAL0000734	group_2255	Farnesyl diphosphate synthase	1	1	1	1	1	1	2	1	1	2	1	1	1	1	1	1	1	1	1	1	1	1	1	1	1	1	1	1	1	1	1	1	1	1	1	1	1	1	1	1	3
SAL0000735	dapA	4-hydroxy-tetrahydrodipicolinate synthase	1	1	2	1	1	1	1	2	1	1	1	1	1	1	2	1	1	1	1	1	1	1	1	1	1	1	1	2	1	2	1	2	1	1	1	1	1	1	1	1	3
SAL0000736	pbpX_3	Putative penicillin-binding protein PbpX	1	1	1	1	1	4	1	1	1	1	1	1	1	1	1	1	1	1	1	1	1	1	1	1	1	1	1	1	2	1	1	1	1	1	1	1	1	1	1	1	3
SAL0000737	corA	Magnesium transport protein CorA	1	1	2	1	1	1	1	1	1	1	1	3	1	1	2	1	1	1	1	1	1	1	1	1	1	1	1	5	1	2	1	2	1	1	1	1	1	1	1	1	4
SAL0000738	group_2259	Competence protein CoiA-like family protein	1	1	1	1	1	1	1	1	1	1	1	1	1	1	1	1	1	1	1	1	1	1	1	1	1	1	3	1	1	1	1	1	1	1	1	1	1	1	1	1	2
SAL0000739	dnaK	Chaperone protein DnaK	1	1	1	1	1	1	1	2	1	1	1	1	1	1	1	1	1	1	1	1	1	1	1	1	1	1	1	1	3	1	1	1	1	1	1	1	1	1	1	1	1
SAL0000740	ysdC	Putative aminopeptidase YsdC	1	1	3	1	1	1	2	1	1	2	1	1	1	1	1	1	1	1	1	1	1	1	1	1	1	1	1	1	1	1	1	1	1	1	1	1	1	1	1	1	4
SAL0000741	rplW	50S ribosomal protein L23	1	1	1	2	1	1	1	1	1	1	1	1	1	1	1	1	1	1	1	1	1	1	1	1	1	1	2	1	1	1	1	1	1	1	1	1	1	1	1	1	1
SAL0000742	priA_1	Primosomal protein N'	1	1	1	1	1	1	1	3	1	1	1	1	1	1	1	1	1	1	1	1	1	1	1	1	1	1	1	1	1	1	1	1	1	1	1	1	1	1	1	1	2
SAL0000743	group_2264	flavodoxin	1	1	1	1	1	1	1	3	1	1	1	2	1	1	1	1	1	1	1	1	1	1	1	1	1	1	1	1	1	1	1	1	1	1	1	1	1	1	1	1	1
SAL0000744	hp	hypothetical protein	1	1	1	1	1	1	1	1	1	1	1	1	1	1	1	1	1	1	1	1	1	1	1	1	1	1	1	1	1	1	1	1	1	1	1	1	1	1	1	1	2
SAL0000745	group_2266	Flavodoxin	1	1	1	1	1	1	1	1	1	1	1	1	1	1	1	1	1	1	1	1	1	1	1	1	1	1	1	1	2	1	1	1	1	1	1	1	1	1	1	1	1
SAL0000746	hp	hypothetical protein	1	1	1	1	1	2	1	1	1	1	1	3	1	1	1	1	1	1	1	1	1	1	1	1	1	1	1	1	1	1	1	1	1	1	1	1	1	1	1	1	1
SAL0000747	manY	Mannose permease IIC component	1	1	1	1	1	2	1	1	1	1	1	3	1	1	1	1	1	1	1	1	1	1	1	1	1	1	1	1	1	1	1	1	1	1	1	1	1	1	1	1	1
SAL0000748	slyA	Transcriptional regulator SlyA	1	1	2	1	1	1	1	1	1	1	1	1	1	1	2	1	1	1	1	1	1	1	1	1	1	1	1	1	1	2	1	2	1	1	1	1	1	1	1	1	1
SAL0000749	group_2270	LemA family protein	1	1	1	1	1	1	1	3	1	1	1	1	1	1	1	1	1	1	1	1	1	1	1	1	1	1	1	1	1	1	1	1	1	1	1	1	1	1	1	1	2
SAL0000750	gmuF	putative mannose-6-phosphate isomerase GmuF	1	1	1	1	1	1	1	1	1	1	1	1	1	1	1	1	1	1	1	1	1	1	1	1	1	1	1	1	1	1	1	1	1	1	1	1	1	1	1	1	2
SAL0000751	hp	hypothetical protein	1	1	1	1	1	1	1	1	1	1	1	1	1	1	3	1	1	1	1	1	1	1	1	1	1	1	1	1	1	1	1	1	1	1	1	1	1	1	1	1	2
SAL0000752	artM_2	Arginine transport ATP-binding protein ArtM	1	1	1	1	1	1	1	1	1	1	1	3	1	1	1	1	1	1	1	1	1	1	1	1	1	1	1	1	1	1	1	1	1	1	1	1	1	1	1	1	2
SAL0000753	relA	Bifunctional (p)ppGpp synthase/hydrolase RelA	1	1	2	1	1	1	1	2	1	1	1	3	1	1	2	1	1	1	1	1	1	1	1	1	1	1	1	2	1	2	1	2	1	1	1	1	1	1	1	1	4
SAL0000754	proS	Proline--tRNA ligase	1	1	1	1	1	1	1	2	1	1	1	1	1	1	1	1	1	1	1	1	1	1	1	1	1	1	1	1	1	1	1	1	1	1	1	1	1	1	1	1	1
SAL0000755	group_2276	Putative universal stress protein	1	1	1	2	1	1	1	3	1	1	1	1	1	1	1	1	1	1	1	1	1	1	1	1	1	1	2	1	1	1	1	1	1	1	1	1	1	1	1	1	4
SAL0000756	dtpT	Di-/tripeptide transporter	1	1	3	1	1	1	1	4	1	1	1	1	1	1	1	1	1	1	1	1	1	1	1	1	1	1	1	1	1	1	1	1	1	1	1	1	1	1	1	1	2
SAL0000757	comEA	ComE operon protein 1	1	1	2	1	1	1	1	2	1	1	1	1	1	1	2	1	1	1	1	1	1	1	1	1	1	1	1	2	1	2	1	2	1	1	1	1	1	1	1	1	1
SAL0000758	hp	hypothetical protein	1	1	1	1	1	1	1	1	1	1	1	1	1	1	1	1	1	1	1	1	1	1	1	1	1	1	1	1	1	1	1	1	1	1	1	1	1	1	1	1	2
SAL0000759	yheS_2	putative ABC transporter ATP-binding protein YheS	1	1	1	1	1	1	1	1	1	1	1	1	1	1	2	1	1	1	1	1	1	1	1	1	1	1	1	1	1	2	1	2	1	1	1	1	1	1	1	1	3
SAL0000760	thiI	putative tRNA sulfurtransferase	1	1	1	1	1	2	1	1	1	1	1	1	1	1	1	1	1	1	1	1	1	1	1	1	1	1	1	1	1	1	1	1	1	1	1	1	1	1	1	1	1

SAL0000761	hp	hypothetical protein	1	1	1	1	1	1	1	1	1	1	1	1	1	1	1	1	1	1	1	1	1	1	1	1	1	1	1	1	1	1	1	1	1	1	1	1	1	1	1	1	2
SAL0000762	yhdG_2	putative amino acid permease YhdG	1	1	5	1	1	1	4	3	1	4	1	1	1	1	2	1	1	1	1	1	1	1	1	1	1	1	1	3	1	2	1	2	1	1	1	1	1	1	1	1	1
SAL0000763	rpiA	Ribose-5-phosphate isomerase A	1	1	1	3	1	1	2	1	1	2	1	5	1	1	1	1	1	1	1	1	1	1	1	1	1	1	3	1	1	1	1	1	1	1	1	1	1	1	1	1	4
SAL0000764	cshB	DEAD-box ATP-dependent RNA helicase CshB	1	1	1	1	1	1	1	3	1	1	1	1	1	1	1	1	1	1	1	1	1	1	1	1	1	1	1	1	1	1	1	1	1	1	1	1	1	1	1	1	2
SAL0000765	lytR_4	Transcriptional regulator LytR	1	1	1	1	1	1	1	2	1	1	1	1	1	1	1	1	1	1	1	1	1	1	1	1	1	1	1	2	1	1	1	1	1	1	1	1	1	1	1	1	3
SAL0000766	lytR_1	Sensory transduction protein LytR	1	1	1	2	1	1	1	1	1	1	1	1	1	1	1	1	1	1	1	1	1	1	1	1	1	1	2	1	1	1	1	1	1	1	1	1	1	1	1	1	3
SAL0000767	group_2288	Putative universal stress protein	1	1	1	1	1	1	1	2	1	1	1	1	1	1	1	1	1	1	1	1	1	1	1	1	1	1	1	1	1	1	1	1	1	1	1	1	1	1	1	1	1
SAL0000768	hp	hypothetical protein	1	1	1	1	1	1	1	1	1	1	1	3	1	1	1	1	1	1	1	1	1	1	1	1	1	1	1	1	1	1	1	1	1	1	1	1	1	1	1	1	2
SAL0000769	group_2290	Recombinase	1	1	2	1	1	1	1	3	1	1	1	1	1	1	1	1	1	1	1	1	1	1	1	1	1	1	1	1	1	1	1	1	1	1	1	1	1	1	1	1	4
SAL0000770	addB	ATP-dependent helicase/deoxyribonuclease subunit B	1	1	3	1	1	1	1	1	1	1	1	4	1	1	1	1	1	1	1	1	1	1	1	1	1	1	1	2	1	1	1	1	1	1	1	1	1	1	1	1	5
SAL0000771	hp	hypothetical protein	1	1	1	1	1	1	1	1	1	1	1	1	1	1	1	1	1	1	1	1	1	1	1	1	1	1	1	1	1	1	1	1	1	1	1	1	1	1	1	1	2
SAL0000772	alx	Inner membrane protein alx	1	1	1	1	1	1	1	1	1	1	1	1	1	1	1	1	1	1	1	1	1	1	1	1	1	1	1	1	1	1	1	1	1	1	1	1	1	1	1	2	3
SAL0000773	hp	hypothetical protein	1	1	1	1	1	1	1	1	1	1	1	1	1	1	1	1	1	1	1	1	1	1	1	1	1	1	1	1	1	1	1	1	1	1	1	1	1	1	1	1	2
SAL0000774	lacM	Beta-galactosidase small subunit	1	1	1	1	1	1	1	1	1	1	1	2	1	1	1	1	1	1	1	1	1	1	1	1	1	1	1	1	1	1	1	1	1	1	1	1	1	1	1	1	3
SAL0000775	hp	hypothetical protein	1	1	2	1	1	1	1	1	1	1	1	1	3	1	2	1	1	1	1	1	1	1	1	1	1	1	1	1	1	2	1	2	1	1	1	1	1	1	1	1	1
SAL0000776	galM_2	Aldose 1-epimerase	1	1	3	2	1	2	2	4	1	2	1	2	1	1	3	1	1	1	1	1	1	1	1	1	1	1	2	6	2	3	1	3	1	1	1	1	1	1	1	1	5
SAL0000777	cysS	Cysteine--tRNA ligase	1	1	1	1	1	1	2	1	1	2	1	1	1	1	1	1	1	1	1	1	1	1	1	1	1	1	1	1	1	1	1	1	1	1	1	1	1	1	1	1	3
SAL0000778	group_2299	Aluminium resistance protein	1	1	1	1	1	1	1	3	1	1	1	1	1	1	1	1	1	1	1	1	1	1	1	1	1	1	1	1	1	1	1	1	1	1	1	1	1	1	1	1	2
SAL0000779	apu	Amylopullulanase precursor	1	1	3	1	1	1	1	1	1	1	1	2	1	1	1	1	1	1	1	1	1	1	1	1	1	1	1	1	1	1	1	1	1	1	1	1	1	1	1	1	1
SAL0000780	hp	hypothetical protein	1	1	1	1	1	1	2	1	1	2	1	1	1	1	1	1	1	1	1	1	1	1	1	1	1	1	1	1	1	1	1	1	1	1	1	1	1	1	1	1	3
SAL0000781	purB	Adenylosuccinate lyase	1	1	1	1	1	1	1	5	1	2	1	4	1	1	1	1	1	1	1	1	1	1	1	1	1	1	1	1	1	1	1	1	1	1	1	1	1	1	1	1	3
SAL0000782	group_2302	Calcium-transporting ATPase 1	1	1	1	1	1	1	1	3	1	1	1	1	1	1	1	1	1	1	1	1	1	1	1	1	1	1	1	1	1	1	1	1	1	1	1	1	1	1	1	1	2
SAL0000783	lacF_1	Lactose transport system permease protein LacF	1	1	2	3	1	1	1	2	1	1	1	1	1	1	2	1	1	1	5	1	1	1	1	1	1	1	3	2	1	2	1	2	1	1	1	1	1	1	1	1	4
SAL0000784	lysS	Lysine--tRNA ligase	1	1	1	1	1	1	1	1	1	1	1	4	1	1	1	1	1	1	1	1	1	1	1	1	1	1	1	1	1	2	1	2	1	1	1	1	1	1	1	1	3
SAL0000785	tagB	Putative CDP-glycerol:glycerophosphate glycerophosphotransferase	1	1	1	1	1	4	1	1	1	1	1	2	1	1	1	1	1	1	1	1	1	1	1	1	1	1	1	1	1	1	1	1	1	1	1	1	1	1	1	1	3
SAL0000786	ansA	L-asparaginase 1	1	1	1	1	1	1	1	1	1	1	1	1	1	1	1	1	1	1	1	1	1	1	1	1	1	1	1	1	1	1	1	1	1	1	1	1	1	1	1	1	2
SAL0000787	ydeH	Diguanylate cyclase YdeH	1	1	1	1	1	1	1	2	1	1	1	1	1	1	1	1	1	1	1	1	1	1	1	1	1	1	1	1	1	1	1	1	1	1	1	1	1	1	1	1	3
SAL0000788	tetA	Tetracycline resistance protein, class B	1	1	2	1	1	1	1	2	1	1	1	1	1	1	2	1	1	1	1	1	1	1	1	1	1	1	1	2	1	2	1	2	1	1	1	1	1	1	1	1	3
SAL0000789	rplB	50S ribosomal protein L2	1	1	1	1	1	1	1	2	1	1	1	1	1	1	1	1	1	1	1	1	1	1	1	1	1	1	1	1	1	1	1	1	1	1	1	1	1	1	1	1	1
SAL0000790	ecfT_2	Energy-coupling factor transporter transmembrane protein EcfT	1	1	1	1	1	1	1	1	1	1	1	1	1	1	1	1	1	1	1	1	1	1	1	1	1	1	1	1	1	1	1	1	1	1	1	1	1	1	1	1	2
SAL0000791	group_2311	inner membrane protein	1	1	2	1	1	1	1	2	1	1	1	1	1	1	2	1	1	1	1	1	1	1	1	1	1	1	1	2	1	2	1	2	1	1	1	1	1	1	1	1	3
SAL0000792	cbh_1	Choloylglycine hydrolase	1	1	1	2	1	1	1	1	1	1	1	1	1	1	1	1	1	1	1	1	1	1	1	1	1	1	2	1	1	1	1	1	1	1	1	1	1	1	1	1	1
SAL0000793	hp	hypothetical protein	1	1	1	1	1	1	1	1	1	1	1	1	1	1	1	1	1	1	1	1	1	1	1	1	1	1	1	1	1	2	1	2	1	1	1	1	1	1	1	1	3
SAL0000794	group_2314	Acyl-ACP thioesterase	1	1	2	1	1	4	1	1	1	1	1	3	1	1	2	1	1	1	1	1	1	1	1	1	1	1	1	1	1	2	1	2	1	1	1	1	1	1	1	1	5
SAL0000795	recG	ATP-dependent DNA helicase RecG	1	1	1	1	1	1	2	1	1	2	1	1	1	1	1	1	1	1	1	1	1	1	1	1	1	1	1	1	1	1	1	1	1	1	1	1	1	1	1	1	3
SAL0000796	yjbM	GTP pyrophosphokinase YjbM	1	1	1	1	1	1	1	1	1	1	1	1	1	1	1	1	1	1	1	1	1	1	1	1	1	1	1	2	1	1	1	1	1	1	1	1	1	1	1	1	3
SAL0000797	group_2317	Luciferase-like monooxygenase	1	1	3	1	1	1	2	1	1	2	1	1	1	1	1	1	1	1	1	1	1	1	1	1	1	1	1	1	1	1	1	1	1	1	1	1	1	1	1	1	1
SAL0000798	iphP_1	Tyrosine-protein phosphatase precursor	1	1	3	1	1	1	1	1	1	1	1	1	1	1	2	1	1	1	1	1	1	1	1	1	1	1	1	1	4	2	1	2	1	1	1	1	1	1	1	1	5
SAL0000799	priA_2	Primosomal protein N'	1	1	1	2	1	1	1	4	1	1	1	1	1	1	1	1	1	1	1	1	1	1	1	1	1	1	2	1	1	1	1	1	1	1	1	1	1	1	1	1	3
SAL0000800	group_232	glmZ(sRNA)-inactivating NTPase	1	1	2	3	1	1	1	1	1	1	1	1	1	1	2	1	1	1	1	1	1	1	1	1	1	1	3	1	1	2	1	2	1	1	1	1	1	1	1	1	1
SAL0000801	rbsK_1	Ribokinase	1	1	2	1	1	1	1	5	1	1	1	1	1	1	2	1	1	1	1	1	1	1	1	1	1	1	4	1	1	2	1	2	1	1	1	1	1	1	1	1	3
SAL0000802	manX_1	PTS system mannose-specific EIIAB component	1	1	1	1	1	1	1	2	1	1	1	1	1	1	1	1	1	1	1	1	1	1	1	1	1	1	1	1	1	1	1	1	1	1	1	1	1	1	1	1	1
SAL0000803	adcR	Transcriptional repressor AdcR	1	1	1	1	1	1	1	1	1	1	1	1	1	1	1	1	1	1	1	1	1	1	1	1	1	1	1	1	1	1	1	1	1	1	1	1	1	1	1	1	2
SAL0000804	glnH_2	ABC transporter glutamine-binding protein GlnH precursor	1	1	1	1	1	1	1	1	1	1	1	1	1	1	1	1	1	1	1	1	1	1	1	1	1	1	1	1	1	1	1	1	1	1	1	1	1	1	1	1	2
SAL0000805	group_2324	DNA-binding transcriptional activator PspC	1	1	1	1	1	1	1	1	1	1	1	1	1	1	1	1	1	1	1	1	1	1	1	1	1	1	1	1	1	1	1	1	1	1	1	1	1	1	1	1	2
SAL0000806	clpY	ATP-dependent protease ATPase subunit ClpY	1	1	1	1	1	1	1	1	1	1	1	3	1	1	1	1	1	1	1	1	1	1	1	1	1	1	1	1	1	1	1	1	1	1	1	1	1	1	1	1	2
SAL0000807	lexA	LexA repressor	1	1	1	1	1	1	1	2	1	1	1	3	1	1	1	1	1	1	1	1	1	1	1	1	1	1	1	1	1	1	1	1	1	1	1	1	1	1	1	1	1
SAL0000808	group_2327	CAAX amino terminal protease self- immunity	1	1	1	1	1	1	2	3	1	2	1	1	1	1	1	1	1	1	1	1	1	1	1	1	1	1	1	1	1	1	1	1	1	1	1	1	1	1	1	1	4
SAL0000809	bglF_6	PTS system beta-glucoside-specific EIIBCA component	1	1	1	1	1	1	1	5	1	1	1	1	1	1	1	1	1	1	1	1	1	1	1	1	1	1	1	1	3	2	1	2	1	1	1	1	1	1	1	1	4
SAL0000810	group_2329	putative peptidase	1	1	1	1	1	1	2	1	1	3	1	1	1	1	1	1	1	1	1	1	1	1	1	1	1	1	1	1	1	1	1	1	1	1	1	1	1	1	1	1	1
SAL0000811	nhaC	Na( )/H( ) antiporter NhaC	1	1	1	1	1	4	1	3	1	1	1	1	1	1	1	1	1	1	1	1	1	1	1	1	1	1	1	1	1	1	1	1	1	1	1	1	1	1	1	1	2
SAL0000812	hp	hypothetical protein	1	1	1	1	1	1	1	1	1	1	1	1	1	1	1	1	1	1	1	1	1	1	1	1	1	1	1	1	1	1	1	1	1	1	1	1	1	1	1	1	2
SAL0000813	murB	UDP-N-acetylenolpyruvoylglucosamine reductase	1	1	1	1	1	1	1	1	1	1	1	1	1	1	1	1	1	1	1	1	1	1	1	1	1	1	1	1	1	1	1	1	1	1	1	1	1	1	1	1	2
SAL0000814	udk	Uridine kinase	1	1	1	1	1	3	1	1	1	1	1	1	1	1	1	1	1	1	1	1	1	1	1	1	1	1	1	1	1	1	1	1	1	1	1	1	1	1	1	1	2
SAL0000815	group_2334	Matrixin	1	1	1	1	1	1	1	1	1	1	1	1	1	1	1	1	1	1	1	1	1	1	1	1	1	1	1	1	1	1	1	1	1	1	1	1	1	1	1	1	2
SAL0000816	glgA	Glycogen synthase	1	1	1	1	1	1	1	1	1	1	1	1	1	1	1	1	1	1	1	1	1	1	1	1	1	1	1	1	1	1	1	1	1	1	1	1	1	1	1	1	2
SAL0000817	manZ	Mannose permease IID component	1	1	2	1	1	1	3	2	1	3	1	5	1	1	2	1	1	1	1	1	1	1	1	1	1	1	1	2	4	2	1	2	1	1	1	1	1	1	1	1	1
SAL0000818	hp	hypothetical protein	1	1	1	1	1	1	1	1	1	1	1	1	1	1	1	1	1	1	1	1	1	1	1	1	1	1	1	1	1	1	1	1	1	1	1	1	1	1	1	1	2
SAL0000819	group_2338	Glycosyl hydrolases family 25	1	1	1	1	1	1	1	3	1	1	1	1	1	1	1	1	1	1	1	1	1	1	1	1	1	1	1	1	1	1	1	1	1	1	1	1	1	1	1	1	2
SAL0000820	rsgA_2	Putative ribosome biogenesis GTPase RsgA	1	1	1	1	1	1	1	1	1	1	1	1	1	1	1	1	1	1	1	1	1	1	1	1	1	1	1	1	1	1	1	1	1	1	1	1	1	1	1	1	2
SAL0000821	pta	Phosphate acetyltransferase	1	1	1	2	1	1	1	1	1	1	1	3	1	1	1	1	1	1	1	1	1	1	1	1	1	1	1	1	1	1	1	1	1	1	1	1	1	1	1	1	1
SAL0000822	pstB3_2	Phosphate import ATP-binding protein PstB 3	1	1	1	3	1	1	2	1	1	2	1	1	1	1	1	1	1	1	1	1	1	1	1	1	1	1	1	1	1	1	1	1	1	1	1	1	1	1	1	1	1
SAL0000823	ecfA1	Energy-coupling factor transporter ATP-binding protein EcfA1	1	1	1	1	1	1	1	1	1	1	1	1	1	1	1	1	1	1	1	1	1	1	1	1	1	1	1	1	1	1	1	1	1	1	1	1	1	1	1	1	2
SAL0000824	cycA_2	D-serine/D-alanine/glycine transporter	1	1	1	1	1	2	1	1	1	1	1	1	1	1	1	1	1	1	1	1	1	1	1	1	1	1	1	1	1	1	1	1	1	1	1	1	1	1	1	1	3

SAL0000825	bglF_2	PTS system beta-glucoside-specific EIIBCA component	1	1	2	1	1	1	1	2	1	1	1	1	1	1	2	1	1	1	1	1	1	1	1	1	1	1	1	2	1	2	1	2	1	1	1	4	1	1	1	1	3
SAL0000826	pyk	Pyruvate kinase	1	1	1	6	1	1	1	5	1	1	1	1	1	1	1	1	1	1	1	1	1	1	1	1	1	1	2	1	3	1	1	1	1	1	1	1	1	1	1	1	4
SAL0000827	simA	6-phospho-alpha-glucosidase 1	1	1	4	1	1	1	2	1	1	2	1	1	1	1	1	1	1	1	1	1	1	1	1	1	1	1	1	1	1	1	1	1	1	1	1	1	1	1	1	1	3
SAL0000828	mutX	8-oxo-dGTP diphosphatase	1	1	1	1	1	1	1	1	1	1	1	1	1	1	1	1	1	1	1	1	1	1	1	1	1	1	1	1	1	1	1	1	1	1	1	1	1	1	1	1	2
SAL0000829	hp	hypothetical protein	1	1	1	1	1	1	1	1	1	1	1	2	1	1	1	1	1	1	1	1	1	1	1	1	1	1	1	1	1	1	1	1	1	1	1	1	1	1	1	1	3
SAL0000830	mmuM	Homocysteine S-methyltransferase	1	1	1	1	1	1	1	1	1	1	1	1	1	1	1	1	1	1	1	1	1	1	1	1	1	1	1	1	1	1	1	1	1	1	1	1	1	1	1	1	2
SAL0000831	murAB	UDP-N-acetylglucosamine 1-carboxyvinyltransferase 2	1	1	2	1	1	1	1	1	1	1	1	1	1	1	2	1	1	1	1	1	1	1	1	1	1	1	1	1	1	2	1	2	1	1	1	1	1	1	1	1	3
SAL0000832	hp	hypothetical protein	1	1	1	1	1	1	1	1	1	1	1	1	1	1	1	1	1	1	1	1	1	1	1	1	1	1	1	1	1	1	1	1	1	1	1	1	1	1	1	1	2
SAL0000833	ydjZ	TVP38/TMEM64 family inner membrane protein YdjZ	1	1	1	1	1	1	1	1	1	1	1	1	1	1	1	1	1	1	1	1	1	1	1	1	1	1	1	1	1	1	1	1	1	1	1	1	1	1	1	2	1
SAL0000834	hp	hypothetical protein	1	1	1	1	1	3	1	1	1	1	1	1	1	1	1	1	1	1	1	1	1	1	1	1	1	1	1	1	1	1	1	1	1	1	1	1	1	1	1	1	2
SAL0000835	kmtR	HTH-type transcriptional regulator KmtR	1	1	1	1	1	1	1	1	1	1	1	1	2	1	1	1	1	1	1	1	1	1	1	1	1	1	1	1	1	1	1	1	1	1	1	1	1	1	1	1	1
SAL0000836	yecS	Inner membrane amino-acid ABC transporter permease protein YecS	1	1	2	1	1	1	1	1	1	1	1	1	1	1	2	1	1	1	1	1	1	1	1	1	1	1	1	1	1	2	1	2	1	1	1	1	1	1	1	1	3
SAL0000837	yicL_1	putative inner membrane transporter YicL	1	1	1	1	1	1	1	1	1	1	1	1	1	1	1	1	1	1	1	1	1	1	1	1	1	1	1	1	1	1	1	1	1	1	1	1	1	1	1	1	2
SAL0000838	group_2356	cytoplasmic glycerophosphodiester phosphodiesterase	1	1	2	1	1	1	1	1	1	1	1	1	1	1	2	1	1	1	1	1	1	1	1	1	1	1	1	1	1	2	1	2	1	1	1	1	1	1	1	1	3
SAL0000839	hp	hypothetical protein	1	1	1	1	1	1	1	1	1	1	1	1	1	1	1	1	1	1	1	1	1	1	1	1	1	1	1	1	1	1	1	1	1	1	1	1	1	1	1	1	2
SAL0000840	pheT_1	Phenylalanine--tRNA ligase beta subunit	1	1	1	1	1	1	1	1	1	1	1	1	1	1	1	1	1	1	1	1	1	1	1	1	1	1	1	1	1	1	1	1	1	1	1	1	1	1	1	1	2
SAL0000841	group_2359	Fibronectin type III domain protein	1	1	1	1	1	3	1	4	1	1	1	5	1	1	1	1	1	1	1	1	1	1	1	1	1	1	1	1	1	1	1	1	1	1	1	1	1	1	1	1	2
SAL0000842	yidA_2	Sugar phosphatase YidA	1	1	2	1	1	1	1	4	1	1	1	1	1	1	2	1	1	1	1	1	1	1	1	1	1	1	1	1	1	2	1	2	1	1	1	1	1	1	1	1	3
SAL0000843	rlmCD_2	23S rRNA (uracil-C(5))-methyltransferase RlmCD	1	1	1	1	1	1	1	3	1	1	1	1	1	1	1	1	1	1	1	1	1	1	1	1	1	1	1	1	1	1	1	1	2	1	1	1	1	1	1	1	4
SAL0000844	rplP	50S ribosomal protein L16	1	1	2	1	1	1	1	1	1	1	1	1	1	1	1	1	1	1	1	1	1	1	1	1	1	1	1	1	1	1	1	1	1	1	1	1	1	1	1	1	1
SAL0000845	hisS	Histidine--tRNA ligase	1	1	4	1	1	1	1	1	1	1	1	1	1	1	2	1	1	1	1	1	1	1	1	1	1	1	1	1	1	2	1	2	1	1	1	1	1	1	1	1	3
SAL0000846	carB_1	Carbamoyl-phosphate synthase large chain	1	1	1	1	1	1	2	1	1	2	1	5	1	1	1	1	1	1	1	1	1	1	1	1	1	1	1	4	1	1	1	1	1	1	1	1	1	1	1	1	3
SAL0000847	pyrP	Uracil permease	1	1	1	1	1	1	1	2	1	1	1	1	1	1	1	1	1	1	1	1	1	1	1	1	1	1	1	2	1	1	1	1	1	1	1	1	1	1	1	1	3
SAL0000848	ileS	Isoleucine--tRNA ligase	1	1	2	2	1	2	2	3	1	2	1	5	1	1	4	1	1	1	1	1	1	1	1	1	1	1	2	2	2	2	1	2	1	1	1	1	1	1	1	1	6
SAL0000849	trxB_2	Thioredoxin reductase	1	1	2	1	1	1	1	1	1	1	1	1	1	1	2	1	1	1	1	1	1	1	1	1	1	1	1	3	1	2	1	2	1	1	1	1	1	1	1	1	4
SAL0000850	rbgA	Ribosome biogenesis GTPase A	1	1	1	1	1	1	1	1	1	2	1	1	1	1	1	1	1	1	1	1	1	1	1	1	1	1	1	1	1	1	1	1	1	1	1	1	1	1	1	1	3
SAL0000851	group_2368	CAAX amino terminal protease self- immunity	1	1	1	1	1	1	1	1	1	1	1	1	1	1	1	1	1	1	1	1	1	1	1	1	1	1	1	1	2	1	1	1	1	1	1	1	1	1	1	1	1
SAL0000852	yveA	Aspartate-proton symporter	1	1	1	1	1	1	1	2	1	1	1	4	1	1	1	1	1	1	1	1	1	1	1	1	1	1	1	1	1	1	1	1	1	1	1	1	1	1	1	1	3
SAL0000853	hp	hypothetical protein	1	1	1	1	1	1	1	1	1	1	1	2	1	1	1	1	1	1	1	1	1	1	1	1	1	1	1	1	1	1	1	1	1	1	1	1	1	1	1	1	3
SAL0000854	xlyA	N-acetylmuramoyl-L-alanine amidase XlyA precursor	1	1	1	1	1	1	1	2	1	1	1	1	1	1	1	1	1	1	1	1	1	1	1	1	1	1	1	2	1	1	1	1	1	1	1	1	1	1	1	1	3
SAL0000855	lepA	Elongation factor 4	1	1	3	1	1	1	1	1	1	1	1	2	1	1	1	1	1	1	1	1	1	1	1	1	1	1	1	1	1	1	1	1	1	1	1	1	1	1	1	1	1
SAL0000856	prfB	Peptide chain release factor 2	1	1	1	1	1	1	1	2	1	1	1	1	1	1	1	1	1	1	1	1	1	1	1	1	1	1	1	1	1	1	1	1	1	1	1	1	1	1	1	1	3
SAL0000857	bglH_1	Aryl-phospho-beta-D-glucosidase BglH	1	1	1	1	1	1	1	1	1	1	1	1	1	1	1	1	1	1	1	1	1	1	1	1	1	1	1	1	1	1	1	1	1	1	1	1	1	1	1	1	2
SAL0000858	group_2374	mevalonate kinase	1	1	1	1	1	2	1	1	1	1	1	4	1	1	1	1	1	1	1	1	1	1	1	1	1	1	1	1	1	1	1	1	1	1	1	1	1	1	1	1	3
SAL0000859	ybbH_2	putative HTH-type transcriptional regulator YbbH	1	1	1	1	1	1	1	2	1	1	1	1	1	1	1	1	1	1	1	1	1	1	1	1	1	1	1	2	1	1	1	1	1	1	1	1	1	1	1	1	3
SAL0000860	prs_2	Ribose-phosphate pyrophosphokinase	1	1	2	2	1	2	2	2	1	2	1	2	1	1	2	1	1	1	1	1	1	1	1	1	1	1	2	2	2	2	1	2	1	1	1	1	1	1	1	1	3
SAL0000861	spxA_1	Regulatory protein Spx	1	1	1	1	1	1	1	3	1	1	1	2	1	1	1	1	1	1	1	1	1	1	1	1	1	1	1	1	1	1	1	1	1	1	1	1	1	1	1	1	1
SAL0000862	adk	Adenylate kinase	1	1	1	1	1	1	1	1	1	1	1	1	1	1	2	1	1	1	1	1	1	1	1	1	1	1	1	1	1	2	1	2	1	1	1	1	1	1	1	1	1
SAL0000863	folT	Folate transporter FolT	1	1	2	1	1	2	2	2	1	2	1	2	1	1	2	1	1	1	1	1	1	1	1	1	1	1	1	2	2	2	1	2	1	1	1	1	1	1	1	1	2
SAL0000864	yycF_2	Transcriptional regulatory protein YycF	1	1	2	1	1	1	1	3	1	1	1	1	1	1	1	1	1	1	1	1	1	1	1	1	1	1	1	1	1	1	1	1	1	1	1	1	1	1	1	1	1
SAL0000865	rplR	50S ribosomal protein L18	1	1	1	1	1	1	1	1	1	1	1	1	1	1	1	1	1	1	1	1	1	1	1	1	1	1	1	1	1	1	1	1	1	1	1	1	1	1	1	1	2
SAL0000866	mtnN	5'-methylthioadenosine/S-adenosylhomocysteine nucleosidase	1	1	2	1	1	3	1	1	1	1	1	1	1	1	2	1	1	1	1	1	1	1	1	1	1	1	1	1	1	2	1	2	1	1	1	1	1	1	1	1	1
SAL0000867	ppnK	putative inorganic polyphosphate/ATP-NAD kinase	1	1	2	1	1	1	1	1	1	1	1	3	1	1	2	1	1	1	1	1	1	1	1	1	1	1	1	1	1	2	1	2	1	1	1	1	1	1	1	1	1
SAL0000868	yheI_1	putative multidrug resistance ABC transporter ATP-binding/permease protein	1	1	1	1	1	1	1	2	1	1	1	1	1	1	1	1	1	1	1	1	1	1	1	1	1	1	1	1	1	1	1	1	1	1	1	1	1	1	1	1	1
SAL0000869	maeA	putative NAD-dependent malic enzyme 2	1	1	2	1	1	1	1	1	1	1	1	1	1	1	1	1	1	1	1	1	1	1	1	1	1	1	1	1	1	1	1	1	1	1	1	1	1	1	1	1	3
SAL0000870	murC	UDP-N-acetylmuramate--L-alanine ligase	1	1	2	1	1	1	1	2	1	1	1	1	1	1	2	1	1	1	1	1	1	1	1	1	1	1	1	2	1	2	1	2	1	1	1	1	1	1	1	1	3
SAL0000871	thiD	Hydroxymethylpyrimidine/phosphomethylpyrimidine kinase	1	1	1	1	1	1	1	1	1	1	1	1	1	1	1	1	1	1	1	1	1	1	1	1	1	1	1	1	2	1	1	1	1	1	1	1	1	1	1	1	3
SAL0000872	group_2388	tetratricopeptide repeat protein	1	1	1	1	1	1	1	1	1	1	1	1	1	1	1	1	1	1	1	1	1	1	1	1	1	1	1	1	1	1	1	1	1	1	1	1	1	1	1	1	2
SAL0000873	hp	hypothetical protein	1	1	1	1	1	1	1	1	1	1	1	1	1	1	1	1	1	1	1	1	1	1	1	1	1	1	1	1	1	1	1	1	1	1	1	1	1	1	1	1	2
SAL0000874	hsrA_1	putative transport protein HsrA	1	1	1	2	1	1	1	1	1	1	1	3	1	1	1	1	1	1	1	1	1	1	1	1	1	1	2	1	1	1	1	1	1	1	1	1	1	1	1	1	4
SAL0000875	sorC	Sorbitol operon regulator	1	1	1	1	1	1	1	1	1	1	1	1	1	1	1	1	1	1	1	1	1	1	1	1	1	1	2	1	1	1	1	1	1	1	1	1	1	1	1	1	1
SAL0000876	npr	NADH peroxidase	1	1	1	1	1	1	1	1	1	1	1	1	1	1	1	1	1	1	1	1	1	1	1	1	1	1	1	1	3	1	1	1	1	1	1	2	1	1	1	1	4
SAL0000877	group_2392	Putative TrmH family tRNA/rRNA methyltransferase	1	1	2	2	1	2	2	2	1	2	1	2	1	1	2	1	1	1	1	1	1	1	1	1	1	1	2	2	2	2	1	2	1	1	1	1	1	1	1	1	3
SAL0000878	group_2393	Glutamine transport ATP-binding protein GlnQ	1	1	1	1	1	1	1	2	1	1	1	1	1	1	1	1	1	1	1	1	1	1	1	1	1	1	1	1	1	1	1	1	1	1	1	1	1	1	1	1	1
SAL0000879	pepV	Beta-Ala-Xaa dipeptidase	1	1	4	1	1	1	1	2	1	1	1	1	1	1	1	1	1	1	1	1	1	1	1	1	1	1	1	1	1	1	1	1	1	1	1	1	1	1	1	1	3
SAL0000880	hp	hypothetical protein	1	1	1	1	1	2	1	1	1	1	1	1	1	1	1	1	1	1	1	1	1	1	1	1	1	1	1	1	1	1	1	1	1	1	1	1	1	1	1	1	3
SAL0000881	yciB_2	Putative L,D-transpeptidase YciB precursor	1	1	1	1	1	1	1	2	1	1	1	1	1	1	1	1	1	1	1	1	1	1	1	1	1	1	1	2	3	1	1	1	1	1	1	1	1	1	1	1	1
SAL0000882	hp	hypothetical protein	1	1	2	1	1	1	1	2	1	1	1	1	1	1	2	1	1	1	1	1	1	1	1	1	1	1	1	2	1	2	1	2	1	1	1	1	1	1	1	1	1
SAL0000883	murG	UDP-N-acetylglucosamine--N-acetylmuramyl-(pentapeptide) pyrophosphoryl-	1	1	1	1	1	1	1	1	1	1	1	1	1	1	1	1	1	1	1	1	1	1	1	1	1	1	1	1	1	1	1	1	1	1	1	3	1	1	1	1	2
SAL0000884	pyrR_1	Bifunctional protein pyrR	1	1	1	1	1	1	1	2	1	1	1	1	1	1	1	1	1	1	1	1	1	1	1	1	1	1	1	1	1	1	1	1	1	1	1	1	1	1	1	1	3
SAL0000885	xseA	Exodeoxyribonuclease 7 large subunit	1	1	1	1	1	1	1	1	1	1	1	1	1	1	1	1	1	1	1	1	1	1	1	1	1	1	1	1	1	1	1	1	1	1	1	1	1	1	1	1	2
SAL0000886	aadR	Transcriptional activatory protein AadR	1	1	2	1	1	3	1	2	1	1	1	1	1	1	2	1	1	1	1	1	1	1	1	1	1	1	1	2	1	2	1	2	1	1	1	1	1	1	1	1	4
SAL0000887	galM_1	Aldose 1-epimerase	1	1	1	2	1	1	1	1	1	1	1	1	1	1	1	1	1	1	1	1	1	1	1	1	1	1	2	1	1	1	1	1	1	1	1	1	1	1	1	1	3
SAL0000888	scrB	Sucrose-6-phosphate hydrolase	1	1	1	1	1	1	1	2	1	1	1	1	1	1	1	1	1	1	1	1	1	1	1	1	1	1	1	2	1	1	1	1	1	1	1	1	1	1	1	1	3

SAL0000889	group_2404	putative transporter YfdV	1	1	2	1	1	1	1	1	1	1	1	1	1	1	2	1	1	1	1	1	1	1	1	1	1	1	1	1	1	2	1	2	1	1	1	1	1	1	1	1	1
SAL0000890	nlhH_2	Carboxylesterase NlhH	1	1	1	1	1	3	1	1	1	1	1	1	1	1	1	1	1	1	1	1	1	1	1	1	1	1	1	1	1	1	1	1	1	1	1	1	1	1	1	1	2
SAL0000891	hp	hypothetical protein	1	1	2	1	1	1	1	2	1	1	1	1	1	1	2	1	1	1	1	1	1	1	1	1	1	1	1	2	1	2	1	2	1	1	1	1	1	1	1	1	1
SAL0000892	cfa	Cyclopropane-fatty-acyl-phospholipid synthase	1	1	1	2	1	1	1	1	1	1	1	1	1	1	1	1	1	1	1	1	1	1	1	1	1	1	1	1	1	1	1	1	1	1	1	1	1	1	1	1	1
SAL0000893	asD	Bifunctional aspartate aminotransferase and L-aspartate beta-decarboxylase	1	1	1	1	1	1	1	2	1	1	1	1	1	1	1	1	1	1	1	1	1	1	1	1	1	1	1	2	1	1	1	1	1	1	1	1	1	1	1	1	3
SAL0000894	tig	Trigger factor	1	1	2	1	1	1	1	1	1	1	1	1	1	1	3	1	1	1	1	1	1	1	1	1	1	1	1	1	1	2	1	2	4	1	1	1	1	1	1	1	1
SAL0000895	group_2412	CAAX amino terminal protease self- immunity	1	1	1	1	1	1	2	1	1	2	1	1	1	1	1	1	1	1	1	1	1	1	1	1	1	1	1	1	1	1	1	1	1	1	1	1	1	1	1	1	1
SAL0000896	glmS	Glutamine--fructose-6-phosphate aminotransferase [isomerizing]	1	1	1	1	1	1	1	1	1	1	1	1	1	1	1	1	1	1	1	1	1	1	1	1	1	1	1	1	1	1	1	1	1	1	1	1	1	1	1	1	2
SAL0000897	glvR_2	HTH-type transcriptional regulator GlvR	1	1	1	2	1	1	1	1	1	1	1	4	1	1	3	1	1	1	1	1	1	1	1	1	1	1	2	1	1	1	1	1	1	1	1	1	1	1	1	1	1
SAL0000898	sepF	Cell division protein SepF	1	1	1	1	1	1	1	1	1	1	1	1	1	1	1	1	1	1	1	1	1	1	1	1	1	1	1	1	1	1	1	1	1	1	1	2	1	1	1	1	1
SAL0000899	tyrS	Tyrosine--tRNA ligase	1	1	1	1	1	1	1	2	1	1	1	1	1	1	1	1	1	1	1	1	1	1	1	1	1	1	1	1	1	1	1	1	1	1	1	1	1	1	1	1	1
SAL0000900	ccpA_1	Catabolite control protein A	1	1	1	1	1	1	1	1	1	1	1	1	1	1	1	1	1	1	1	1	1	1	1	1	1	1	1	1	1	1	1	1	1	1	1	1	1	1	1	1	2
SAL0000901	hp	hypothetical protein	1	1	2	1	1	1	1	1	1	1	1	1	1	1	1	1	1	1	1	1	1	1	1	1	1	1	1	1	1	1	1	1	1	1	1	1	1	1	1	1	3
SAL0000902	hp	hypothetical protein	1	1	1	1	1	1	1	1	1	1	1	1	1	1	1	1	1	1	1	1	1	1	1	1	1	1	1	1	1	1	1	1	1	1	1	1	1	1	1	1	2
SAL0000903	ftsZ	Cell division protein FtsZ	1	1	1	1	1	1	2	1	1	2	1	1	1	1	1	1	1	1	1	1	1	1	1	1	1	1	4	1	1	1	1	1	1	1	1	1	1	1	1	1	3
SAL0000904	valS	Valine--tRNA ligase	1	1	2	2	1	2	2	3	1	2	1	2	1	1	2	1	1	1	1	1	1	1	1	1	1	1	2	2	2	2	1	2	1	1	1	1	1	1	1	1	2
SAL0000905	hp	hypothetical protein	1	1	1	1	1	1	1	3	1	1	1	1	1	1	1	1	1	1	1	1	1	1	1	1	1	1	1	1	1	1	1	1	1	1	1	1	1	1	1	1	2
SAL0000906	cobB	NAD-dependent protein deacetylase	1	1	1	1	1	1	2	1	1	2	1	1	1	1	1	1	1	1	1	1	1	1	1	1	1	1	1	1	1	1	1	1	1	1	1	1	1	1	1	1	3
SAL0000907	hp	hypothetical protein	1	1	1	1	1	1	1	1	1	1	1	1	1	1	1	1	1	1	1	1	1	1	1	1	1	1	1	1	1	1	1	1	1	1	1	1	1	1	1	1	2
SAL0000908	group_2425	phosphatidylglycerophosphatase B	1	1	1	1	1	1	1	1	1	1	1	1	1	1	1	1	1	1	1	1	1	1	1	1	1	1	1	1	3	1	1	1	1	1	1	1	1	1	1	1	2
SAL0000909	hp	hypothetical protein	1	1	1	1	1	1	1	1	1	1	1	1	1	1	1	1	1	1	1	1	1	1	1	1	1	1	1	1	1	1	1	1	1	1	1	1	1	1	1	1	2
SAL0000910	rluD_1	Ribosomal large subunit pseudouridine synthase D	1	1	1	1	1	1	1	2	1	1	1	1	1	1	1	1	1	1	1	1	1	1	1	1	1	1	1	1	1	1	1	1	1	1	1	1	1	1	1	1	1
SAL0000911	tagA	Putative N-acetylmannosaminyltransferase	1	1	1	1	1	1	1	2	1	1	1	1	1	1	1	1	1	1	1	1	1	1	1	1	1	1	1	1	1	1	1	1	1	1	1	1	1	1	1	1	3
SAL0000912	garB	Glutathione amide reductase	1	1	1	5	1	4	1	3	1	1	1	1	1	1	1	1	1	1	1	1	1	1	1	1	1	1	1	1	1	1	1	1	1	1	1	1	1	1	1	1	2
SAL0000913	fgs	Folylpolyglutamate synthase	1	1	1	2	1	1	1	4	1	1	1	5	3	1	1	1	1	1	1	1	1	1	1	1	1	1	2	1	1	1	1	1	1	1	1	1	1	1	1	1	6
SAL0000914	pepE_1	Aminopeptidase E	1	1	1	1	1	1	1	2	1	1	1	1	1	1	1	1	1	1	1	1	1	1	1	1	1	1	1	1	1	1	1	1	1	1	1	1	1	1	1	1	1
SAL0000915	rpoC	DNA-directed RNA polymerase subunit beta'	1	1	6	5	1	7	4	2	1	4	1	3	1	1	2	1	1	1	1	1	1	1	1	1	1	1	5	2	3	2	1	2	1	1	1	1	1	1	1	1	3
SAL0000916	glgP	Glycogen phosphorylase	1	1	1	1	1	1	1	2	1	1	1	1	1	1	1	1	1	1	1	1	1	1	1	1	1	1	1	1	1	1	1	1	1	1	1	1	1	1	1	1	1
SAL0000917	pepO_2	Neutral endopeptidase	1	1	1	2	1	1	1	3	1	1	1	1	1	1	1	1	1	1	1	1	1	1	1	1	1	1	2	1	1	1	1	1	1	1	1	1	1	1	1	1	4
SAL0000918	mepH	Murein DD-endopeptidase MepH precursor	1	1	1	1	1	1	1	1	1	1	1	1	1	1	1	1	1	1	1	1	1	1	1	1	1	1	1	1	1	1	1	1	1	1	1	1	1	1	1	1	2
SAL0000919	groL	60 kDa chaperonin	1	1	1	1	1	1	1	3	1	1	1	1	1	1	1	1	1	1	1	1	1	1	1	1	1	1	1	1	1	1	1	1	1	1	1	1	1	1	1	1	2
SAL0000920	epsH	Putative glycosyltransferase EpsH	1	1	1	1	1	1	1	1	1	1	1	2	1	1	1	1	1	1	1	1	1	1	1	1	1	1	1	1	1	1	1	1	1	1	1	1	1	1	1	1	3
SAL0000921	pstB3_1	Phosphate import ATP-binding protein PstB 3	1	1	1	1	1	1	1	1	1	1	1	2	1	1	1	1	1	1	1	1	1	1	1	1	1	1	1	1	1	1	1	1	1	1	1	1	1	1	1	1	1
SAL0000922	hp	hypothetical protein	1	1	1	1	1	1	1	1	1	1	1	1	1	1	1	1	1	1	1	1	1	1	1	1	1	1	1	1	1	1	1	1	1	1	1	1	1	1	1	1	2
SAL0000923	group_2439	topology modulation protein	1	1	1	1	1	1	1	1	1	1	1	1	1	1	1	1	1	1	1	1	1	1	1	1	1	1	1	1	1	1	1	1	1	1	1	1	1	1	1	1	2
SAL0000924	divIB	Cell division protein DivIB	1	1	1	1	1	1	1	1	1	1	1	1	2	1	1	1	1	1	1	1	1	1	1	1	1	1	1	1	1	1	1	1	1	1	1	1	1	1	1	1	1
SAL0000925	group_2440	Enterocin A Immunity	1	1	2	1	1	1	1	1	1	1	1	1	1	1	2	1	1	1	1	1	1	1	1	1	1	1	1	1	1	2	1	2	1	1	1	1	1	1	1	1	3
SAL0000926	ycjP	Inner membrane ABC transporter permease protein YcjP	1	1	1	1	1	1	1	2	1	1	1	4	1	1	1	1	1	1	1	1	1	1	1	1	1	1	1	2	1	1	1	1	1	1	1	1	1	1	1	1	3
SAL0000927	hom	Homoserine dehydrogenase	1	1	1	1	1	1	1	1	1	1	1	1	1	1	1	1	1	1	1	1	1	1	1	1	1	1	1	1	1	1	1	1	1	1	1	1	1	1	1	1	2
SAL0000928	adaA	Bifunctional transcriptional activator/DNA repair enzyme AdaA	1	1	1	1	1	1	1	1	1	1	1	1	1	1	1	1	1	1	1	1	1	1	1	1	1	1	3	1	1	1	1	1	1	1	2	1	1	1	1	1	4
SAL0000929	phnE_2	Phosphate-import permease protein PhnE	1	1	2	1	1	1	1	1	1	1	1	1	1	1	2	1	1	1	1	1	1	1	1	1	1	1	1	1	1	2	1	2	1	1	1	1	1	1	1	1	1
SAL0000930	hp	hypothetical protein	2	2	2	2	2	2	2	2	2	2	2	2	2	2	2	2	2	2	2	2	2	2	2	2	2	2	2	2	2	2	2	2	2	2	2	2	2	2	2	2	3
SAL0000931	rnc	Ribonuclease 3	1	1	1	1	1	1	1	3	1	1	1	1	1	1	1	1	1	1	1	1	1	1	1	1	1	1	1	1	1	1	1	1	1	1	1	1	1	1	1	1	2
SAL0000932	hp	hypothetical protein	1	1	1	1	1	1	1	2	1	1	1	1	1	1	1	1	1	1	1	1	1	1	1	1	1	1	1	1	1	1	1	1	1	1	1	1	1	1	1	1	3
SAL0000933	citC	[Citrate [pro-3S]-lyase] ligase	1	1	1	1	1	1	1	1	2	1	1	1	1	1	1	2	2	2	2	1	1	2	2	2	2	2	1	1	1	1	1	1	1	1	2	1	1	2	2	1	3
SAL0000934	group_2449	chromosome segregation protein	1	1	2	1	1	1	1	3	1	1	1	5	1	1	2	1	1	1	1	1	1	1	1	1	1	1	1	3	1	2	1	2	1	1	1	1	1	1	1	1	4
SAL0000935	clpX	ATP-dependent Clp protease ATP-binding subunit ClpX	1	1	3	1	1	1	2	1	1	2	1	1	1	1	1	1	1	1	1	1	1	1	1	1	1	1	1	1	1	1	1	1	1	1	1	1	1	1	1	1	1
SAL0000936	group_2451	preprotein translocase subunit SecA	1	1	2	3	1	2	2	2	1	2	1	2	1	1	2	1	1	1	1	1	1	1	1	1	1	1	3	2	2	2	1	2	1	1	1	1	1	1	1	1	4
SAL0000937	group_2452	Carboxymuconolactone decarboxylase family protein	1	1	1	1	1	1	1	1	1	1	1	1	1	1	1	1	1	1	1	1	1	1	1	1	1	1	1	1	1	1	1	1	1	1	1	1	1	1	1	1	2
SAL0000938	glmU	Bifunctional protein GlmU	1	1	1	1	1	1	1	1	1	1	1	1	1	1	1	1	1	1	1	1	1	1	1	1	1	1	1	1	1	1	1	1	1	1	1	1	1	1	1	1	2
SAL0000939	mutS2	Endonuclease MutS2	1	1	1	1	1	1	1	1	1	1	1	1	1	1	1	1	1	1	1	1	1	1	1	1	1	1	1	1	1	1	1	1	1	1	1	1	1	1	1	1	2
SAL0000940	melR	Melibiose operon regulatory protein	1	1	1	1	1	3	1	1	1	1	1	1	1	1	1	1	1	1	1	1	1	1	1	1	1	1	1	1	1	1	1	1	1	1	1	1	1	1	1	1	2
SAL0000941	trmB	tRNA (guanine-N(7)-)-methyltransferase	1	1	1	1	1	1	1	2	1	1	1	1	1	1	1	1	1	1	1	1	1	1	1	1	1	1	1	1	1	1	1	1	1	1	1	1	1	1	1	1	3
SAL0000942	hp	hypothetical protein	1	1	1	1	1	1	1	1	1	2	1	1	1	1	1	1	1	1	1	1	1	1	1	1	1	1	1	1	1	1	1	1	1	1	1	1	1	1	1	1	3
SAL0000943	sigA	RNA polymerase sigma factor SigA	1	1	1	1	1	1	1	2	1	1	1	1	1	1	1	1	1	1	1	1	1	1	1	1	1	1	1	1	1	1	1	1	1	1	1	1	1	1	1	1	3
SAL0000944	group_2459	Uracil DNA glycosylase superfamily protein	1	1	2	1	1	1	1	1	1	1	1	1	1	1	2	1	1	1	1	1	1	1	1	1	1	1	1	1	1	2	1	2	1	1	1	1	1	1	1	1	3
SAL0000945	engB	putative GTP-binding protein EngB	1	1	1	1	1	1	1	3	1	1	1	4	1	1	1	1	1	1	1	1	1	1	1	1	1	1	1	1	1	1	1	1	1	1	1	1	1	1	1	1	2
SAL0000946	licC_3	Lichenan permease IIC component	1	1	1	1	1	1	1	1	1	1	1	1	1	1	1	1	1	1	1	1	1	1	1	1	1	1	1	3	1	1	1	1	1	1	2	1	1	1	1	1	1
SAL0000947	hp	hypothetical protein	1	1	1	1	1	1	1	1	1	1	1	1	1	1	1	1	1	1	1	1	1	1	1	1	1	1	1	1	1	1	1	1	1	1	1	1	1	1	1	1	2
SAL0000948	leuS	Leucine--tRNA ligase	1	1	1	1	1	1	1	3	1	1	1	1	1	1	2	1	1	1	1	1	1	1	1	1	1	1	1	3	5	2	1	2	1	1	1	1	1	1	1	1	4
SAL0000949	gsiC	Glutathione transport system permease protein GsiC	1	1	1	3	1	1	1	2	1	1	1	1	1	1	1	1	1	1	1	1	1	1	1	1	1	1	3	2	1	1	1	1	1	1	1	1	1	1	1	1	4
SAL0000950	adhE	Aldehyde-alcohol dehydrogenase	1	1	1	1	1	1	1	3	1	1	1	1	1	1	1	1	1	1	1	1	1	1	1	1	1	1	1	1	4	1	1	1	1	1	1	1	1	1	1	1	2
SAL0000951	tpx	putative thiol peroxidase	1	1	1	3	1	1	2	1	1	2	1	1	1	1	1	1	1	1	1	1	1	1	1	1	1	1	3	1	1	1	1	1	1	1	1	1	1	1	1	1	4
SAL0000952	hp	hypothetical protein	1	1	1	1	1	1	1	1	1	1	1	1	1	1	1	1	1	1	1	1	1	1	1	1	1	1	1	1	1	1	1	1	1	1	1	1	1	1	1	1	2

SAL0000953	rpsA	30S ribosomal protein S1	1	1	1	1	1	1	1	2	1	1	1	1	1	1	1	1	1	1	1	1	1	1	1	1	1	1	1	1	1	1	1	1	1	1	1	1	1	1	1	1	3
SAL0000954	pepQ	Xaa-Pro dipeptidase	1	1	1	1	1	1	1	1	1	1	1	1	1	1	1	1	1	1	1	1	1	1	1	1	1	1	1	1	1	1	1	1	1	1	1	1	1	1	1	1	2
SAL0000955	immR_1	HTH-type transcriptional regulator ImmR	1	1	1	1	1	1	1	1	1	1	1	1	1	1	1	1	1	1	1	1	1	1	1	1	1	1	1	1	1	1	1	1	1	1	1	1	1	1	1	1	2
SAL0000956	gatA	Glutamyl-tRNA(Gln) amidotransferase subunit A	1	1	1	3	1	1	1	2	1	1	1	1	1	1	4	1	1	1	1	1	1	1	1	1	1	1	3	2	1	1	1	1	1	1	1	1	1	1	1	1	5
SAL0000957	plsY_1	Glycerol-3-phosphate acyltransferase	1	1	1	1	1	1	1	3	1	1	1	1	1	1	1	1	1	1	1	1	1	1	1	1	1	1	1	1	1	1	1	1	1	1	1	1	1	1	1	1	2
SAL0000958	group_2472	glutamyl-tRNA(Gln) amidotransferase subunit E	1	1	1	1	1	1	1	1	1	1	1	1	1	1	2	1	1	1	1	1	1	1	1	1	1	1	1	1	1	1	1	1	1	1	1	1	1	1	1	1	1
SAL0000959	emrB	Multidrug export protein EmrB	1	1	3	1	1	1	1	2	1	1	1	1	1	1	3	1	1	1	1	1	1	1	1	1	1	1	1	2	6	4	1	4	1	1	1	1	1	1	1	1	5
SAL0000960	ffh	Signal recognition particle protein	1	1	1	1	1	1	1	2	1	1	1	1	1	1	1	1	1	1	1	1	1	1	1	1	1	1	1	1	1	1	1	1	1	1	1	1	1	1	1	1	1
SAL0000961	purD	Phosphoribosylamine--glycine ligase	1	1	2	1	1	1	1	2	1	1	1	1	1	1	2	1	1	1	1	1	1	1	1	1	1	1	1	2	1	2	1	2	1	1	1	1	1	1	1	1	1
SAL0000962	fmt	Methionyl-tRNA formyltransferase	1	1	1	1	1	3	1	1	1	1	1	1	1	1	1	1	1	1	1	1	1	1	1	1	1	1	1	1	1	1	1	1	1	1	1	1	1	1	1	1	2
SAL0000963	lacL	Beta-galactosidase large subunit	1	1	1	1	1	1	1	2	1	1	1	1	1	1	1	1	1	1	1	1	1	1	1	1	1	1	1	2	4	1	1	1	1	1	1	1	1	1	1	1	3
SAL0000964	endA_2	DNA-entry nuclease	1	1	1	1	1	1	1	1	1	1	1	1	1	1	1	1	1	1	1	1	1	1	1	1	1	1	1	1	1	1	1	1	1	1	1	1	1	1	1	1	2
SAL0000965	gspA_2	General stress protein A	1	1	1	1	1	1	1	1	1	1	1	1	1	1	1	1	1	1	1	1	1	1	1	1	1	1	1	1	1	1	1	1	1	1	1	1	1	1	1	1	2
SAL0000966	yclM_1	Aspartokinase 3	1	1	1	1	1	1	1	2	1	1	1	1	1	1	1	1	1	1	1	1	1	1	1	1	1	1	1	1	4	1	1	1	1	1	1	1	1	1	1	1	3
SAL0000967	gspA_1	General stress protein A	1	1	1	1	1	1	1	1	1	1	1	3	1	1	1	1	1	1	1	1	1	1	1	1	1	1	1	1	1	1	1	1	1	1	1	1	1	1	1	1	2
SAL0000968	group_2481	Putative multidrug export ATP-binding/permease protein	1	1	2	1	1	1	1	3	1	1	1	4	1	1	2	1	1	1	1	1	1	1	5	1	1	1	1	3	1	2	1	2	1	1	1	1	1	1	1	1	6
SAL0000969	group_2482	Serine/threonine exchanger SteT	1	1	1	1	1	1	1	2	1	1	1	1	1	1	1	1	1	1	1	1	1	1	1	1	1	1	1	3	1	1	1	1	1	1	1	1	1	1	1	1	4
SAL0000970	hp	hypothetical protein	1	1	1	1	1	1	1	1	1	1	1	1	1	1	1	1	1	1	1	1	1	1	1	1	1	1	1	2	1	1	1	1	1	1	1	1	1	1	1	1	1
SAL0000971	dtd	D-tyrosyl-tRNA(Tyr) deacylase	1	1	1	1	1	1	1	1	1	1	1	1	1	1	1	1	1	1	1	1	1	1	1	1	1	1	1	1	1	1	1	1	1	1	1	1	1	1	1	1	2
SAL0000972	trmK	tRNA (adenine(22)-N(1))-methyltransferase	1	1	1	1	1	1	1	2	1	1	1	1	1	1	1	1	1	1	1	1	1	1	1	1	1	1	1	1	1	1	1	1	1	1	1	1	1	1	1	1	3
SAL0000973	hp	hypothetical protein	1	1	1	1	1	1	1	1	1	1	1	1	1	1	1	1	1	1	1	1	1	1	1	1	1	1	1	1	1	1	1	1	1	1	1	1	1	1	1	1	2
SAL0000974	pyrH	Uridylate kinase	1	1	2	1	1	3	3	2	1	3	1	3	1	1	2	1	1	1	1	1	1	1	1	1	1	1	1	2	3	2	1	2	1	1	1	1	1	1	1	1	4
SAL0000975	group_2488	Na( )-translocating NADH-quinone reductase subunit C	1	1	1	1	1	1	1	1	1	1	1	1	1	1	1	1	1	1	1	1	1	1	1	1	1	1	1	1	1	1	1	1	1	1	1	1	1	1	1	1	2
SAL0000976	hp	hypothetical protein	1	1	1	1	1	1	1	1	1	1	1	1	1	1	1	1	1	1	1	1	1	1	1	1	1	1	1	1	1	1	1	1	1	1	1	1	1	1	1	1	2
SAL0000977	group_2490	anaerobic ribonucleotide reductase-activating protein	1	1	1	1	1	1	1	1	1	1	1	1	1	1	1	1	1	1	1	1	1	1	1	1	1	1	1	1	1	1	1	1	1	1	1	1	1	1	1	1	2
SAL0000978	group_2491	ribosome-associated heat shock protein Hsp15	1	1	1	1	1	1	1	1	1	1	1	1	1	1	1	1	1	1	1	1	1	1	1	1	1	1	1	1	1	1	1	1	1	1	1	1	1	1	1	1	2
SAL0000979	cshA_2	DEAD-box ATP-dependent RNA helicase CshA	1	1	2	1	1	1	1	1	1	1	1	1	1	1	2	1	1	1	1	1	1	1	1	1	1	1	1	1	3	2	1	2	1	1	1	1	1	1	1	1	4
SAL0000980	hp	hypothetical protein	1	1	1	1	1	1	1	1	1	1	1	1	1	1	1	1	1	1	1	1	1	1	1	1	1	1	1	1	1	1	1	1	1	1	1	2	1	1	1	1	3
SAL0000981	hp	hypothetical protein	1	1	1	1	1	1	1	1	1	1	1	1	1	1	1	1	1	1	1	1	1	1	1	1	1	1	1	1	1	1	1	1	1	1	1	1	1	1	1	1	2
SAL0000982	group_2495	Major Facilitator Superfamily protein	1	1	1	1	1	1	1	2	1	1	1	1	1	1	1	1	1	1	1	1	1	1	1	1	1	1	1	2	1	1	1	1	1	1	1	1	1	1	1	1	1
SAL0000983	group_2496	Putative TrmH family tRNA/rRNA methyltransferase	1	1	1	1	1	1	1	1	1	1	1	1	1	1	2	1	1	1	1	1	1	1	1	1	1	1	1	1	1	2	1	2	1	1	1	3	1	1	1	1	4
SAL0000984	hp	hypothetical protein	1	1	1	1	1	1	1	1	1	1	1	1	1	1	1	1	1	1	1	1	1	1	1	1	1	1	1	1	1	1	1	1	1	1	1	1	1	1	1	1	2
SAL0000985	yycI	Two-component system YycFG regulatory protein	1	1	1	1	1	1	1	1	2	1	1	1	1	1	1	2	2	2	2	1	1	2	2	2	2	2	1	1	3	1	1	1	1	1	2	1	1	2	2	1	4
SAL0000986	pyrE	Orotate phosphoribosyltransferase	1	1	1	2	1	1	1	1	1	1	1	1	1	1	1	1	1	1	1	1	1	1	1	1	1	1	2	1	1	1	1	1	1	1	1	1	1	1	1	1	3
SAL0000987	lolD	Lipoprotein-releasing system ATP-binding protein LolD	2	2	2	2	2	2	2	2	2	2	2	2	2	2	2	2	2	2	2	2	2	2	2	2	2	2	2	2	2	2	2	2	2	2	2	2	2	2	2	2	3
SAL0000988	carA_2	Carbamoyl-phosphate synthase small chain	1	1	1	1	1	1	1	1	1	1	1	1	1	1	1	1	1	1	1	1	1	1	1	1	1	1	1	1	1	1	1	1	1	1	1	1	1	1	1	1	2
SAL0000989	hp	hypothetical protein	1	1	4	3	1	1	2	5	1	2	1	1	1	1	1	1	1	1	1	1	1	1	1	1	1	1	3	1	1	1	1	1	1	1	1	1	1	1	1	1	6
SAL0000990	fni	Isopentenyl-diphosphate delta-isomerase	1	1	3	1	1	1	1	1	1	1	1	1	1	1	1	1	1	1	1	1	1	1	1	1	1	1	1	1	1	1	1	1	1	1	1	1	1	1	1	1	2
SAL0000991	nudF	ADP-ribose pyrophosphatase	1	1	3	1	1	2	2	3	1	2	1	2	1	1	3	1	1	1	1	1	1	1	1	1	1	1	1	3	2	3	1	3	1	1	1	1	1	1	1	1	2
SAL0000992	group_2505	putative acyltransferase	1	1	1	1	1	1	1	1	1	1	1	1	1	1	1	1	1	1	1	1	1	1	1	1	1	1	1	1	1	1	1	1	1	1	1	1	1	1	1	1	2
SAL0000993	btr_1	HTH-type transcriptional activator Btr	1	1	1	1	1	1	1	1	1	1	1	1	1	1	1	1	1	1	1	1	1	1	1	1	1	1	1	1	1	1	1	1	1	1	1	1	1	1	1	1	2
SAL0000994	hp	hypothetical protein	1	1	4	3	1	1	1	2	1	1	1	1	1	1	2	1	1	1	1	1	1	1	1	1	1	1	3	2	1	2	1	2	1	1	1	1	1	1	1	1	1
SAL0000995	hp	hypothetical protein	1	1	1	1	1	1	1	1	1	1	1	1	1	1	1	1	1	1	1	1	1	1	1	1	1	1	1	1	1	1	1	1	1	1	1	1	1	1	1	1	2
SAL0000996	lagD_2	Lactococcin-G-processing and transport ATP-binding protein LagD	1	1	1	1	1	1	1	1	1	1	1	3	1	1	1	1	1	1	1	1	1	1	1	1	1	1	1	1	1	1	1	1	1	1	1	1	1	1	1	1	2
SAL0000997	hp	hypothetical protein	1	1	1	1	1	1	1	1	1	1	1	1	1	1	1	1	1	1	1	1	1	1	1	1	1	1	1	1	1	1	1	1	1	1	1	1	1	1	1	1	2
SAL0000998	prkC	Serine/threonine-protein kinase PrkC	1	1	1	1	1	1	2	1	1	2	1	3	1	1	1	1	1	1	1	1	1	1	1	1	1	1	1	1	1	1	1	1	1	1	1	1	1	1	1	1	4
SAL0000999	group_2512	NADH dehydrogenase-like protein	1	1	1	1	1	1	1	1	1	1	2	1	1	1	1	1	1	1	1	2	2	1	1	1	1	1	1	1	1	1	2	1	1	1	1	1	1	1	1	1	3
SAL0001000	group_2513	CBS domain protein	1	1	4	2	1	2	2	2	1	2	1	2	1	1	3	1	1	1	1	1	1	1	1	1	1	1	2	2	2	3	1	3	1	1	1	1	1	1	5	1	2
SAL0001001	hp	hypothetical protein	1	1	1	1	1	1	1	1	1	1	1	1	1	1	1	1	1	1	1	1	1	1	1	1	1	1	1	1	1	1	1	1	1	1	1	1	1	1	1	1	2
SAL0001002	group_2515	Membrane transport protein	1	1	1	1	1	1	1	1	1	1	1	1	1	1	1	1	1	1	1	1	1	1	1	1	1	1	1	1	1	1	1	1	1	1	1	1	1	1	1	1	2
SAL0001003	hp	hypothetical protein	1	1	1	1	1	1	1	1	1	1	1	2	1	1	1	1	1	1	1	1	1	1	1	1	1	1	1	1	1	1	1	1	1	1	1	1	1	1	1	1	3
SAL0001004	hp	hypothetical protein	1	1	1	1	1	1	1	1	1	1	1	1	1	1	1	1	1	1	1	1	1	1	1	1	1	1	1	1	1	1	1	1	1	1	1	1	1	1	1	1	2
SAL0001005	hp	hypothetical protein	1	1	1	1	1	1	1	1	1	1	1	1	1	1	1	1	1	1	1	1	1	1	1	1	1	1	1	1	1	1	1	1	1	1	1	1	1	1	1	1	2
SAL0001006	pepD	Dipeptidase	1	1	1	1	1	1	1	4	1	1	1	1	1	1	1	1	1	1	1	1	1	1	1	1	1	1	1	3	1	1	1	1	1	1	1	1	1	1	1	1	2
SAL0001007	group_2520	site-specific tyrosine recombinase XerC	1	1	1	1	1	1	1	1	1	1	1	1	1	1	1	1	1	1	1	1	1	1	1	1	1	1	1	1	1	1	1	1	1	1	1	1	1	1	1	1	2
SAL0001008	rpmE2	50S ribosomal protein L31 type B	1	1	1	1	1	1	1	2	1	1	1	1	1	1	1	1	1	1	1	1	1	1	1	1	1	1	1	1	1	1	1	1	1	1	1	1	1	1	1	1	1
SAL0001009	hp	hypothetical protein	1	1	1	1	1	1	1	1	1	1	1	1	1	1	1	1	1	1	1	1	1	1	1	1	1	1	1	1	1	1	1	1	1	1	1	1	1	1	1	1	2
SAL0001010	acyP	Acylphosphatase	1	1	1	1	1	1	1	1	1	1	1	1	1	1	1	1	1	1	1	1	1	1	1	1	1	1	1	1	1	1	1	1	1	1	1	1	1	1	1	1	2
SAL0001011	pepF1	Oligoendopeptidase F, plasmid	1	1	1	2	1	1	1	1	1	1	1	1	1	1	3	1	1	1	1	1	1	1	1	1	1	1	2	1	1	1	1	1	1	1	1	1	1	1	1	1	4
SAL0001012	nfrA1	FMN reductase (NADPH)	1	1	1	1	1	1	1	2	1	1	1	1	1	1	1	1	1	1	1	1	1	1	1	1	1	1	1	1	1	1	1	1	1	1	1	1	1	1	1	1	3
SAL0001013	group_2526	Beta-lactamase superfamily domain protein	1	1	1	1	1	1	1	1	1	1	1	1	1	1	1	1	1	1	1	1	1	1	1	1	1	1	1	1	1	1	1	1	1	1	1	1	1	1	1	1	2
SAL0001014	hp	hypothetical protein	1	1	1	1	1	1	1	1	1	1	1	2	1	1	1	1	1	1	1	1	1	1	1	1	1	1	1	1	1	1	1	1	1	1	1	1	1	1	1	1	1
SAL0001015	glnH_1	ABC transporter glutamine-binding protein GlnH precursor	1	1	1	1	1	1	1	1	1	1	1	1	1	1	1	1	1	1	1	1	1	1	1	1	1	1	1	1	1	1	1	1	1	1	1	1	1	1	1	1	2
SAL0001016	ybjG	Putative undecaprenyl-diphosphatase YbjG	1	1	1	1	1	1	1	1	1	1	1	1	1	1	1	1	1	1	1	1	1	1	1	1	1	1	1	1	1	1	1	1	1	1	1	1	1	1	1	1	2

SAL0001017	hp	hypothetical protein	1	1	1	2	1	1	1	3	1	1	1	1	1	1	1	1	1	1	1	1	1	1	1	1	1	1	2	1	1	1	1	1	1	1	1	1	1	1	1	1	4
SAL0001018	hp	hypothetical protein	1	1	1	1	1	1	2	1	1	2	1	1	1	1	1	1	1	1	1	1	1	1	1	1	1	1	1	1	1	1	1	1	1	1	1	1	1	1	1	1	1
SAL0001019	group_2532	NADH oxidase	1	1	1	1	1	1	2	1	1	2	1	1	1	1	1	1	1	1	1	1	1	1	1	1	1	1	1	1	1	1	1	1	1	1	1	1	1	1	1	1	3
SAL0001020	hp	hypothetical protein	1	1	1	1	1	1	1	4	1	1	1	1	1	1	1	1	1	1	1	1	1	1	1	1	1	1	1	1	3	1	1	1	1	1	1	1	1	1	1	1	2
SAL0001021	soxS	Regulatory protein SoxS	1	1	1	1	1	1	1	1	1	1	1	1	1	1	1	1	1	1	1	1	1	1	1	1	1	1	1	1	1	1	1	1	1	1	1	1	1	1	1	1	2
SAL0001022	hp	hypothetical protein	1	1	1	1	1	1	1	2	1	1	1	1	1	1	1	1	1	1	1	1	1	1	1	1	1	1	1	1	1	1	1	1	1	1	1	1	1	1	1	1	3
SAL0001023	glcU_1	putative glucose uptake protein GlcU	1	1	1	1	1	1	1	1	1	1	1	4	1	1	1	1	1	1	1	1	1	1	1	1	1	1	1	1	1	2	1	2	1	1	1	1	1	1	1	1	3
SAL0001024	xylB	Xylulose kinase	1	1	2	1	1	1	1	2	1	1	1	1	1	1	2	1	1	1	1	1	1	1	1	1	1	1	1	2	1	2	1	2	1	1	1	1	1	1	1	1	3
SAL0001025	dkgA_1	2,5-diketo-D-gluconic acid reductase A	1	1	1	1	1	1	1	3	1	1	1	1	1	1	1	1	1	1	1	1	1	1	1	1	1	1	1	1	1	1	1	1	1	1	1	1	1	1	1	1	2
SAL0001026	hp	hypothetical protein	1	1	1	1	1	1	1	3	1	1	1	1	1	1	1	1	1	1	1	1	1	1	1	1	1	1	1	1	1	1	1	1	1	1	1	1	1	1	1	1	2
SAL0001027	group_254	Helix-turn-helix domain protein	1	1	1	1	1	1	1	1	1	1	1	2	1	1	1	1	1	1	1	1	1	1	1	1	1	1	1	1	1	1	1	1	1	1	1	1	1	1	1	1	3
SAL0001028	group_2540	MucBP domain protein	1	1	1	1	1	1	1	1	1	1	1	1	1	1	1	1	1	1	1	1	1	1	1	1	1	1	1	1	1	1	1	1	1	1	1	1	1	1	1	1	2
SAL0001029	group_2541	HIT-like protein	1	1	1	1	1	1	1	1	1	1	1	1	1	1	1	1	1	1	1	1	1	1	1	1	1	1	1	1	1	1	1	1	1	1	1	1	1	1	1	1	2
SAL0001030	group_2542	YycH protein	1	1	2	1	1	1	1	1	1	1	1	1	1	1	2	1	1	1	1	1	1	1	1	1	1	1	1	1	1	2	1	2	1	1	1	1	1	1	1	1	3
SAL0001031	ybjI_3	Flavin mononucleotide phosphatase YbjI	1	1	1	1	1	1	1	1	1	1	1	1	1	1	1	1	1	1	1	1	1	1	1	1	1	1	1	1	1	1	1	1	1	1	1	1	1	1	1	1	2
SAL0001032	group_2544	Major Facilitator Superfamily protein	1	1	1	1	1	1	1	1	1	1	1	1	1	1	1	1	1	1	1	1	1	1	1	1	1	1	1	1	1	1	1	1	1	1	1	1	1	1	1	1	2
SAL0001033	rsmB	Ribosomal RNA small subunit methyltransferase B	1	1	3	1	1	1	1	1	1	1	1	1	1	1	2	1	1	1	1	1	1	1	1	1	1	1	1	1	1	2	1	2	1	1	1	1	1	1	1	1	4
SAL0001034	group_2546	Bacterial low temperature requirement A protein (LtrA)	1	1	1	1	1	3	1	1	1	1	1	1	1	1	1	1	1	1	1	1	1	1	1	1	1	1	1	1	1	1	1	1	1	1	1	1	1	1	1	1	2
SAL0001035	hp	hypothetical protein	1	1	1	1	1	1	1	1	1	1	1	1	1	1	1	1	1	1	1	1	1	1	1	1	1	1	1	1	1	1	1	1	1	1	1	1	1	1	1	1	2
SAL0001036	hp	hypothetical protein	1	1	1	1	1	1	1	1	1	1	1	1	1	1	1	1	1	1	1	1	1	1	1	1	1	1	1	1	1	1	1	1	1	1	1	1	1	1	1	1	2
SAL0001037	ytlR	Putative lipid kinase YtlR	1	1	1	1	1	1	2	1	1	2	1	1	1	1	1	1	1	1	1	1	1	1	1	1	1	1	1	1	1	1	1	1	1	1	1	1	1	1	1	1	1
SAL0001038	ycfH	putative deoxyribonuclease YcfH	1	1	1	1	1	1	1	1	1	1	1	1	1	1	1	1	1	1	1	1	1	1	1	1	1	1	2	1	1	1	1	1	1	1	1	1	1	1	1	1	3
SAL0001039	group_2551	DAK2 domain protein	1	1	1	1	1	2	1	1	1	1	1	1	1	1	1	1	1	1	1	1	1	1	1	1	1	1	1	1	1	1	1	1	1	1	1	1	1	1	1	1	3
SAL0001040	gmuC_1	Oligo-beta-mannoside permease IIC component	1	1	1	2	1	1	1	1	1	1	1	1	1	1	1	1	1	1	1	1	1	1	1	1	1	1	2	1	1	1	1	1	1	1	1	1	1	1	1	1	3
SAL0001041	hp	hypothetical protein	1	1	1	1	1	1	1	1	1	1	1	1	1	1	1	1	1	1	1	1	1	1	1	1	1	1	1	1	1	1	1	1	1	1	1	1	1	1	1	1	2
SAL0001042	group_2554	Colicin V production protein	1	1	1	1	1	1	1	1	1	1	1	1	1	1	1	1	1	1	1	1	1	1	1	1	1	1	1	1	1	1	1	1	1	1	1	1	1	1	1	1	2
SAL0001043	pbpX_2	Putative penicillin-binding protein PbpX	1	1	1	1	1	1	1	1	1	1	1	1	1	1	1	1	1	1	1	1	1	1	1	1	1	1	1	1	1	1	1	1	1	1	1	1	1	1	1	1	2
SAL0001044	galK	Galactokinase	1	1	1	4	1	1	3	1	1	3	1	1	1	1	2	1	1	1	1	1	1	1	1	1	1	1	4	1	1	2	1	2	1	1	1	1	1	1	1	1	5
SAL0001045	atl	Bifunctional autolysin precursor	1	1	1	1	1	1	1	3	1	1	1	1	1	1	1	1	1	1	1	1	1	1	1	1	1	1	1	1	1	1	1	1	1	1	1	1	1	1	1	1	2
SAL0001046	hp	hypothetical protein	1	1	1	1	1	1	1	2	1	1	1	1	1	1	1	1	1	1	1	1	1	1	1	1	1	1	1	1	1	1	1	1	1	1	1	1	1	1	1	1	1
SAL0001047	group_2559	LysR substrate binding domain protein	1	1	1	1	1	1	1	3	1	1	1	1	1	1	1	1	1	1	1	1	1	1	1	1	1	1	1	1	1	1	1	1	1	1	1	1	1	1	1	1	2
SAL0001048	metQ_1	putative D-methionine-binding lipoprotein MetQ precursor	1	1	1	1	1	1	1	1	1	1	1	1	1	1	1	1	1	1	1	1	1	1	1	1	1	1	1	1	1	1	1	1	1	1	1	1	1	1	1	1	2
SAL0001049	ymdB	O-acetyl-ADP-ribose deacetylase	1	1	2	1	1	1	1	4	1	1	1	1	1	1	2	1	1	1	1	1	1	1	1	1	1	1	1	1	1	2	1	2	1	1	1	1	1	1	1	1	3
SAL0001050	hp	hypothetical protein	1	1	1	1	1	1	1	1	1	1	1	1	1	1	2	1	1	1	1	1	1	1	1	1	1	1	1	1	1	2	1	2	1	1	1	1	1	1	1	1	3
SAL0001051	hp	hypothetical protein	1	1	1	1	1	1	1	1	1	1	1	1	1	1	1	1	1	1	1	1	1	1	1	1	1	1	1	1	1	1	1	1	1	1	1	1	1	1	1	1	2
SAL0001052	yclM_2	Aspartokinase 3	1	1	1	1	1	1	1	1	1	1	1	1	1	1	1	1	1	1	1	1	1	1	1	1	1	1	1	1	1	1	1	1	1	1	1	1	1	1	1	1	2
SAL0001053	hp	hypothetical protein	1	1	1	1	1	1	1	3	1	1	1	1	1	1	1	1	1	1	1	1	1	1	1	1	1	1	1	1	1	1	1	1	1	1	1	1	1	1	1	1	2
SAL0001054	lptB	Lipopolysaccharide export system ATP-binding protein LptB	1	1	1	1	1	1	1	1	1	1	1	1	1	1	1	1	1	1	1	1	1	1	1	1	1	1	1	1	1	1	1	1	1	1	1	1	1	1	1	1	2
SAL0001055	rihC_2	Non-specific ribonucleoside hydrolase RihC	1	1	1	1	1	1	1	1	1	1	1	1	1	1	1	1	1	1	1	1	1	1	1	1	1	1	1	1	1	1	1	1	1	1	1	1	1	1	1	1	2
SAL0001056	group_2568	flavodoxin	1	1	1	1	1	1	1	1	1	1	1	1	1	1	1	1	1	1	1	1	1	1	1	1	1	1	1	1	1	1	1	1	1	1	1	1	1	1	1	1	2
SAL0001057	yvdM	Beta-phosphoglucomutase	1	1	1	1	1	1	1	1	1	1	1	1	1	1	1	1	1	1	1	1	1	1	1	1	1	1	1	1	1	1	1	1	1	1	1	1	1	1	1	1	2
SAL0001058	hp	hypothetical protein	1	1	1	1	1	1	1	1	1	1	1	3	1	1	1	1	1	1	1	1	1	1	1	1	1	1	1	1	1	1	1	1	1	1	1	1	1	1	1	1	2
SAL0001059	hp	hypothetical protein	1	1	1	1	1	1	1	1	1	1	1	1	1	1	1	1	1	1	1	1	1	1	1	1	1	1	1	1	1	1	1	1	1	1	1	1	1	1	1	1	2
SAL0001060	trxA_2	Thioredoxin	1	1	1	1	1	1	1	1	1	1	1	1	1	1	1	1	1	1	1	1	1	1	1	1	1	1	1	1	1	1	1	1	1	1	1	1	1	1	1	1	2
**SAL0001061**	**frdA**	**Fumarate reductase flavoprotein subunit**	3	1	4	1	1	1	2	1	1	2	1	1	1	1	1	1	1	1	1	1	1	1	1	1	1	1	1	1	1	1	1	1	1	1	1	1	1	1	1	1	5
SAL0001062	hp	hypothetical protein	1	1	1	1	1	1	1	1	1	1	1	1	1	1	1	1	1	1	1	1	1	1	1	1	1	1	1	1	2	1	1	1	1	1	1	1	1	1	1	1	3
SAL0001063	pepT_2	Peptidase T	1	1	1	1	1	1	1	1	1	1	1	1	1	1	1	1	1	1	1	1	1	1	1	1	1	1	1	1	1	1	1	1	1	1	1	1	1	1	1	1	2
SAL0001064	hp	hypothetical protein	1	1	1	1	1	1	1	1	1	1	1	1	1	1	1	1	1	1	1	1	1	1	1	1	1	1	1	1	1	1	1	1	1	1	1	1	1	1	1	1	2
SAL0001065	group_2577	camphor resistance protein CrcB	1	1	1	1	1	1	1	1	1	1	1	1	1	1	1	1	1	1	1	1	1	1	1	1	1	1	1	1	1	1	1	1	1	1	1	1	1	1	1	1	2
SAL0001066	yhfK_1	putative sugar epimerase YhfK	1	1	1	1	1	1	1	1	1	1	1	1	1	1	1	1	1	1	1	1	1	1	1	1	1	1	1	1	1	1	1	1	1	1	1	1	1	1	1	1	2
SAL0001067	group_2579	Acyltransferase	1	1	2	1	1	1	1	2	1	1	1	1	1	1	2	1	1	1	1	1	1	1	1	1	1	1	1	2	1	2	1	2	1	1	1	1	1	1	1	1	3
SAL0001068	pyrB	Aspartate carbamoyltransferase	1	1	1	1	1	1	1	1	1	1	1	1	1	1	1	1	1	1	1	1	1	1	1	1	1	1	1	1	1	1	1	1	1	1	1	1	1	1	1	1	2
SAL0001069	rpe	Ribulose-phosphate 3-epimerase	1	1	1	1	1	1	1	1	1	1	1	1	1	1	1	1	1	1	1	1	1	1	1	1	1	1	1	1	1	1	1	1	1	1	1	1	1	1	1	1	2
SAL0001070	hp	hypothetical protein	1	1	1	1	1	1	1	1	1	1	1	1	1	1	1	1	1	1	1	1	1	1	1	1	1	1	1	1	1	1	1	1	1	1	1	1	1	1	1	1	2
SAL0001071	walK_1	Sensor protein kinase WalK	1	1	3	8	1	2	2	5	1	2	1	2	1	1	7	1	1	1	1	1	1	1	1	1	1	1	4	3	2	3	1	3	1	1	1	1	1	1	1	1	6
SAL0001072	group_2584	putative racemase	1	1	1	1	1	1	1	1	1	1	1	2	1	1	1	1	1	1	1	1	1	1	1	1	1	1	1	1	1	1	1	1	1	1	1	1	1	1	1	1	3
SAL0001073	ywqE	Tyrosine-protein phosphatase YwqE	1	1	1	2	1	1	1	1	1	1	1	1	1	1	1	1	1	1	1	1	1	1	1	1	1	1	2	1	4	1	1	1	1	1	1	1	1	1	1	1	3
SAL0001074	hsrA_2	putative transport protein HsrA	1	1	2	1	1	1	1	1	1	1	1	1	1	1	1	1	1	1	1	1	1	1	1	1	1	1	1	1	1	1	1	1	1	1	1	1	1	1	1	1	3
SAL0001075	hp	hypothetical protein	1	1	2	1	1	1	1	2	1	1	1	3	1	1	2	1	1	1	1	1	1	1	1	1	1	1	1	2	1	2	1	2	1	1	1	1	1	1	1	1	4
SAL0001076	hp	hypothetical protein	1	1	1	1	1	1	1	1	1	1	1	1	1	1	1	1	1	1	1	1	1	1	1	1	1	1	1	1	1	1	1	1	1	1	1	1	1	1	1	1	2
SAL0001077	hp	hypothetical protein	1	1	1	1	1	1	1	1	1	1	1	1	2	1	1	1	1	1	1	1	1	1	1	1	1	1	1	1	1	1	1	1	1	1	1	1	1	1	1	1	3
SAL0001078	ftsL_1	Cell division protein FtsL	1	1	1	1	1	1	1	3	1	1	1	1	1	1	1	1	1	1	1	1	1	1	1	1	1	1	1	1	1	1	1	1	1	1	1	1	1	1	1	1	2
SAL0001079	dnaI	Primosomal protein DnaI	1	1	5	1	1	1	1	2	1	3	1	1	1	1	1	1	1	1	1	1	1	1	1	1	1	1	1	1	1	1	1	1	1	1	1	1	1	1	1	1	4
SAL0001080	recJ	Single-stranded-DNA-specific exonuclease RecJ	1	1	2	1	1	1	1	1	1	1	1	1	1	1	1	1	1	1	1	1	1	1	1	1	1	1	1	1	1	1	1	1	1	1	1	1	1	1	1	1	3

SAL0001081	hp	hypothetical protein	1	1	1	1	1	1	1	1	1	1	1	1	1	1	1	1	1	1	1	1	1	1	1	1	1	1	1	1	1	1	1	1	1	1	1	1	1	1	1	2	3
SAL0001082	rpsU	30S ribosomal protein S21	1	1	1	1	1	1	2	1	1	2	1	1	1	1	1	1	1	1	1	1	1	1	1	1	1	1	1	1	1	1	1	1	1	1	1	1	1	1	1	1	1
SAL0001083	rnhB	Ribonuclease HII	1	1	1	1	1	1	1	1	1	1	1	1	1	1	1	1	1	1	1	1	1	1	1	1	1	1	1	1	1	1	1	1	1	1	1	1	1	1	1	1	2
SAL0001084	polC_3	DNA polymerase III PolC-type	1	1	1	1	1	1	1	2	1	1	1	1	1	1	1	1	1	1	1	1	1	1	1	1	1	1	1	1	1	1	1	1	1	1	1	1	1	1	1	1	3
SAL0001085	hp	hypothetical protein	1	1	1	1	1	1	1	1	1	1	1	1	1	1	1	1	1	1	1	1	1	1	1	1	1	1	1	1	1	1	1	1	1	1	1	1	1	1	1	1	2
SAL0001086	yvoA_2	HTH-type transcriptional repressor YvoA	1	1	1	1	1	1	1	5	1	1	1	4	1	1	1	1	1	1	1	1	1	1	1	1	1	1	1	1	1	1	1	1	1	1	1	1	1	1	1	1	3
SAL0001087	rpmB	50S ribosomal protein L28	1	1	1	1	1	1	1	1	1	1	1	1	1	1	1	1	1	1	1	1	1	1	1	1	1	1	1	1	1	1	1	1	1	1	1	1	1	1	1	1	2
SAL0001088	metP	Methionine import system permease protein MetP	1	1	2	1	1	3	1	1	1	1	1	1	1	1	2	1	1	1	1	1	1	1	1	1	1	1	1	1	5	2	1	2	1	1	1	1	1	1	1	1	4
SAL0001089	hp	hypothetical protein	1	1	1	1	1	1	1	1	1	1	1	2	1	1	1	1	1	1	1	1	1	1	1	1	1	1	1	1	1	1	1	1	1	1	1	1	1	1	1	1	1
SAL0001090	mscL	Large-conductance mechanosensitive channel	1	1	1	2	1	1	1	4	1	1	1	1	1	1	1	1	1	1	1	1	1	1	1	1	1	1	2	1	1	1	1	1	1	1	1	3	1	1	1	1	5
SAL0001091	lysA	Diaminopimelate decarboxylase	1	1	1	1	1	1	1	2	1	1	1	1	1	1	1	1	1	1	1	1	1	1	1	1	1	1	1	1	1	1	1	1	1	1	1	1	1	1	1	1	1
SAL0001092	lytR_3	Transcriptional regulator LytR	1	1	1	1	1	1	1	1	1	1	1	1	1	1	1	1	1	1	1	1	1	1	1	1	1	1	1	1	3	1	1	1	1	1	1	1	1	1	1	1	2
SAL0001093	rpsK	30S ribosomal protein S11	1	1	2	1	1	2	2	2	1	2	1	3	1	1	2	1	1	1	1	1	1	1	1	1	1	1	1	2	2	2	1	2	1	1	1	1	1	1	1	1	2
SAL0001094	group_2605	HNH endonuclease	1	1	1	1	1	1	1	2	1	1	1	1	1	1	1	1	1	1	1	1	1	1	1	1	1	1	1	1	1	1	1	1	1	1	1	1	1	1	1	1	3
SAL0001095	ybaK	Cys-tRNA(Pro)/Cys-tRNA(Cys) deacylase YbaK	1	1	1	2	1	1	3	2	1	4	1	2	1	1	1	1	1	1	1	1	1	1	1	1	1	1	2	1	1	1	1	1	1	1	1	1	1	1	1	1	5
SAL0001096	nhaK	Sodium, potassium, lithium and rubidium/H( ) antiporter	1	1	1	2	1	1	1	1	1	1	1	1	1	1	4	1	1	1	1	1	1	1	1	1	1	1	2	1	3	1	1	1	1	1	1	1	1	1	1	1	5
SAL0001097	chbC_1	N,N'-diacetylchitobiose permease IIC component	1	1	1	1	1	4	1	1	1	1	1	1	1	1	2	1	1	1	1	1	1	1	1	1	1	1	1	1	1	2	1	2	1	1	1	1	1	1	1	1	3
SAL0001098	odcI	Ornithine decarboxylase, inducible	1	1	2	1	1	1	1	3	1	1	1	1	1	1	1	1	1	1	1	1	1	1	1	1	1	1	1	1	1	1	1	1	1	1	1	1	1	1	1	1	4
SAL0001099	group_270	pyridoxamine kinase	1	1	1	1	1	1	1	2	1	1	1	4	1	1	1	1	1	1	1	1	1	1	1	1	1	1	1	1	1	1	1	1	1	1	1	1	1	1	1	1	3
SAL0001100	bmrR	Multidrug-efflux transporter 1 regulator	1	1	2	1	1	2	2	4	1	2	1	5	1	1	3	1	1	1	1	1	1	1	1	1	1	1	1	4	2	3	1	3	1	1	1	1	1	1	1	1	6
SAL0001101	oxyR	Hydrogen peroxide-inducible genes activator	1	1	1	1	1	1	1	1	1	1	1	1	1	1	1	1	1	1	1	1	1	1	1	1	1	1	1	1	1	1	1	1	1	1	1	1	1	1	1	1	2
SAL0001102	steT_2	Serine/threonine exchanger SteT	1	1	1	2	1	1	1	3	1	1	1	1	1	1	1	1	1	1	1	1	1	1	1	1	1	1	2	1	1	1	1	1	1	1	1	1	1	1	1	1	4
SAL0001103	hp	hypothetical protein	1	1	1	1	1	1	1	1	1	1	1	3	1	1	1	1	1	1	1	1	1	1	1	1	1	1	1	1	1	1	1	1	1	1	1	1	1	1	1	1	2
SAL0001104	yhdN	General stress protein 69	1	1	1	1	1	3	1	1	1	1	1	1	1	1	1	1	1	1	1	1	1	1	1	1	1	1	1	1	1	1	1	1	1	1	1	1	1	1	1	1	2
SAL0001105	yheI_3	putative multidrug resistance ABC transporter ATP-binding/permease protein	1	1	3	1	1	1	1	5	2	1	1	1	1	1	3	2	2	2	2	1	1	2	2	2	2	2	1	3	1	3	1	3	1	1	2	1	1	2	2	1	4
SAL0001106	group_295	enterobactin exporter EntS	1	1	1	1	1	1	1	1	1	1	1	1	1	1	1	1	1	1	1	1	1	1	1	1	1	1	1	1	1	1	1	1	1	1	1	2	1	1	1	1	3
SAL0001107	rsmF	Ribosomal RNA small subunit methyltransferase F	1	1	1	1	1	1	1	1	1	1	1	4	1	1	1	1	1	1	1	1	1	1	1	1	1	1	1	1	1	1	1	1	1	1	1	3	1	1	1	1	2
SAL0001108	spsB_1	Signal peptidase IB	1	1	1	1	1	1	1	1	1	1	1	1	1	1	1	1	1	1	1	1	1	1	1	1	1	1	1	1	1	1	1	1	1	1	1	3	1	1	1	1	2
SAL0001109	cls	Cardiolipin synthase	1	1	1	1	1	1	1	3	1	1	1	1	1	1	1	1	1	1	1	1	1	1	1	1	1	1	1	1	1	1	1	1	1	1	1	1	1	1	1	1	2
SAL0001110	yafP	putative N-acetyltransferase YafP	1	1	1	1	1	1	1	1	1	1	1	3	1	1	1	1	1	1	1	1	1	1	1	1	1	1	1	1	1	1	1	1	1	1	1	1	1	1	1	1	2
SAL0001111	frr	Ribosome-recycling factor	1	1	1	1	1	1	1	1	1	1	1	1	1	1	1	1	1	1	1	1	1	1	1	1	1	1	1	1	1	1	1	1	1	1	2	2	1	1	1	1	1
SAL0001112	yheH	putative multidrug resistance ABC transporter ATP-binding/permease protein	1	1	2	1	1	6	3	4	1	3	1	2	1	1	2	1	1	1	1	1	1	1	1	1	1	1	1	4	5	2	1	2	1	1	1	1	1	1	1	1	2
SAL0001113	group_322	putative multidrug resistance ABC transporter ATP-binding/permease protein	1	1	2	1	1	1	1	2	1	1	1	3	1	1	2	1	1	1	1	1	1	1	1	1	1	1	1	2	1	2	1	2	1	1	1	1	1	1	1	1	1
SAL0001114	trmD	tRNA (guanine-N(1)-)-methyltransferase	1	1	1	1	1	1	1	1	1	1	1	1	1	1	1	1	1	1	1	1	1	1	1	1	1	1	1	1	1	1	1	1	1	1	4	2	1	1	1	1	3
SAL0001115	hp	hypothetical protein	1	1	1	1	1	1	1	2	1	1	1	1	1	1	1	1	1	1	1	1	1	1	1	1	1	1	1	1	1	1	1	1	1	1	1	1	1	1	1	1	3
SAL0001116	hp	hypothetical protein	1	1	1	1	1	1	1	1	1	1	1	1	1	1	1	1	1	1	1	1	1	1	1	1	1	1	1	1	1	1	1	1	1	1	1	1	1	1	1	1	2
SAL0001117	mfd	Transcription-repair-coupling factor	1	1	2	3	1	1	1	2	1	1	1	4	1	1	2	1	1	1	1	1	1	1	1	1	1	1	3	2	1	2	1	2	1	1	1	1	1	1	1	1	1
SAL0001118	dexB	Glucan 1,6-alpha-glucosidase	1	1	1	1	1	4	1	1	1	1	1	3	1	1	1	1	1	1	1	1	1	1	1	1	1	1	1	1	1	1	1	1	1	1	1	1	1	1	1	1	2
SAL0001119	hp	hypothetical protein	1	1	1	1	1	1	1	1	1	1	1	2	1	1	1	1	1	1	1	1	1	1	1	1	1	1	1	1	1	1	1	1	1	1	1	1	1	1	1	1	1
SAL0001120	bglA_2	6-phospho-beta-glucosidase BglA	1	1	1	1	1	1	1	1	1	1	1	2	1	1	1	1	1	1	1	1	1	1	1	1	1	1	1	1	1	1	1	1	1	1	1	1	1	1	1	1	3
SAL0001121	mgtA	Magnesium-transporting ATPase, P-type 1	1	1	1	1	1	4	1	5	1	1	1	1	1	1	2	1	1	1	1	1	1	1	1	1	1	1	1	1	6	1	1	1	1	1	1	1	1	1	1	1	3
SAL0001122	dppE_3	Dipeptide-binding protein DppE precursor	1	1	2	1	1	1	1	4	1	1	1	1	1	1	2	1	1	1	1	1	1	1	1	1	1	1	1	5	1	2	1	2	1	1	1	1	1	1	1	1	3
SAL0001123	lacZ	Beta-galactosidase LacZ	1	1	1	1	1	1	1	3	1	1	1	2	1	1	1	1	1	1	1	1	1	1	1	1	1	1	1	1	1	1	1	1	1	1	1	1	1	1	1	1	4
SAL0001124	ugpC	sn-glycerol-3-phosphate import ATP-binding protein UgpC	1	1	1	1	1	1	2	1	1	2	1	1	1	1	1	1	1	1	1	1	1	1	1	1	1	1	1	1	1	1	1	1	1	1	1	1	1	1	1	1	1
SAL0001125	yvgN	Glyoxal reductase	1	1	1	1	1	1	1	2	1	1	1	2	1	1	1	1	1	1	1	1	1	1	1	1	1	1	1	1	1	1	1	1	1	1	1	1	1	1	1	1	3
SAL0001126	hp	hypothetical protein	1	1	1	1	1	1	1	1	1	1	1	1	1	1	1	1	1	1	1	1	1	1	1	1	1	1	1	1	1	1	1	1	1	1	1	1	1	1	1	1	2
SAL0001127	yecD	Isochorismatase family protein YecD	1	1	1	1	1	1	1	3	1	1	1	4	1	1	1	1	1	1	1	1	1	1	1	1	1	1	1	1	1	1	1	1	1	1	1	1	1	1	1	1	2
SAL0001128	pgaC_2	Poly-beta-1,6-N-acetyl-D-glucosamine synthase	1	1	1	2	1	1	1	2	1	1	1	3	1	1	1	1	1	1	1	1	1	1	1	1	1	1	2	1	1	1	1	1	1	1	1	1	1	1	1	1	4
SAL0001129	tfdR	HTH-type transcriptional regulator TdfR	1	1	1	2	1	1	1	1	1	1	1	4	1	1	1	1	1	1	1	1	1	1	1	1	1	1	2	1	1	1	1	1	1	1	1	1	1	1	1	1	3
SAL0001130	appA_3	Oligopeptide-binding protein AppA precursor	1	1	1	1	1	1	1	1	1	1	1	1	1	1	1	1	1	1	1	1	1	1	1	1	1	1	1	1	1	1	1	1	1	1	1	1	1	1	1	1	2
SAL0001131	sdhA	L-serine dehydratase, alpha chain	1	1	1	1	1	1	1	1	1	1	1	1	1	1	3	1	1	1	1	1	1	1	1	1	1	1	1	1	4	1	1	1	1	1	1	1	1	1	1	1	2
SAL0001132	carB_2	Carbamoyl-phosphate synthase large chain	1	1	2	1	1	1	1	1	1	1	1	5	1	1	3	1	1	1	1	1	1	1	1	1	1	1	1	1	1	1	1	1	1	1	1	1	1	1	1	1	4
SAL0001133	glcU_2	Glucose uptake protein GlcU	1	1	3	1	1	1	1	4	1	1	1	1	1	1	2	1	1	1	1	1	1	1	1	1	1	1	1	1	1	2	1	2	1	1	1	1	1	1	1	1	5
SAL0001134	acm	Lysozyme M1 precursor	1	1	3	4	1	2	2	6	1	2	1	2	1	1	2	1	1	1	1	1	1	1	1	1	1	1	2	5	2	2	1	2	1	1	1	1	1	1	1	1	2
SAL0001135	agrA_1	Accessory gene regulator protein A	1	1	1	1	1	1	1	4	1	1	1	5	1	1	2	1	1	1	1	1	1	1	1	1	1	1	1	1	1	2	1	2	1	1	1	1	1	1	1	1	3
SAL0001136	yheI_5	putative multidrug resistance ABC transporter ATP-binding/permease protein	1	1	1	1	1	1	1	1	1	1	1	1	1	1	2	1	1	1	1	1	1	1	1	1	1	1	1	1	1	2	1	2	1	1	1	1	1	1	1	1	3
SAL0001137	glpQ1_2	putative glycerophosphoryl diester phosphodiesterase 1	1	1	1	1	1	1	1	1	1	1	1	1	1	1	1	1	1	1	1	1	1	1	1	1	1	1	1	1	1	1	1	1	1	1	1	1	1	1	1	1	2
SAL0001138	group_403	diadenosine tetraphosphatase	1	1	1	1	1	1	1	1	1	1	1	1	1	1	2	1	1	1	1	1	1	1	1	1	1	1	1	1	1	2	1	2	1	1	1	1	1	1	1	1	1
SAL0001139	hp	hypothetical protein	1	1	1	1	1	1	1	2	1	1	1	1	1	1	1	1	1	1	1	1	1	1	1	1	1	1	1	1	1	1	1	1	1	1	1	1	1	1	1	1	3
SAL0001140	cysL	HTH-type transcriptional regulator CysL	1	1	1	1	1	1	1	4	1	1	1	3	1	1	1	1	1	1	1	1	1	1	1	1	1	1	1	1	1	1	1	1	1	1	1	1	1	1	1	1	2
SAL0001141	prtP	PII-type proteinase precursor	1	1	5	2	1	4	3	1	1	3	1	1	1	1	1	1	1	1	1	1	1	1	1	1	1	1	2	1	1	1	1	1	1	1	1	1	1	1	1	1	6
SAL0001142	arlS_1	Signal transduction histidine-protein kinase ArlS	1	1	1	1	1	1	1	2	1	1	1	4	1	1	1	1	1	1	1	1	1	1	1	1	1	1	1	2	5	1	1	1	1	1	1	1	1	1	1	1	3
SAL0001143	bglH_4	Aryl-phospho-beta-D-glucosidase BglH	1	1	1	1	1	1	1	2	1	1	1	1	1	1	1	1	1	1	1	1	1	1	1	1	1	1	1	1	1	1	1	1	1	1	1	1	1	1	1	1	3
SAL0001144	sacX	Negative regulator of SacY activity	3	3	3	3	3	3	3	3	3	3	3	3	4	3	3	3	3	3	3	3	3	3	3	3	3	3	3	3	3	3	3	3	3	3	3	1	3	3	3	3	2

SAL0001145	pbpX_5	Putative penicillin-binding protein PbpX	1	1	1	1	1	1	1	2	1	1	1	3	1	1	1	1	1	1	1	1	1	1	1	1	1	1	1	1	1	1	1	1	1	1	1	1	1	1	1	1	4
SAL0001146	hp	hypothetical protein	1	1	1	1	1	1	1	1	1	1	1	1	1	1	1	1	1	1	1	1	1	1	1	1	1	1	1	1	1	1	1	1	1	1	1	1	1	1	1	1	2
SAL0001147	malF	Maltose transport system permease protein MalF	1	1	1	1	1	1	1	1	1	1	1	1	1	1	1	1	1	1	1	1	1	1	1	1	1	1	1	1	1	1	1	1	1	1	1	1	1	1	1	1	2
SAL0001148	hp	hypothetical protein	1	1	1	1	1	1	1	1	1	1	1	1	1	1	1	1	1	1	1	1	1	1	1	1	1	1	1	1	1	1	1	1	1	1	1	1	1	1	1	1	2
SAL0001149	srlR	Glucitol operon repressor	1	1	2	4	1	1	3	5	1	3	1	1	1	1	2	1	1	1	1	1	1	1	1	1	1	1	4	5	1	2	1	2	1	1	1	1	1	1	1	1	1
**SAL0001150**	**hp**	**hypothetical protein**	1	1	5	1	1	4	3	11	1	3	1	4	1	1	2	1	1	1	1	1	1	1	7	1	1	1	1	8	10	2	1	2	1	1	1	1	1	1	1	9	6
SAL0001151	hflX	GTPase HflX	1	1	2	1	1	1	1	4	1	1	1	1	1	1	2	1	1	1	1	1	1	1	1	1	1	1	1	5	1	2	1	2	1	1	1	1	1	1	1	1	3
SAL0001152	helD_2	Helicase IV	1	1	2	2	1	2	2	3	1	2	1	4	1	1	2	1	1	1	1	1	1	1	1	1	1	1	2	3	2	2	1	2	1	1	1	1	1	1	1	1	5
SAL0001153	fliY	Cystine-binding periplasmic protein precursor	1	1	2	1	1	1	1	3	1	1	1	1	1	1	2	1	1	1	1	1	1	1	1	1	1	1	1	3	1	2	1	2	1	1	1	1	1	1	1	1	4
SAL0001154	ndvA_2	Beta-(1-->2)glucan export ATP-binding/permease protein NdvA	1	1	2	3	1	1	1	2	1	1	1	1	1	1	2	1	1	1	1	1	1	1	1	1	1	1	3	2	1	2	1	2	1	1	1	5	1	1	1	1	4
SAL0001155	hp	hypothetical protein	1	1	5	1	1	1	1	6	1	1	1	2	3	1	1	1	1	1	1	1	1	1	1	1	1	1	1	1	7	1	1	1	1	1	1	1	1	1	1	1	4
SAL0001156	xpsE	Type II secretion system protein E	1	1	1	2	1	1	1	2	1	1	1	1	5	1	1	1	1	1	1	1	1	1	1	1	1	1	2	1	3	1	1	1	1	1	1	1	1	1	1	1	4
SAL0001157	group_499	Nucleoid-associated protein	1	1	2	1	1	1	1	1	1	1	1	1	1	1	1	1	1	1	1	1	1	1	1	1	1	1	1	1	1	1	1	1	1	1	1	1	1	1	1	1	1
SAL0001158	hp	hypothetical protein	1	1	1	1	1	1	1	2	1	1	1	1	1	1	1	1	1	1	1	1	1	1	1	1	1	1	1	1	1	1	1	1	1	1	1	1	1	1	1	1	1
SAL0001159	rsmI	Ribosomal RNA small subunit methyltransferase I	1	1	1	2	1	1	1	2	1	1	1	1	1	1	1	1	1	1	1	1	1	1	1	1	1	1	2	1	1	1	1	1	1	1	1	1	1	1	1	1	3
**SAL0001160**	**rr**	**Response regulator**	1	1	7	3	1	8	1	2	1	1	1	4	1	1	2	1	1	1	1	1	1	1	1	1	1	1	3	2	5	2	1	2	1	1	1	1	1	1	6	1	1
SAL0001161	glpQ1_1	putative glycerophosphoryl diester phosphodiesterase 1	1	1	1	2	1	3	1	1	1	1	1	1	1	1	1	1	1	1	1	1	1	1	1	1	1	1	2	1	1	1	1	1	1	1	1	1	1	1	1	1	4
SAL0001162	hp	hypothetical protein	1	1	1	1	1	1	1	1	1	1	1	3	1	1	1	1	1	1	1	1	1	1	1	1	1	1	1	1	1	1	1	1	1	1	1	1	1	1	1	1	2
SAL0001163	ybiR	Inner membrane protein YbiR	1	1	3	2	1	1	4	6	1	4	1	2	1	1	3	1	1	1	1	1	1	1	1	1	1	1	2	3	2	3	1	3	1	1	1	1	1	1	2	1	5
SAL0001164	hp	hypothetical protein	1	1	1	1	1	1	1	3	1	1	1	1	1	1	1	1	1	1	1	1	1	1	1	1	1	1	1	1	1	1	1	1	1	1	1	1	1	1	1	1	2
SAL0001165	group_542	Helix-turn-helix domain protein	1	1	1	1	1	1	2	3	1	2	1	1	1	1	1	1	1	1	1	1	1	1	1	1	1	1	1	1	1	1	1	1	1	1	1	1	1	1	1	1	4
SAL0001166	group_546	Putative glutamine amidotransferase	1	1	1	1	1	1	1	1	1	1	1	3	1	1	1	1	1	1	1	1	1	1	1	1	1	1	1	1	1	1	1	1	1	1	1	1	1	1	1	1	2
SAL0001167	asnA	Aspartate--ammonia ligase	1	1	1	4	1	1	1	3	1	1	1	1	1	1	1	1	1	1	1	1	1	1	1	1	1	1	1	1	2	1	1	1	1	1	1	1	1	1	1	1	1
SAL0001168	malL	Oligo-1,6-glucosidase	1	1	3	1	1	1	1	1	1	1	1	1	1	1	2	1	1	1	1	1	1	1	1	1	1	1	1	1	1	2	1	2	1	1	1	1	1	1	1	1	4
SAL0001169	ltaS1	Lipoteichoic acid synthase 1	1	1	1	1	1	5	1	3	1	1	1	4	1	1	1	1	2	1	1	1	1	1	1	1	1	1	1	1	1	1	1	1	1	1	1	1	1	1	1	6	7
SAL0001170	hp	hypothetical protein	2	2	2	3	2	2	2	2	2	2	2	2	2	2	2	2	2	2	2	2	2	2	2	2	2	2	3	2	2	2	2	2	2	2	2	2	2	2	2	2	4
SAL0001171	group_580	ribonuclease BN/unknown domain fusion protein	1	1	1	1	1	1	1	2	1	1	1	1	1	1	1	1	1	1	1	1	1	1	1	1	1	1	1	2	1	1	1	1	1	1	1	1	1	1	1	1	3
SAL0001172	gtaB_1	UTP--glucose-1-phosphate uridylyltransferase	1	1	1	1	1	1	1	2	1	1	1	1	1	1	1	1	1	1	1	1	1	1	1	1	1	1	1	1	1	1	1	1	1	1	1	1	1	1	1	1	1
SAL0001173	thlA	Acetyl-CoA acetyltransferase	1	1	1	1	1	1	1	1	1	1	1	1	1	1	1	1	1	1	1	1	1	1	2	1	1	1	3	1	1	1	1	1	1	1	1	1	1	1	1	1	1
SAL0001174	recX	Regulatory protein RecX	1	1	2	1	1	1	1	1	1	1	1	1	1	1	1	1	1	1	1	1	1	1	1	1	1	1	1	1	1	1	1	1	1	1	1	1	1	1	1	1	3
SAL0001175	prfC	Peptide chain release factor 3	1	1	1	1	1	1	1	1	1	1	1	1	1	1	1	1	1	1	1	1	1	1	1	1	1	1	3	1	1	1	1	1	1	1	1	1	1	1	1	1	2
SAL0001176	ptsI	Phosphoenolpyruvate-protein phosphotransferase	1	1	1	1	1	1	1	2	1	1	1	5	1	1	1	1	1	1	1	1	1	1	3	1	1	1	1	1	1	1	1	1	1	1	1	1	1	1	1	1	4
SAL0001177	hp	hypothetical protein	1	1	1	1	1	1	1	1	1	1	3	1	2	1	1	1	1	1	1	1	1	1	1	1	1	1	1	1	1	1	1	1	1	1	1	1	1	1	1	1	4
SAL0001178	group_587	SNARE associated Golgi protein	1	1	1	1	1	1	1	1	1	1	1	3	1	1	1	1	1	1	1	1	1	1	1	1	1	1	1	1	1	1	1	1	1	1	1	1	1	1	1	1	2
SAL0001179	hp	hypothetical protein	1	1	1	2	1	1	1	2	1	1	1	3	1	1	1	1	1	1	1	1	1	1	1	1	1	1	1	1	1	1	1	1	1	1	1	1	1	1	1	1	4
SAL0001180	hp	hypothetical protein	1	1	1	1	1	1	1	2	1	1	1	1	1	1	1	1	1	1	1	1	1	1	1	1	1	1	1	1	1	1	1	1	1	1	1	1	1	1	1	1	3
SAL0001181	group_590	Putative gluconeogenesis factor	1	1	1	3	1	1	1	4	1	1	1	1	1	1	2	1	1	1	1	1	1	1	1	1	1	1	1	1	1	2	1	2	1	1	1	1	1	1	1	1	5
SAL0001182	hp	hypothetical protein	1	1	1	1	1	1	1	1	1	1	1	2	1	1	1	1	1	1	1	1	1	1	1	1	1	1	2	1	1	1	1	1	1	1	1	1	1	1	1	1	1
SAL0001183	yfkJ	Low molecular weight protein-tyrosine-phosphatase YfkJ	1	1	1	1	1	1	1	1	1	1	1	1	1	1	1	1	1	1	1	1	1	1	1	1	1	1	1	1	1	1	1	1	1	1	1	1	1	1	1	1	1
SAL0001184	licT_1	Transcription antiterminator LicT	1	1	3	1	1	1	1	5	1	1	1	1	1	1	2	1	1	1	1	1	1	1	1	1	1	1	1	6	1	2	1	2	1	1	1	1	1	1	1	1	4
SAL0001185	group_594	VanZ like family protein	1	1	1	1	1	1	1	1	1	1	1	2	1	1	1	1	1	1	1	1	1	1	1	1	1	1	1	1	1	1	1	1	1	1	1	1	1	1	1	1	3
SAL0001186	pulG	Type II secretion system protein G precursor	1	1	1	1	1	1	1	1	1	1	1	1	1	1	1	1	1	1	1	1	1	1	1	1	1	1	1	1	1	1	1	1	1	1	1	1	1	1	1	1	2
SAL0001187	patB	Cystathionine beta-lyase PatB	1	1	1	1	1	1	1	1	1	1	1	2	1	1	1	1	1	1	1	1	1	1	1	1	1	1	1	1	1	1	1	1	1	1	1	1	1	1	1	1	3
SAL0001188	baeS	Signal transduction histidine-protein kinase BaeS	1	1	1	1	1	1	1	3	1	1	1	1	1	1	1	1	1	1	1	1	1	1	1	1	1	1	1	1	1	1	1	1	1	1	1	1	1	1	1	1	2
SAL0001189	atpE	ATP synthase subunit c	1	1	1	1	1	1	4	1	1	3	1	1	1	1	1	1	1	1	1	1	1	1	1	1	1	1	1	1	2	1	1	1	1	1	1	1	1	1	1	1	1
SAL0001190	hp	hypothetical protein	1	1	1	1	1	1	1	1	1	1	1	1	1	1	1	1	1	1	1	1	1	1	1	1	1	1	1	1	1	1	1	1	1	1	1	1	1	1	1	1	2
SAL0001191	group_602	YGGT family protein	1	1	2	1	1	2	2	2	1	2	1	2	1	1	2	1	1	1	1	1	1	1	1	1	1	1	1	2	2	2	1	2	1	1	1	1	1	1	1	1	2
SAL0001192	group_603	tetratricopeptide repeat protein	1	1	1	2	1	1	1	2	1	1	1	1	1	1	1	1	1	1	1	1	1	1	1	1	1	1	2	1	1	1	1	1	1	1	1	1	1	1	1	1	3
SAL0001193	recD2	ATP-dependent RecD-like DNA helicase	1	1	1	1	1	1	1	1	1	1	1	1	1	1	3	1	1	1	1	1	1	1	1	1	1	1	1	1	2	1	1	1	1	1	1	1	1	1	1	1	4
SAL0001194	group_605	Nuclease-related domain protein	1	1	6	1	1	1	1	1	1	1	1	1	1	1	4	1	1	1	1	1	1	1	1	1	1	1	1	1	5	4	1	4	1	1	1	1	1	1	1	1	1
SAL0001195	rnjA	Ribonuclease J 1	1	1	1	1	1	1	1	1	1	1	1	1	2	1	1	1	1	1	1	1	1	1	1	1	1	1	1	1	1	1	1	1	1	1	1	1	1	1	1	1	1
SAL0001196	ftsW	Lipid II flippase FtsW	2	2	4	2	2	3	2	2	2	2	2	2	2	2	2	2	2	2	2	2	2	2	2	2	2	2	2	2	2	2	2	2	2	2	2	2	2	2	2	2	5
SAL0001197	rpsO	30S ribosomal protein S15	1	1	1	1	1	1	1	1	1	1	1	1	1	1	1	1	1	1	1	1	1	1	1	1	1	1	1	1	1	1	1	1	1	1	1	2	1	1	1	1	1
SAL0001198	dapH	2,3,4,5-tetrahydropyridine-2,6-dicarboxylate N-acetyltransferase	1	1	1	1	1	1	1	3	1	1	1	1	1	1	1	1	1	1	1	1	1	1	1	1	1	1	1	1	1	1	1	1	1	1	1	1	1	1	1	1	2
SAL0001199	gmuC_3	Oligo-beta-mannoside permease IIC component	1	1	2	1	1	1	1	1	1	1	1	5	1	1	2	1	1	1	1	1	1	1	1	1	1	1	1	1	3	2	1	2	1	1	1	1	1	1	1	4	6
SAL0001200	bglK_1	Beta-glucoside kinase	1	1	1	1	1	1	1	1	1	1	1	2	1	1	1	1	1	1	1	1	1	1	1	1	1	1	1	1	1	1	1	1	1	1	1	1	1	1	1	1	3
SAL0001201	hp	hypothetical protein	1	1	1	1	1	1	1	1	1	1	1	2	1	1	1	1	1	1	1	1	1	1	1	1	1	1	1	1	1	1	1	1	1	1	1	1	1	1	1	1	1
SAL0001202	thyA	Thymidylate synthase	1	1	1	2	1	1	1	1	1	1	1	1	1	1	1	1	1	1	1	1	1	1	1	1	1	1	1	1	1	1	1	1	1	1	1	1	1	1	1	1	1
SAL0001203	metN2	Methionine import ATP-binding protein MetN 2	1	1	1	2	1	1	1	1	1	1	1	1	1	1	1	1	1	1	1	1	1	1	1	1	1	1	2	1	1	1	1	1	1	1	1	1	1	1	1	1	1
SAL0001204	fumC	Fumarate hydratase class II	1	1	1	1	1	4	3	1	1	3	2	1	1	1	1	1	1	1	1	2	2	1	1	1	1	1	1	1	1	1	2	1	1	1	1	1	1	1	1	1	5
SAL0001205	hp	hypothetical protein	1	1	1	1	1	1	1	1	1	1	1	1	1	1	1	1	1	1	1	1	1	1	1	1	1	1	1	1	1	1	1	1	1	1	1	1	1	1	1	1	2
SAL0001206	ecfT_1	Energy-coupling factor transporter transmembrane protein EcfT	1	1	1	2	1	1	1	2	1	1	1	1	1	1	1	1	1	1	1	1	1	1	1	1	1	1	2	1	1	1	1	1	1	1	1	1	1	1	1	1	3
SAL0001207	ykoD_1	Putative HMP/thiamine import ATP-binding protein YkoD	1	1	1	1	1	1	1	2	1	1	1	1	1	1	1	1	1	1	1	1	1	1	1	1	1	1	1	3	1	1	1	1	1	1	1	1	1	1	1	1	4
SAL0001208	yjjP_1	Inner membrane protein YjjP	1	1	1	1	1	1	1	1	1	1	1	1	2	1	1	1	1	1	1	1	1	1	1	1	1	1	1	1	1	1	1	1	1	1	1	4	1	1	1	1	3

SAL0001209	yhdG_1	putative amino acid permease YhdG	1	1	1	1	1	1	1	1	1	1	1	1	1	1	1	1	1	1	1	1	1	1	1	1	1	1	1	1	1	1	1	1	1	1	1	2	1	1	1	1	3
SAL0001210	hp	hypothetical protein	1	1	1	1	1	1	1	1	1	1	1	3	1	1	1	1	1	1	1	1	1	1	1	1	1	1	1	1	1	1	1	1	1	1	1	2	1	1	1	1	4
SAL0001211	pfkA	6-phosphofructokinase	1	1	1	1	1	1	1	1	1	1	1	1	1	1	1	1	1	1	1	1	1	1	2	1	1	1	1	1	1	1	1	1	1	1	1	1	1	1	1	1	1
SAL0001212	ktrA	Ktr system potassium uptake protein A	1	1	1	1	1	1	1	2	1	1	1	1	1	1	1	1	1	1	1	1	1	1	1	1	1	1	1	1	1	1	1	1	1	1	1	1	1	1	1	1	3
SAL0001213	group_626	flavodoxin	1	1	2	1	1	1	3	1	1	3	1	1	1	1	2	1	1	1	1	1	1	1	1	1	1	1	1	1	1	2	1	2	1	1	1	1	1	1	1	1	4
SAL0001214	yvbK	putative N-acetyltransferase YvbK	1	1	1	1	1	1	1	1	1	1	1	1	1	1	1	1	1	1	1	1	1	1	1	1	1	1	1	1	1	1	1	1	1	1	1	1	1	1	1	1	2
SAL0001215	group_628	MucBP domain protein	1	1	1	1	1	1	1	1	1	1	1	1	1	1	1	1	1	1	1	1	1	1	1	1	1	1	1	1	1	1	1	1	1	1	1	1	1	1	1	1	2
SAL0001216	hp	hypothetical protein	1	1	1	1	1	1	1	1	1	1	1	1	1	1	1	1	1	1	1	1	1	1	1	1	1	1	1	1	1	1	1	1	1	1	1	1	1	1	1	1	2
SAL0001217	cynR_1	HTH-type transcriptional regulator CynR	1	1	4	2	1	1	1	1	1	1	1	1	1	1	1	1	1	1	1	1	1	1	1	1	1	1	2	1	5	1	1	1	1	1	1	1	1	1	1	1	3
SAL0001218	group_631	putative oxidoreductase/MSMEI_2347	1	1	1	1	1	1	1	1	1	1	1	3	1	1	1	1	1	1	1	1	1	1	1	1	1	1	1	1	4	1	1	1	1	1	1	1	1	1	1	1	2
SAL0001219	hp	hypothetical protein	1	1	1	3	1	1	1	1	1	1	1	1	1	1	2	1	1	1	1	1	1	1	1	1	1	1	3	1	1	2	1	2	1	1	1	1	1	1	1	1	4
SAL0001220	group_633	Acetyltransferase (GNAT) family protein	1	1	3	1	1	1	1	1	1	1	1	1	1	1	1	1	1	1	1	1	1	1	1	1	1	1	1	1	1	1	1	1	1	1	1	1	1	1	1	1	2
SAL0001221	hp	hypothetical protein	1	1	1	1	1	1	1	1	1	1	1	2	1	1	1	1	1	1	1	1	1	1	1	1	1	1	1	1	1	1	1	1	1	1	1	1	1	1	1	1	3
SAL0001222	group_635	iron-responsive transcriptional regulator	1	1	1	2	1	1	1	2	1	1	1	2	1	1	1	1	1	1	1	1	1	1	1	1	1	1	1	1	1	1	1	1	1	1	1	1	1	1	1	1	3
SAL0001223	group_636	putative oxidoreductase/MSMEI_2347	1	1	1	1	1	1	1	1	1	1	1	1	1	1	1	1	1	1	1	1	1	1	1	1	1	1	1	1	1	1	1	1	1	1	1	1	1	1	1	1	2
SAL0001224	group_637	Bacterial Ig-like domain (group 3)	1	1	2	4	1	1	1	2	1	1	1	1	1	1	2	1	1	1	1	1	1	1	1	1	1	1	1	2	1	2	1	2	1	1	1	1	1	1	1	1	3
SAL0001225	hp	hypothetical protein	1	1	1	1	1	1	1	1	1	1	1	1	1	1	1	1	1	1	1	1	1	1	1	1	1	1	1	1	1	1	1	1	1	1	1	1	1	1	1	1	2
SAL0001226	group_639	ABC transporter glutamine-binding protein GlnH precursor	1	1	2	1	1	4	2	2	1	2	1	3	1	1	6	1	1	1	1	1	1	1	1	1	1	1	1	2	2	2	1	2	1	1	1	1	1	1	1	1	5
SAL0001227	hp	hypothetical protein	1	1	1	1	1	1	1	1	1	1	1	1	1	1	1	1	1	1	1	1	1	1	1	1	1	1	1	1	1	1	1	1	1	1	1	1	1	1	1	1	1
SAL0001228	hp	hypothetical protein	1	1	1	1	1	1	1	1	1	1	1	1	1	1	1	1	1	1	1	1	1	1	1	1	1	1	1	1	1	1	1	1	1	1	1	1	1	1	1	1	2
SAL0001229	ydaF_1	Putative ribosomal N-acetyltransferase YdaF	1	1	1	1	1	1	1	1	1	1	1	1	1	1	1	1	1	1	1	1	1	1	1	1	1	1	1	1	1	1	1	1	1	1	1	1	1	1	1	1	2
SAL0001230	group_643	AMP nucleosidase	1	1	1	1	1	1	1	1	1	1	1	1	1	1	1	1	1	1	1	1	1	1	1	1	1	1	1	1	3	1	1	1	1	1	1	1	1	1	1	1	2
SAL0001231	group_644	AMP nucleosidase	1	1	1	1	1	2	1	1	1	1	1	1	1	1	1	1	1	1	1	1	1	1	1	1	1	1	1	1	1	1	1	1	1	1	1	1	1	1	1	1	1
SAL0001232	hp	hypothetical protein	1	1	1	1	1	1	1	1	1	1	1	1	1	1	1	1	1	1	1	1	1	1	1	1	1	1	1	1	1	1	1	1	1	1	1	1	1	1	1	1	2
SAL0001233	hp	hypothetical protein	1	1	1	1	1	1	1	1	1	1	1	1	1	1	1	1	1	1	1	1	1	1	1	1	1	1	1	1	1	1	1	1	1	1	1	1	1	1	1	1	2
SAL0001234	ppc	Phosphoenolpyruvate carboxylase	1	1	2	2	1	4	2	2	1	2	1	2	1	1	2	1	1	1	1	1	1	1	1	1	1	1	2	2	2	3	1	3	1	1	1	1	1	1	1	1	5
SAL0001235	gdh	Glucose 1-dehydrogenase	1	1	1	1	1	1	1	1	1	1	1	1	1	1	2	1	1	1	1	1	1	1	1	1	1	1	1	1	2	1	1	1	1	1	1	1	1	1	1	1	3
SAL0001236	hp	hypothetical protein	1	1	1	1	1	1	1	1	1	1	1	1	1	1	1	1	1	1	1	1	1	1	1	1	1	1	1	1	1	1	1	1	1	1	1	1	1	1	1	1	2
SAL0001237	adh	NADP-dependent isopropanol dehydrogenase	1	1	1	1	1	1	1	1	1	1	1	1	1	1	1	1	1	1	1	1	1	1	1	1	1	1	1	1	1	1	1	1	1	1	1	1	1	1	1	1	2
SAL0001238	azoR2	FMN-dependent NADH-azoreductase 2	1	1	1	1	1	1	1	1	1	1	1	1	1	1	1	1	1	1	1	1	1	1	1	1	1	1	1	1	1	1	1	1	1	1	1	1	1	1	1	1	2
SAL0001239	xerS	Tyrosine recombinase XerS	1	1	1	1	1	1	1	1	1	1	1	2	1	1	1	1	1	1	1	1	1	1	1	1	1	1	1	1	1	1	1	1	1	1	1	3	1	1	1	1	1
SAL0001240	lspA	Lipoprotein signal peptidase	2	2	2	2	2	2	2	2	2	2	2	3	2	2	2	2	2	2	2	2	2	2	2	2	2	2	2	2	2	2	2	2	2	2	2	2	2	2	2	2	2
SAL0001241	gpsB	Cell cycle protein GpsB	1	1	1	1	1	1	1	1	1	1	1	1	1	1	1	1	1	1	1	1	1	1	1	1	1	1	1	1	1	1	1	1	1	1	1	1	1	1	1	2	3
SAL0001242	asnS	Asparagine--tRNA ligase	1	1	1	1	1	1	1	1	1	1	1	1	1	1	1	1	1	1	1	1	1	1	1	1	1	1	1	1	1	1	1	1	1	1	1	2	1	1	1	1	3
SAL0001243	hp	hypothetical protein	1	1	1	1	1	1	1	1	1	1	1	2	1	1	1	1	1	1	1	1	1	1	1	1	1	1	1	1	1	1	1	1	1	1	1	1	1	1	1	1	1
SAL0001244	ybbH_1	putative HTH-type transcriptional regulator YbbH	1	1	1	1	1	1	1	3	1	1	1	1	1	1	1	1	1	1	1	1	1	1	1	1	1	1	1	1	1	1	1	1	1	1	1	1	1	1	1	1	2
SAL0001245	hp	hypothetical protein	1	1	1	1	1	1	1	2	1	1	1	1	1	1	1	1	1	1	1	1	1	1	1	1	1	1	1	1	1	1	1	1	1	1	1	1	1	1	1	1	1
SAL0001246	albC	Putative ABC transporter ATP-binding protein AlbC	1	1	3	1	1	1	1	1	1	1	1	1	1	1	1	1	1	1	1	1	1	1	1	1	1	1	1	1	1	1	1	1	1	1	1	1	1	1	1	1	2
SAL0001247	ytrA	HTH-type transcriptional repressor YtrA	1	1	1	1	1	1	1	1	1	1	1	1	1	1	1	1	1	1	1	1	1	1	1	1	1	1	1	1	1	1	1	1	2	1	1	1	1	1	1	1	3
SAL0001248	pepT_1	Peptidase T	1	1	1	1	1	1	1	1	1	1	1	1	1	1	1	1	1	1	1	1	1	1	1	1	1	1	1	1	1	1	1	1	1	1	1	1	1	1	1	1	2
SAL0001249	group_663	Peptidase family M23	1	1	2	1	1	1	1	3	1	1	1	1	1	1	2	1	1	1	1	1	1	1	1	1	1	1	1	2	1	2	1	2	1	1	1	1	1	1	1	1	4
SAL0001250	ybeZ	PhoH-like protein	1	1	2	1	1	6	1	3	1	1	1	7	1	1	2	1	1	1	1	1	1	1	1	1	1	1	1	3	5	2	1	2	1	1	1	1	1	1	1	1	4
SAL0001251	group_665	Peptidase family M23	1	1	1	1	1	1	1	1	1	1	1	1	1	1	1	1	1	1	1	2	1	1	1	1	1	1	1	1	1	1	1	1	1	1	1	1	1	1	1	1	1
SAL0001252	thrB	Homoserine kinase	1	1	1	1	1	1	3	1	1	3	1	1	1	1	2	1	1	1	1	1	1	1	1	1	1	1	1	1	1	2	1	2	1	1	1	1	1	1	1	1	4
SAL0001253	maa_1	Maltose O-acetyltransferase	1	1	2	1	1	1	1	1	1	1	1	1	1	1	1	1	1	1	1	1	1	1	1	1	1	1	1	1	1	1	1	1	1	1	1	1	1	1	1	1	3
SAL0001254	merA	Mercuric reductase	1	1	4	1	1	1	1	1	1	1	1	2	1	1	1	1	1	1	1	1	1	1	1	1	1	1	1	1	1	1	1	1	1	1	1	1	1	1	1	1	3
SAL0001255	hp	hypothetical protein	1	1	1	1	1	1	1	1	1	1	1	1	1	1	1	1	1	1	1	1	1	1	1	1	1	1	1	2	1	1	1	1	1	1	1	1	1	1	1	1	1
SAL0001256	hp	hypothetical protein	1	1	1	1	1	1	1	1	1	1	1	1	1	1	1	1	1	1	1	1	1	1	1	1	1	1	1	1	1	1	1	1	1	1	1	1	1	1	1	1	2
SAL0001257	dnaJ	Chaperone protein DnaJ	1	1	1	1	1	1	1	2	1	1	1	1	1	1	1	1	1	1	1	1	1	1	3	1	1	1	1	1	1	1	1	1	1	1	1	1	1	1	1	1	1
SAL0001258	yheS_1	putative ABC transporter ATP-binding protein YheS	1	1	2	1	1	6	1	3	1	1	1	1	1	1	2	1	1	1	1	1	1	1	1	1	1	1	1	3	4	2	1	2	1	1	1	1	1	1	1	1	5
SAL0001259	group_673	GDSL-like Lipase/Acylhydrolase	1	1	1	1	1	1	1	1	1	1	1	1	1	1	1	1	1	1	1	1	1	1	1	1	1	1	1	1	2	1	1	1	1	1	1	1	1	1	1	1	3
SAL0001260	hp	hypothetical protein	1	1	1	1	1	1	1	1	1	1	1	1	1	1	1	1	1	1	1	1	1	1	1	1	1	1	1	1	1	1	1	1	1	1	1	2	1	1	1	1	1
SAL0001261	group_675	Ion channel	1	1	1	1	1	1	1	1	1	1	1	1	1	1	1	1	1	1	1	1	1	1	1	1	1	1	1	1	1	1	1	1	1	1	1	3	1	1	1	1	2
SAL0001262	hp	hypothetical protein	1	1	2	1	1	1	1	1	1	1	1	1	1	1	2	1	1	1	1	1	1	1	1	1	1	1	1	1	1	2	1	2	1	1	1	3	1	1	1	1	1
SAL0001263	hp	hypothetical protein	1	1	1	1	1	1	1	1	1	1	1	1	1	1	1	1	1	1	1	1	1	1	1	1	1	1	1	1	1	1	1	1	1	1	1	1	1	1	1	1	2
SAL0001264	sigH	RNA polymerase sigma-H factor	1	1	1	1	1	1	1	1	1	1	1	3	1	1	1	1	1	1	1	1	1	1	1	1	1	1	1	1	1	1	1	1	1	1	1	1	1	1	1	1	2
SAL0001265	ecfA2	Energy-coupling factor transporter ATP-binding protein EcfA2	1	1	1	1	1	1	1	1	1	1	1	1	1	1	1	1	1	1	1	1	1	1	1	1	1	1	1	1	1	1	1	1	1	1	1	1	1	1	1	1	2
SAL0001266	clpC	ATP-dependent Clp protease ATP-binding subunit ClpC	1	1	1	2	1	1	1	5	1	1	1	1	1	1	1	1	1	1	1	1	1	1	1	1	1	1	2	1	6	3	1	3	1	1	1	1	1	1	1	7	4
SAL0001267	msrC	Free methionine-R-sulfoxide reductase	1	1	1	1	1	1	1	1	1	1	1	1	1	1	1	1	1	1	1	1	1	1	1	1	1	1	1	1	1	1	1	1	1	1	1	1	1	1	1	1	2
SAL0001268	hp	hypothetical protein	1	1	1	1	1	4	1	1	1	1	1	3	1	1	1	1	1	1	1	1	1	1	1	1	1	1	1	1	2	1	1	1	1	1	1	1	1	1	1	1	1
SAL0001269	hp	hypothetical protein	1	1	1	1	1	1	1	1	1	1	1	1	1	1	1	1	1	1	1	1	1	1	1	1	1	1	1	1	1	1	1	1	1	1	1	1	1	1	1	1	2
SAL0001270	ogt	Methylated-DNA--protein-cysteine methyltransferase	1	1	1	1	1	1	1	2	1	1	1	1	1	1	1	1	1	1	1	1	1	1	1	1	1	1	1	2	1	1	1	1	1	1	1	1	1	1	1	1	3
SAL0001271	group_685	ABC-2 family transporter protein	1	1	1	1	1	1	1	1	1	1	1	1	1	1	1	1	1	1	1	1	1	1	1	1	1	1	1	3	1	1	1	1	1	1	1	1	1	1	1	1	2
SAL0001272	drrA	Daunorubicin/doxorubicin resistance ATP-binding protein DrrA	1	1	1	1	1	3	1	1	1	1	1	1	1	1	1	1	1	1	1	1	1	1	1	1	1	1	1	1	1	1	1	1	1	1	1	1	1	1	1	1	2

SAL0001273	hp	hypothetical protein	1	1	1	1	1	1	1	1	1	1	1	1	1	1	1	1	1	1	1	1	1	1	2	1	1	1	1	1	1	1	1	1	1	1	1	1	1	1	1	1	3
SAL0001274	rpoE	putative DNA-directed RNA polymerase subunit delta	1	1	1	1	1	1	1	1	1	1	1	1	1	1	1	1	1	1	1	1	1	1	1	1	1	1	1	1	1	1	1	1	1	1	1	1	1	1	1	1	2
SAL0001275	group_689	deoxyguanosinetriphosphate triphosphohydrolase-like protein	1	1	1	1	1	1	1	1	1	1	1	1	1	1	1	1	1	1	1	1	1	1	1	1	1	1	1	1	1	1	1	1	1	1	1	1	1	1	1	3	2
SAL0001276	licC_1	Lichenan permease IIC component	1	1	1	1	1	4	1	1	1	1	1	3	1	1	1	1	1	1	1	1	1	1	1	1	1	1	1	1	2	1	1	1	1	1	1	1	1	1	1	1	1
SAL0001277	rnmV	Ribonuclease M5	1	1	2	1	1	1	1	1	1	1	1	1	1	1	1	1	1	1	1	1	1	1	1	1	1	1	1	1	1	1	1	1	1	1	1	1	1	1	1	1	3
SAL0001278	guaB	Inosine-5'-monophosphate dehydrogenase	1	1	1	1	1	1	1	2	1	1	1	1	1	1	1	1	1	1	1	1	1	1	1	1	1	1	1	1	1	1	1	1	1	1	1	1	1	1	1	1	1
SAL0001279	group_693	Sulfatase	1	1	4	1	1	1	1	1	1	1	1	1	1	1	2	1	1	1	1	1	1	1	5	1	1	1	6	1	1	2	1	2	1	1	1	1	1	1	1	1	3
SAL0001280	lytR_2	Transcriptional regulator LytR	1	1	1	1	1	1	1	1	1	1	1	2	1	1	1	1	1	1	1	1	1	1	1	1	1	1	1	1	4	1	1	1	1	1	1	1	1	1	1	1	3
SAL0001281	hp	hypothetical protein	1	1	1	1	1	1	1	1	1	1	1	2	1	1	1	1	1	1	1	1	1	1	1	1	1	1	1	1	1	1	1	1	1	1	1	1	1	1	1	1	3
SAL0001282	dhaK	PTS-dependent dihydroxyacetone kinase, dihydroxyacetone-binding subunit [illegible]	1	1	1	1	1	5	1	2	1	1	1	1	1	1	1	1	1	1	1	1	1	1	1	1	1	1	1	1	4	1	1	1	1	1	1	1	1	1	1	1	3
SAL0001283	hp	hypothetical protein	1	1	1	1	1	1	1	1	1	1	1	1	1	1	1	1	1	1	1	1	1	1	1	1	1	1	1	1	1	1	1	1	1	1	1	1	1	1	1	1	2
SAL0001284	hp	hypothetical protein	1	1	1	1	1	1	1	1	1	1	1	1	1	1	1	1	1	1	1	1	1	1	1	1	1	1	1	1	1	1	1	1	1	1	1	1	1	1	1	1	2
SAL0001285	group_699	phage resistance protein	1	1	1	1	1	1	1	2	1	1	1	1	1	1	1	1	1	1	1	1	1	1	1	1	1	1	1	1	1	1	1	1	1	1	1	1	1	1	1	1	1
SAL0001286	metQ_2	putative D-methionine-binding lipoprotein MetQ precursor	1	1	3	1	1	1	1	1	1	1	1	1	1	1	1	1	1	1	1	1	1	1	1	1	1	1	1	1	1	1	1	1	1	1	1	1	1	1	1	1	2
SAL0001287	group_701	Matrixin	1	1	1	1	1	1	1	1	1	1	1	1	1	1	1	1	1	1	1	1	1	1	1	1	1	1	1	1	1	1	1	1	1	1	1	1	1	1	1	1	2
SAL0001288	msrB	Peptide methionine sulfoxide reductase MsrB	1	1	1	1	1	1	1	1	1	1	1	1	1	1	1	1	1	1	1	1	1	1	1	1	1	1	1	1	1	1	1	1	1	1	1	1	1	1	1	1	2
SAL0001289	hp	hypothetical protein	1	1	1	1	1	1	1	1	1	1	1	3	1	1	1	1	1	1	1	1	1	1	1	1	1	1	1	1	1	1	1	1	1	1	1	1	1	1	1	1	2
SAL0001290	hp	hypothetical protein	1	1	1	1	1	1	1	1	1	1	1	1	1	1	1	1	1	1	1	1	1	1	1	1	1	1	1	1	1	1	1	1	1	1	1	1	1	1	1	1	2
SAL0001291	licC_2	Lichenan permease IIC component	1	1	1	2	1	1	1	1	1	1	1	1	1	1	1	1	1	1	1	1	1	1	1	1	1	1	2	1	1	1	1	1	1	1	1	1	1	1	1	1	3
SAL0001292	hp	hypothetical protein	1	1	2	1	1	1	1	3	1	1	1	1	1	1	2	1	1	1	1	1	1	1	1	1	1	1	1	1	2	1	2	1	2	1	1	1	1	1	1	1	1
SAL0001293	hp	hypothetical protein	1	1	1	1	1	1	1	1	1	1	1	1	1	1	1	1	1	1	1	1	1	1	1	1	1	1	1	1	1	1	1	1	1	1	1	2	1	1	1	1	1
SAL0001294	rsgA_1	Putative ribosome biogenesis GTPase RsgA	1	1	4	1	1	1	1	2	1	1	1	1	1	1	1	1	1	1	1	1	1	1	1	1	1	1	1	1	1	1	1	1	1	1	1	1	1	1	1	1	3
SAL0001295	thiN	Thiamine pyrophosphokinase	1	1	1	2	3	1	1	2	1	1	1	1	1	3	1	1	1	1	1	1	1	1	1	1	1	1	1	1	1	1	1	1	1	1	1	5	1	1	1	1	4
SAL0001296	hpt	Hypoxanthine-guanine phosphoribosyltransferase	2	2	2	2	2	2	2	2	2	2	2	2	2	2	2	2	2	2	2	2	2	2	2	2	2	2	2	2	2	2	2	2	3	2	2	2	2	2	2	2	4
SAL0001297	ybjI_4	Flavin mononucleotide phosphatase YbjI	1	1	1	1	1	1	1	1	1	1	1	1	1	1	1	1	1	1	1	1	1	1	1	1	1	1	1	1	1	1	1	1	1	1	1	1	1	1	1	1	2
SAL0001298	bmr3_2	Multidrug resistance protein 3	1	1	2	1	1	1	1	1	1	1	1	1	1	1	2	1	1	1	1	1	1	1	1	1	1	1	1	1	1	2	1	2	1	1	3	1	1	1	1	1	1
SAL0001299	group_714	putative oxidoreductase/MSMEI_2347	1	1	1	3	1	1	2	1	1	2	1	1	1	1	1	1	1	1	1	1	1	1	1	1	1	1	3	4	1	1	1	1	1	1	1	1	1	1	1	1	1
SAL0001300	group_715	Bacterial regulatory proteins, tetR family	1	1	1	1	1	1	1	3	1	1	1	1	1	1	1	1	1	1	1	1	1	1	1	1	1	1	1	1	1	1	1	1	1	1	1	1	1	1	1	1	2
SAL0001301	hp	hypothetical protein	1	1	1	1	1	1	1	1	1	1	1	1	1	1	1	1	1	1	1	1	1	1	1	1	1	1	1	1	3	1	1	1	1	1	1	1	1	1	1	1	2
SAL0001302	group_717	putative racemase	1	1	1	1	1	1	1	1	1	1	1	1	1	1	1	1	1	1	1	1	1	1	1	1	1	1	1	1	1	1	1	1	1	1	1	1	1	1	1	1	3
SAL0001303	hp	hypothetical protein	1	1	1	1	1	1	2	1	1	2	1	1	1	1	1	1	1	1	1	1	1	1	1	1	1	1	1	1	1	1	1	1	1	1	1	1	1	1	1	1	1
SAL0001304	slpA_5	S-layer protein precursor	1	1	1	1	1	1	1	1	1	1	1	1	1	1	1	1	1	1	1	1	1	1	1	1	1	1	1	2	1	1	1	1	1	1	1	1	1	1	1	1	3
SAL0001305	group_723	autoinducer-2 (AI-2) modifying protein LsrG	1	1	1	1	1	1	1	1	1	1	1	2	1	1	1	1	1	1	1	1	1	1	1	1	1	1	1	1	1	1	1	1	1	1	1	1	1	1	1	1	1
SAL0001306	hp	hypothetical protein	1	1	1	1	1	1	1	1	1	1	1	1	1	1	1	1	1	1	1	1	1	1	1	1	1	1	1	1	1	1	1	1	2	1	1	1	1	1	1	1	3
SAL0001307	hp	hypothetical protein	1	1	1	1	1	1	1	3	1	1	1	1	1	1	1	1	1	1	1	1	1	1	1	1	1	1	1	1	1	1	1	1	1	1	1	1	1	1	1	1	2
SAL0001308	pckA	Phosphoenolpyruvate carboxykinase [ATP]	1	1	2	3	1	1	1	2	1	1	1	4	1	1	2	1	1	1	1	1	1	1	1	1	1	1	3	2	1	2	1	2	1	1	1	1	1	1	1	1	1
SAL0001309	hp	hypothetical protein	1	1	4	1	1	1	1	3	1	1	1	1	1	1	1	1	1	1	1	1	1	1	1	1	1	1	1	1	1	1	1	1	1	1	1	1	1	1	1	1	2
SAL0001310	group_730	5-formyltetrahydrofolate cyclo-ligase family protein	1	1	1	1	1	1	1	2	1	1	1	1	1	1	1	1	1	1	1	1	1	1	1	1	1	1	1	1	1	1	1	1	1	1	1	1	1	1	1	1	3
SAL0001311	group_731	Bacterial membrane protein YfhO	1	1	1	1	1	1	2	5	1	2	1	1	1	1	4	1	1	1	1	1	1	1	1	1	1	1	1	1	1	1	1	1	1	1	1	1	1	1	1	1	3
SAL0001312	yodB	HTH-type transcriptional regulator YodB	1	1	2	1	1	1	4	2	1	5	1	6	1	1	2	1	1	1	1	1	1	1	1	1	1	1	1	2	1	2	1	2	1	1	1	1	1	1	1	1	3
SAL0001313	misCB	Membrane protein insertase MisCB precursor	1	1	1	1	1	1	1	4	1	1	1	3	2	1	1	1	1	1	1	1	1	1	1	1	1	1	1	1	1	1	1	1	1	1	1	1	1	1	1	1	5
SAL0001314	group_734	D,D-heptose 1,7-bisphosphate phosphatase	1	1	1	1	1	1	1	2	1	1	1	1	1	1	1	1	1	1	1	1	1	1	1	1	1	1	1	1	1	1	1	1	1	1	1	1	1	1	1	1	3
SAL0001315	add2	Aminodeoxyfutalosine deaminase	1	1	1	1	1	1	1	1	1	1	1	1	1	1	1	1	1	1	1	1	1	1	1	1	1	1	1	1	1	1	1	1	1	1	1	1	1	1	1	1	2
SAL0001316	mutM	Formamidopyrimidine-DNA glycosylase	1	1	1	1	1	1	1	1	1	1	1	1	1	1	1	1	1	1	1	1	1	1	1	1	1	1	1	2	1	1	1	1	1	1	1	1	1	1	1	1	3
SAL0001317	purM	Phosphoribosylformylglycinamidine cyclo-ligase	1	1	1	1	1	1	1	1	1	1	1	2	1	1	1	1	1	1	1	1	1	1	1	1	1	1	1	1	1	1	1	1	1	1	1	1	1	1	1	1	3
SAL0001318	purF	Amidophosphoribosyltransferase precursor	1	1	1	1	1	1	1	3	1	1	1	1	1	1	2	1	1	1	1	1	1	1	1	1	1	1	1	1	2	1	1	1	1	1	1	1	1	1	1	1	4
SAL0001319	group_739	Peptidase family M1	1	1	1	1	1	1	1	1	1	1	1	1	1	1	1	1	1	1	1	1	1	1	3	1	1	1	1	1	1	2	1	2	1	1	1	1	1	1	1	4	1
SAL0001320	mepA	Multidrug export protein MepA	1	1	1	1	1	1	1	3	1	1	1	1	1	1	2	1	1	1	1	1	1	1	1	1	1	1	1	3	1	2	1	2	1	1	1	1	1	1	4	1	5
SAL0001321	hp	hypothetical protein	1	1	3	1	1	1	1	2	1	1	1	1	1	1	1	1	1	1	1	1	1	1	1	1	1	1	1	1	1	1	1	1	1	1	1	1	1	1	1	1	1
SAL0001322	yhaM	3'-5' exoribonuclease YhaM	1	1	1	1	1	1	1	1	1	1	1	3	1	1	1	1	1	1	1	1	1	1	1	1	1	1	1	2	1	1	1	1	1	1	1	1	1	1	1	1	4
SAL0001323	lldD	L-lactate dehydrogenase [cytochrome]	1	1	1	1	1	1	1	1	1	3	1	2	1	1	1	1	1	1	1	1	1	1	1	1	1	1	1	1	1	1	1	1	1	1	1	1	1	1	1	1	1
SAL0001324	nnrD	ADP-dependent (S)-NAD(P)H-hydrate dehydratase	1	1	3	2	1	5	1	1	1	1	1	4	1	1	1	1	1	1	1	1	1	1	1	1	1	1	2	1	1	1	1	1	1	1	1	1	1	1	1	1	1
SAL0001325	group_745	MucBP domain protein	1	1	1	2	1	1	1	2	1	1	1	1	1	1	1	1	1	1	1	1	1	1	1	1	1	1	2	1	1	1	1	1	1	1	1	1	1	1	1	1	3
SAL0001326	group_746	Cupin domain protein	1	1	1	1	1	1	1	1	1	1	1	1	1	1	1	1	1	1	1	1	1	1	1	1	1	1	1	1	1	1	1	1	1	1	1	1	1	1	1	1	2
SAL0001327	cydA	Cytochrome bd ubiquinol oxidase subunit 1	1	1	1	3	1	1	1	1	1	1	1	1	1	1	2	1	1	1	1	1	1	1	1	1	1	1	1	1	1	2	1	2	1	1	1	1	1	1	1	1	1
SAL0001328	hp	hypothetical protein	1	1	1	2	1	1	1	1	1	1	1	3	1	1	1	1	1	1	1	1	1	1	1	1	1	1	1	1	1	1	1	1	1	1	1	1	1	1	1	1	1
SAL0001329	hp	hypothetical protein	2	2	1	1	1	1	1	1	1	1	2	1	1	1	1	1	1	1	1	2	2	1	1	1	1	1	1	1	1	1	2	1	1	1	1	1	1	1	1	2	3
SAL0001330	hp	hypothetical protein	1	1	2	2	1	2	2	2	1	2	1	2	1	1	2	1	1	1	1	1	1	1	1	1	1	1	2	2	2	2	1	2	1	1	1	1	1	1	1	1	3
SAL0001331	group_753	NADPH-dependent FMN reductase	1	1	1	1	1	1	1	2	1	1	1	1	1	1	1	1	1	1	1	1	1	1	1	1	1	1	1	1	1	1	1	1	1	1	1	1	1	1	1	1	3
SAL0001332	hp	hypothetical protein	1	1	1	1	1	1	1	2	1	1	1	1	1	1	1	1	1	1	1	1	1	1	1	1	1	1	1	2	1	1	1	1	1	1	1	1	1	1	1	1	3
SAL0001333	srrA_2	Transcriptional regulatory protein SrrA	1	1	1	1	1	1	1	1	1	1	1	1	1	1	1	1	1	1	1	1	1	1	1	1	1	1	1	1	1	1	1	1	1	1	1	1	1	1	1	2	3
SAL0001334	hp	hypothetical protein	1	1	1	1	1	1	1	2	1	1	1	1	1	1	1	1	1	1	1	1	1	1	1	1	1	1	1	1	1	1	1	1	1	1	1	1	1	1	1	1	1
SAL0001335	group_758	CAAX amino terminal protease self- immunity	1	1	1	1	1	1	1	2	1	1	1	3	1	1	1	1	1	1	1	1	1	1	1	1	1	1	1	2	1	1	1	1	1	1	1	1	1	1	1	1	4
SAL0001336	hp	hypothetical protein	1	1	1	1	1	1	1	1	1	1	1	1	1	1	1	1	1	1	1	1	1	1	1	1	1	1	1	1	1	1	1	1	1	1	1	1	1	1	1	1	2

SAL0001337	ssaB	Manganese ABC transporter substrate-binding lipoprotein precursor	1	1	1	1	1	1	1	5	1	1	1	1	1	1	2	1	1	1	1	1	1	1	1	1	1	1	1	1	1	2	1	2	1	1	1	1	1	1	1	1	3
SAL0001338	group_762	putative ABC transporter ATP-binding protein	1	1	2	1	1	1	1	2	1	1	1	1	1	1	2	1	1	1	1	1	1	1	1	1	1	1	1	2	1	2	1	2	1	1	1	1	1	1	1	1	3
SAL0001339	sufB	FeS cluster assembly protein SufB	1	1	1	1	1	1	1	1	1	1	1	1	1	1	1	1	1	1	1	1	1	1	1	1	1	1	1	1	1	1	1	1	1	1	1	1	1	1	1	1	2
SAL0001340	hp	hypothetical protein	1	1	1	1	1	1	2	3	1	2	1	2	1	1	1	1	1	1	1	1	1	1	1	1	1	1	1	1	1	1	1	1	1	1	1	1	1	1	1	1	4
SAL0001341	group_765	Putative NrdI-like protein	1	1	1	1	1	1	2	1	1	2	1	1	1	1	1	1	1	1	1	1	1	1	1	1	1	1	1	1	1	1	1	1	1	1	1	1	1	1	1	1	1
SAL0001342	hp	hypothetical protein	1	1	1	1	1	1	1	1	1	1	1	1	1	1	1	1	1	1	1	1	1	1	1	1	1	1	1	1	1	1	1	1	1	1	1	1	1	1	1	1	2
SAL0001343	gmk_2	Guanylate kinase	1	1	1	1	1	4	1	1	1	1	1	3	1	1	1	1	1	1	1	1	1	1	1	1	1	1	1	1	1	1	1	1	1	1	2	2	1	1	1	1	1
SAL0001344	mepS_2	Murein DD-endopeptidase MepS/Murein LD-carboxypeptidase precursor	1	1	1	1	1	1	1	1	1	2	1	1	1	1	1	1	1	1	1	1	1	1	1	1	1	1	1	1	1	1	1	1	1	1	1	1	1	1	1	1	3
SAL0001345	cap8A	Capsular polysaccharide type 8 biosynthesis protein cap8A	1	1	1	1	1	1	1	2	1	1	1	1	1	1	1	1	1	1	1	1	1	1	1	1	1	1	1	1	1	1	1	1	1	1	1	1	1	1	1	1	3
SAL0001346	fmnP	Riboflavin transporter FmnP	1	1	2	3	1	1	1	4	1	1	1	1	1	1	2	1	1	1	1	1	1	1	1	1	1	1	3	1	1	2	1	2	1	1	1	1	1	1	1	1	5
SAL0001347	wecA	UDP-N-acetylgalactosamine-undecaprenyl-phosphate N-acetylgalactosaminep	1	1	2	1	1	1	1	1	1	1	1	1	1	1	2	1	1	1	1	1	1	1	1	1	1	1	1	1	1	2	1	2	1	1	1	1	1	1	1	1	3
SAL0001348	gtaB_2	UTP--glucose-1-phosphate uridylyltransferase	1	1	1	1	1	1	1	1	1	1	1	1	1	1	1	1	1	1	1	1	1	1	1	1	1	1	1	1	1	1	1	1	1	1	1	1	1	1	1	1	2
SAL0001349	bglH_5	Aryl-phospho-beta-D-glucosidase BglH	1	1	1	3	1	1	2	4	1	2	1	1	1	1	1	1	1	1	1	1	1	1	1	1	1	1	1	1	1	1	1	1	1	1	1	1	1	1	1	1	5
SAL0001350	group_776	PTS system EIIBC component	1	1	1	1	1	1	1	1	1	1	1	1	1	1	1	1	1	1	1	1	1	1	3	1	1	1	1	1	4	1	1	1	1	1	1	1	1	1	1	1	2
SAL0001351	hp	hypothetical protein	1	1	1	1	1	1	1	1	1	1	1	1	1	1	1	1	1	1	1	1	1	1	1	1	1	1	1	1	1	1	1	1	1	1	1	1	1	1	1	1	2
SAL0001352	hp	hypothetical protein	1	1	1	1	1	1	1	1	1	1	1	1	1	1	1	1	1	1	1	1	1	1	1	1	1	1	1	1	1	1	1	1	1	1	1	1	1	1	1	1	2
SAL0001353	yxdM	ABC transporter permease protein YxdM	1	1	1	1	1	1	2	1	1	2	1	1	1	1	1	1	1	1	1	1	1	1	4	1	1	1	1	1	1	1	1	1	1	1	1	1	1	1	1	1	3
SAL0001354	group_780	multifunctional acyl-CoA thioesterase I and protease I and lysophospholipase	1	1	1	1	1	1	1	2	1	1	1	1	1	1	1	1	1	1	1	1	1	1	1	1	1	1	1	1	1	1	1	1	1	1	1	1	1	1	1	1	3
SAL0001355	group_782	Nicotinamide mononucleotide transporter	1	1	1	1	1	1	1	1	1	1	1	1	2	1	1	1	1	1	1	1	1	1	1	1	1	1	1	1	1	1	1	1	1	1	1	1	1	1	1	1	3
SAL0001356	hp	hypothetical protein	1	1	1	1	1	1	1	1	1	1	1	1	1	1	1	1	1	1	1	1	1	1	1	1	1	1	1	1	1	1	1	1	1	1	1	1	1	1	1	1	2
SAL0001357	hp	hypothetical protein	1	1	1	1	1	1	2	4	1	2	1	1	1	1	1	1	1	1	1	1	1	1	1	1	1	1	1	1	1	3	1	3	1	1	1	1	1	1	1	1	1
SAL0001358	group_786	ribosomal-protein-S5-alanine N-acetyltransferase	1	1	1	1	1	1	1	1	1	1	1	1	1	1	1	1	1	1	1	1	1	1	1	1	1	1	1	1	1	1	1	1	1	1	1	1	1	1	1	1	2
SAL0001359	mutL	DNA mismatch repair protein MutL	1	1	1	1	1	1	2	1	1	2	1	1	1	1	1	1	1	1	1	1	1	1	1	1	1	1	1	1	4	1	1	1	1	1	1	1	1	1	1	3	5
SAL0001360	dinB	DNA polymerase IV	2	2	2	2	2	2	2	2	2	2	2	2	2	2	2	2	2	2	2	2	2	2	2	2	2	2	2	2	2	2	2	2	2	2	2	2	2	2	2	2	2
SAL0001361	alaS	Alanine--tRNA ligase	1	1	1	1	1	1	1	1	1	1	1	1	1	1	1	1	1	1	1	1	1	1	1	1	1	1	1	1	3	1	1	1	1	1	1	1	1	1	1	2	1
SAL0001362	group_803	Non-canonical purine NTP pyrophosphatase	1	1	1	1	1	1	1	2	1	1	1	1	1	1	1	1	1	1	1	1	1	1	1	1	1	1	1	1	1	1	1	1	1	1	1	3	1	1	1	1	4
SAL0001363	yumC	Ferredoxin--NADP reductase 2	1	1	1	1	1	3	1	2	1	1	1	1	1	1	1	1	1	1	1	1	1	1	1	1	1	1	1	2	1	1	1	1	1	1	1	1	1	1	1	1	4
SAL0001364	group_812	Cysteine-rich secretory protein family protein	1	1	1	1	1	1	1	1	1	2	1	3	1	1	1	1	1	1	1	1	1	1	1	1	1	1	1	1	1	1	1	1	1	1	1	1	1	1	1	1	1
SAL0001365	yicI_1	Alpha-xylosidase	1	1	2	1	1	1	1	3	1	4	1	1	1	1	2	1	1	1	1	1	1	1	7	1	1	1	1	5	1	2	1	2	1	1	1	1	1	1	1	1	6
SAL0001366	group_814	PTS system galactitol-specific transporter subunit IIA	1	1	1	1	1	1	1	2	1	1	1	1	3	1	1	1	1	1	1	1	1	1	1	1	1	1	1	1	1	1	1	1	1	1	1	1	1	1	1	1	1
SAL0001367	phnE_1	Phosphate-import permease protein PhnE	1	1	2	1	1	2	2	2	1	2	1	2	1	1	2	1	1	1	1	1	1	1	1	1	1	1	1	2	2	2	1	2	1	1	1	1	1	1	1	3	2
SAL0001368	group_816	Calcineurin-like phosphoesterase superfamily domain protein	1	1	2	1	1	1	1	1	1	1	1	4	1	1	2	1	1	1	1	1	1	1	1	1	1	1	1	1	1	2	1	2	1	1	1	1	1	1	5	1	3
SAL0001369	hp	hypothetical protein	1	1	3	1	1	1	1	1	1	1	1	1	1	1	1	1	1	1	1	1	1	1	1	1	1	1	1	1	1	1	1	1	1	1	1	1	1	1	1	1	2
SAL0001370	group_818	multidrug resistance protein MdtH	1	1	1	1	1	1	1	1	1	1	1	1	1	1	1	1	1	1	1	1	1	1	1	1	1	1	1	1	1	1	1	1	1	1	1	1	1	1	1	2	3
SAL0001371	hp	hypothetical protein	1	1	1	1	1	1	1	1	1	1	1	1	1	1	1	1	1	1	1	1	1	1	1	1	1	1	1	1	1	1	1	1	1	1	1	2	1	1	1	1	3
SAL0001372	yycJ	Putative metallo-hydrolase YycJ	1	1	1	1	1	1	2	5	1	2	1	3	1	1	1	1	1	1	1	1	1	1	1	1	1	1	1	1	1	1	1	1	1	1	1	1	1	1	1	1	4
SAL0001373	rlmH	Ribosomal RNA large subunit methyltransferase H	1	1	1	1	1	1	1	2	1	1	1	1	1	1	1	1	1	1	1	1	1	1	1	1	1	1	1	1	1	1	1	1	1	1	1	1	1	1	1	1	3
SAL0001374	group_822	Helix-turn-helix domain protein	1	1	1	2	1	1	1	1	1	1	1	1	1	1	1	1	1	1	1	1	1	1	1	1	1	1	1	1	1	1	1	1	1	1	1	1	1	1	1	1	3
SAL0001375	hp	hypothetical protein	1	1	1	1	1	1	2	1	1	2	1	3	1	1	1	1	1	1	1	1	1	1	1	1	1	1	1	1	1	1	1	1	1	1	1	1	1	1	1	1	1
SAL0001376	hp	hypothetical protein	1	1	1	1	1	1	1	1	1	1	1	1	1	1	1	1	1	1	1	1	1	1	1	1	1	1	1	1	1	1	1	1	1	1	1	1	1	1	1	3	2
SAL0001377	rnhA	Ribonuclease H	1	1	1	2	1	1	1	1	1	1	1	1	1	1	1	1	1	1	1	1	1	1	1	1	1	1	2	1	1	1	1	1	1	1	1	1	1	1	1	1	3
SAL0001378	hp	hypothetical protein	1	1	1	1	1	1	1	1	1	1	1	1	1	1	1	1	1	1	1	1	1	1	1	1	1	1	1	1	1	1	1	1	1	1	1	1	1	1	1	1	2
SAL0001379	hp	hypothetical protein	1	1	1	1	1	1	1	1	1	1	1	2	1	1	1	1	1	1	1	1	1	1	1	1	1	1	1	1	1	1	1	1	1	1	1	1	1	1	1	1	3
SAL0001380	iphP_3	Tyrosine-protein phosphatase precursor	1	1	1	1	1	1	1	1	1	1	1	1	1	1	1	1	1	1	1	1	1	1	1	1	1	1	1	1	1	1	1	1	1	1	1	1	1	1	1	2	3
SAL0001381	group_831	Glycosyl transferase family 8	1	1	1	1	1	1	1	1	1	1	1	2	1	1	1	1	1	1	1	1	1	1	1	1	1	1	1	1	1	1	1	1	1	1	1	1	1	1	1	1	3
SAL0001382	gltC	HTH-type transcriptional regulator GltC	1	1	1	1	1	1	1	1	1	1	1	1	1	1	1	1	1	1	1	1	1	1	1	1	1	1	1	1	1	1	1	1	1	1	1	1	1	1	1	1	2
SAL0001383	group_848	Aluminium resistance protein	1	1	1	2	1	1	1	1	1	1	1	1	1	1	1	1	1	1	1	1	1	1	4	1	1	1	2	1	1	1	1	1	1	1	1	1	1	1	1	1	3
SAL0001384	group_850	YbbR-like protein	1	1	1	1	1	1	1	2	1	1	1	1	1	1	1	1	1	1	1	1	1	1	1	1	1	1	1	1	1	1	1	1	1	1	1	1	1	1	1	1	3
**SAL0001385**	**uvrA_2**	**UvrABC system protein A**	1	1	1	2	1	1	1	1	1	1	1	1	1	1	1	1	1	1	1	1	1	1	1	1	1	1	2	1	3	1	1	1	1	1	1	4	1	1	1	1	5
SAL0001386	group_854	CAAX amino terminal protease self- immunity	1	1	1	1	1	1	1	3	1	1	1	1	1	1	1	1	1	1	1	1	1	1	1	1	1	1	1	1	1	1	1	1	1	1	1	2	1	1	1	1	4
SAL0001387	hp	hypothetical protein	1	1	5	1	1	7	1	4	1	1	1	8	1	1	2	1	1	1	1	1	1	1	1	1	1	1	6	3	1	2	1	2	1	1	1	1	1	1	1	1	9
SAL0001388	metA	Homoserine O-succinyltransferase	1	1	1	1	1	1	1	4	1	1	1	1	1	1	3	1	1	1	1	1	1	1	1	1	1	1	1	1	1	2	1	2	1	1	1	1	1	1	1	1	5
SAL0001389	hp	hypothetical protein	1	1	1	2	1	3	1	5	1	1	1	4	1	1	1	1	1	1	1	1	1	1	1	1	1	1	2	1	1	1	1	1	1	1	1	1	1	1	1	1	1
**SAL0001390**	**phoU**	**Phosphate-specific transport system accessory protein**	1	1	1	1	1	1	1	2	1	5	1	1	1	1	1	1	1	1	1	1	1	1	1	1	1	1	1	4	1	1	1	1	1	1	1	1	1	1	1	1	3
Sequence type			1	2	3	4	5	6	7	8	9	10	11	12	13	5	14	9	15	16	17	18	19	20	21	22	23	9	24	25	26	27	19	27	28	29	30	31	29	9	32	33	34

The bold letters indicate the genes used for validation of reference strains.

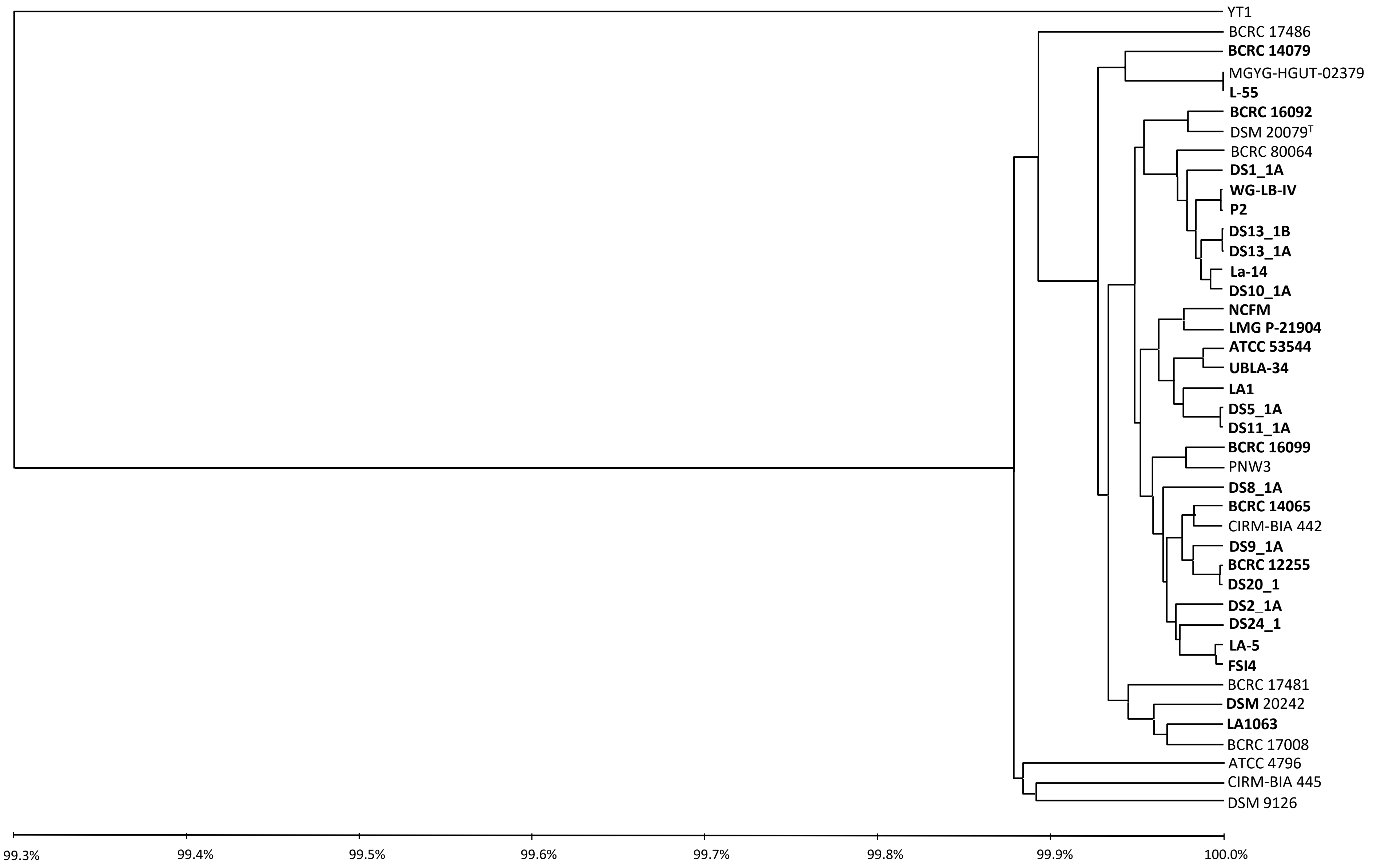

Supplementary Fig. S1. Unweighted pair group method with arithmetic mean (UPGMA) dendrogram based on OrthoANI values among the 41 *Lactobacillus acidophilus* strains.

(a)

```
                   Rex2-F →
Reference strains  AGGTAAACGCGGATATGGATATGATGTTAAAAATTTGCTGAGTTTCTTTAAGAAAATTTT
LA1063/BCRC 17481  AGGTAAACGCGGATATGGATATGATGTTAAAAATTTGCTGAGTTTCTTTAAGAAAATTTT
                   ************************************************************

Reference strains  GAATCAAGATACACTAACTAATGTTGCGTTAATTGGTGTTGGTAACTTAGGACGAGCACT
LA1063/BCRC 17481  GAATCAAGATACACTAACTAATGTTGCGTTAATTGGTGTTGGTAACTTAGGACGAGCACT
                   ************************************************************

Reference strains  TTTAAATTATAATTTTAAGCGTAGCAACAATATCCGTATTTCTTGTGCATTTGATATTAA
LA1063/BCRC 17481  TTTAAATTATAATTTTAAGCG---------------------------------------
                   *********************

Reference strains  CAAAGAAATTACTGGTCGAATTTTAAGCGGTGTTCCCGTTTATGATATGGAAGATTTAAA
LA1063/BCRC 17481  -----------------------------GTGTTCCCGTTTATGATATGGAAGATTTAAA
                                                *******************************
                                                              ← Rex2-R
Reference strains  ACAACAATTAAGTGATCAACAAATTTCGATTGCTATTTTAACAGTTCCTTCGACTGCGGC
LA1063/BCRC 17481  ACAACAATTAAGTGATCAACAAATTTCGATTGCTATTTTAACAGTTCCTTCGACTGCGGC
                   ************************************************************

Reference strains  T
LA1063/BCRC 17481  T
                   *
```

(b)

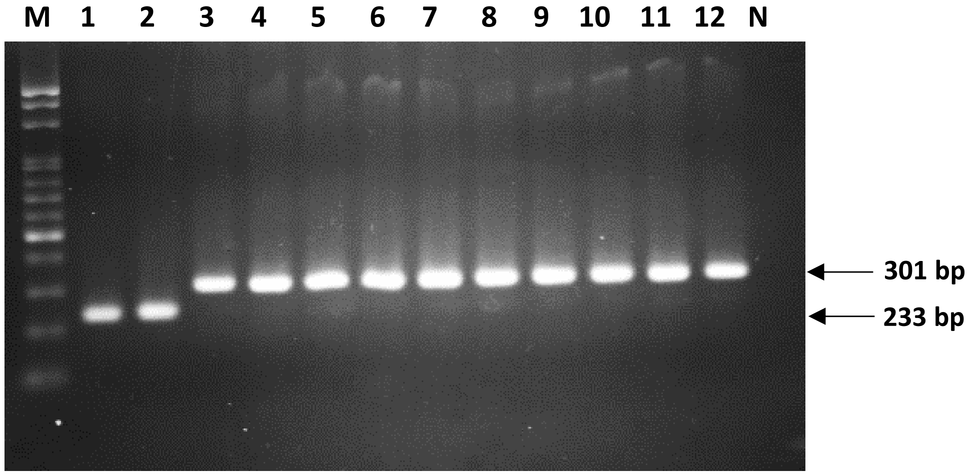

Supplementary Fig. S2. Alignment of *Rex2* gene sequences among the 11 *Lactobacillus acidophilus* strains reference strains and LA1063, differs in a 68-bp insertion/deletion. (a) The specific primer regions are indicated in bold letters with underlines. (b) PCR products amplified from genomic DNA of *L. acidophilus* strains. Template DNA was amplified with the strain-specific primers Rex2-F/R. Lanes M, 100-bp DNA size marker; 1, LA1063; 2, BCRC 17481; 3, BCRC 10695^{T}; 4, BCRC 12255; 5, BCRC 14065; 6, BCRC 14079; 7, BCRC 16092; 8, BCRC 16099; 9, BCRC 17008; 10, BCRC 17009; 11, BCRC 17488; 12, BCRC 80064; N, negative.

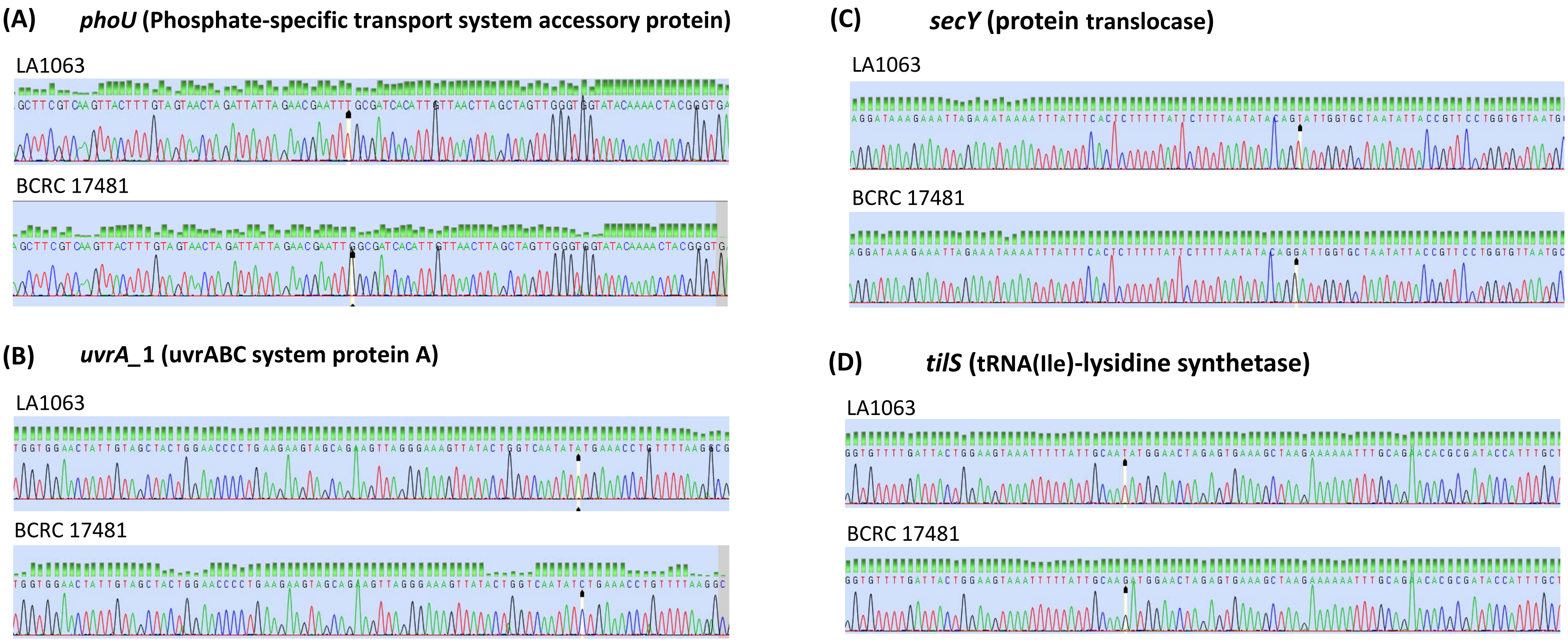

Supplementary Fig. S3. Discriminated loci between *Lactobacillus acidophilus* LA1063 and BCRC 17481. (A) *phoU*: T/G; (B) *uvrA*_1: A/C; (C) *secY*: T/G; (D) *tilS*: T/G. The single nucleotide polymorphism site is marked with an arrow.
